# Ir(III) Compounds Containing a Terdentate Ligand Are Potent Inhibitors of Proliferation and Effective Antimetastatic Agents in Aggressive Triple-Negative Breast Cancer Cells

**DOI:** 10.1021/acs.jmedchem.3c00586

**Published:** 2023-07-06

**Authors:** Vojtech Novohradsky, Alicia Marco, Lenka Markova, Natalia Cutillas, José Ruiz, Viktor Brabec

**Affiliations:** † Institute of Biophysics, Czech Academy of Sciences, Kralovopolska 135, Brno CZ-61200, Czech Republic; ‡ Departamento de Química Inorgánica, Universidad de Murcia and Institute for Bio-Health Research of Murcia (IMIB-Arrixaca), Murcia E-30100, Spain

## Abstract

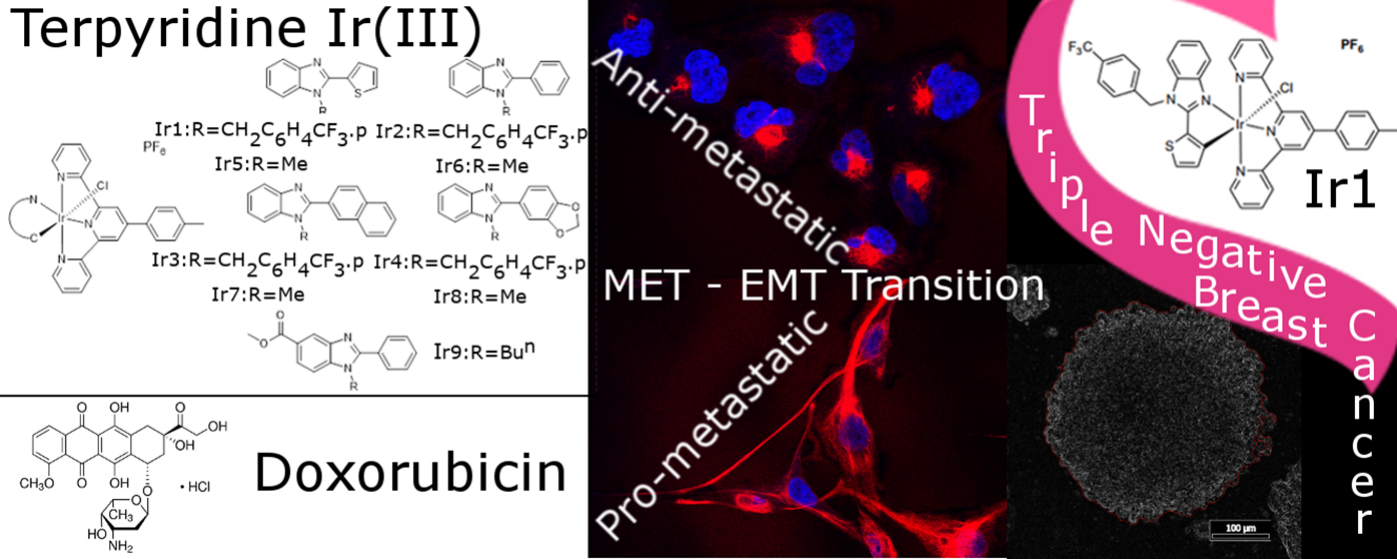

Herein, we report a series of new octahedral iridium(III) complexes **Ir1**–**Ir9** of the type [Ir(N^N^N)(C^N)Cl]PF_6_ (N^N^N = 4′-(*p*-tolyl)-2,2′:6′,2″-terpyridine; C^N = deprotonated 2-arylbenzimidazole backbone) to introduce new metal-based compounds for effective inhibition of metastatic processes in triple-negative breast cancer (TNBC). The results show that the structural modifications within the C^N scaffold strongly impact the antimetastatic properties of these complexes in TNBC cells. Furthermore, testing the antimetastatic effects of the investigated Ir complexes revealed that the highest antimetastatic activity in TNBC cells is exhibited by complex **Ir1**. This result was in contrast to the effects of the clinically used drug doxorubicin used in conventional chemotherapy of TNBC, which conversely promoted metastatic properties of TNBC cells. Thus, the latter result suggests that doxorubicin chemotherapy may increase the risk of metastasis of breast cancer cells, so the search for new drugs to treat breast cancer that would show better antitumor effects than doxorubicin is justified.

## Introduction

Despite the application potential of platinum drugs in various anticancer chemotherapeutic regimens, they are rarely active against tumor metastases, even in the combined treatment schedules with other drugs. This is counterproductive since metastases account for over 90% of cancer deaths.^1^ Moreover, most solid primary tumors are curable with surgery and radiation therapy; chemotherapy and immunotherapy are applied for a complete cure if the tumor metastasizes. Therefore, the need for antimetastatic targeted chemotherapy rather than just the chemotherapeutic targeting of primary tumors is coming to the fore.^1,2^

Despite the large variety of metal-based compounds already demonstrated to have anticancer activity, the antimetastatic properties of this class of antitumor drugs were investigated much less. The metal-based compounds screened for their antimetastatic activity include those containing cobalt, copper, gold, gadolinium, nickel, palladium, platinum, ruthenium, and vanadium.^2,3^ It is worth noting that two ruthenium compounds, RAPTA-C and NAMI-A, were recognized and studied extensively for their antimetastatic properties *in vitro* and *in vivo*. Surprisingly, the antiproliferative activity of NAMI-A in cancer cells is far worse compared to conventional cisplatin. On the other hand, the ability of NAMI-A to inhibit the main steps of the metastatic process (detachment from the primary tumor, migration, invasion, and re-adhesion) is the most beneficial in antitumor treatment with this drug. Thus, searching for compounds showing both antiproliferative and antimetastatic activities seems to be a very efficient strategy against neoplasms with metastatic potential. Also notably, recently, a few papers were published describing the antimetastatic potential of Ir complexes.^4−8^

One of the most common cancers where metastases are responsible for most deaths is breast cancer,^9,10^ in particular triple-negative breast cancer (TNBC), an aggressive subtype of breast cancer with higher mortality rate. The most effective chemotherapy drug for breast cancer is currently doxorubicin.^11^ However, although doxorubicin is a potent therapeutic agent, it has adverse side effects, such as cardiotoxicity, myelosuppression, and palmar-plantar erythrodysesthesia,^12−14^ and the clinical data demonstrate reduced progression-free and overall survival of patients treated even with a low dose of doxorubicin.^15^ Thus, chemotherapy of breast cancer patients by doxorubicin, on the one hand, kills primary cancer cells, whereas, on the other hand, it promotes breast cancer cell metastasis.^16^ New chemotherapeutics capable of effectively treating breast cancer should therefore show the ability to simultaneously kill primary cancer cells and inhibit metastatic processes.

Recently, cationic heteroleptic bis-cyclometalated Ir(III) complexes have received enormous attention due to their excellent anticancer activity with non-conventional modes of action and precise subcellular localization.^17−23^ However, most complexes with bidentate ligands often exist as a mixture of enantiomers,^24^ which may bring unanticipated side effects. The incorporation of tridentate ligands can avoid chirality, as in the cases of recently reported iridium photocatalysts of the types [Ir(N^N^N)(C^N)Cl]^+^, [Ir(N^N^N)(C^N^C)]^+^, [Ir(N^N^N)_2_]^3+^, and [Ir(N^N^N))(C^N))Cl]_2_^2+^.^25−28^

**Scheme 1:**
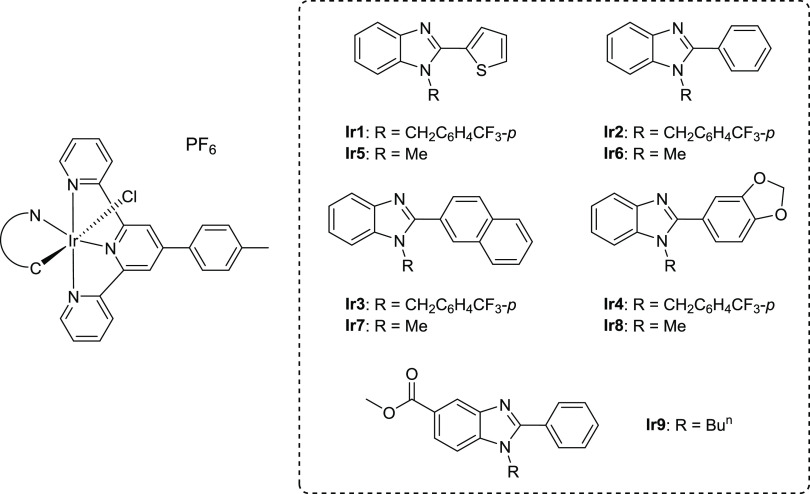
Structures of the New Terpyridine Iridium Tested Compounds

Herein, we have synthesized a family of new complexes of the type [Ir(ttpy)(C^N)Cl]^+^, where “ttpy” represents 4′-(*p*-tolyl)-2,2′:6′,2″-terpyridine and “C^N” stands for different C,N-donor ligands containing the deprotonated 2-arylbenzimidazole backbone (Scheme 1). 2-(Aryl)benzimidazole was selected as the scaffold for the C^N ligands to investigate their antiproliferative and antimetastatic effects in TNBC cells and thus find a relationship between their chemical structure and biological activity. Additionally, in one case, an ester functionality was installed as a handle for the intended functionalization of metallodrugs.^29−31^

Thus, this work aimed to develop a new metal-based compound(s) for the effective treatment of TNBC that would overcome drawbacks associated with treating this type of cancer with doxorubicin used in the conventional chemotherapy of TNBC.

## Results and Discussion

### Synthesis and Characterization of Proligands (**HL1**–**HL8**) and Ir(III) Complexes (**Ir1**–**Ir9**)

**Scheme 2:**
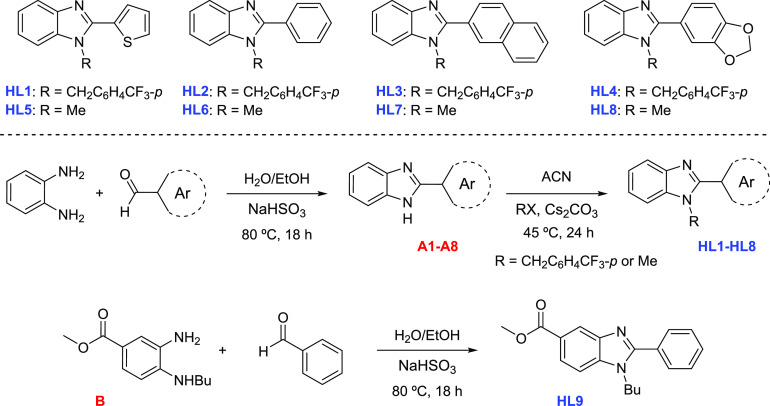
Synthesis of Proligands for SAR **HL1**–**HL9**

Precursors 2-(aryl)benzimidazole **A1**–**A8** (Scheme 2) were synthesized via condensation reactions between *o*-phenylenediamine and the corresponding aldehyde by adaptation of procedures reported in the literature,^32,33^ whereas the synthesis of the proligands **HL1**–**HL8** was performed from the corresponding 2-(aryl)benzimidazole precursor (**A1**–**A8**) by reaction with MeI or 4-(trifluoromethyl)benzyl bromide (R-Br). On the other hand, the synthesis of **HL9** was achieved by condensing the key intermediate diamine **B** with benzaldehyde (Scheme 2, bottom).^29^ The NMR spectra of the new proligand **HL4** are shown in Figures S1 and S2.

**Scheme 3:**
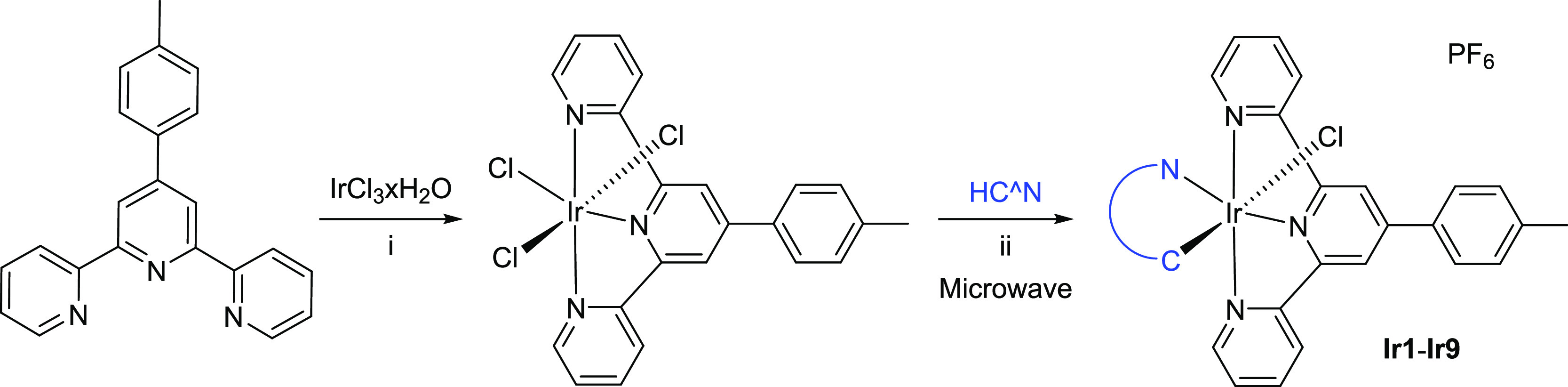
Synthesis of Iridium Complexes **Ir1**–**Ir9** Investigated in This Work (i) IrCl_3_×H_2_O and ttpy in ethylene glycol at 180 °C for 25 min; (ii) precursor complex and the corresponding HC^N proligand and KPF_6_ in ethylene glycol at 240 °C for 12 min in a reaction microwave.

The synthesis of complexes **Ir1**–**Ir9** from Ir(ttpy)Cl_3_ required harsh reaction conditions (240 °C) to overcome the inertness of the coordination sphere (Scheme 3).

The new orange air-stable iridium complexes **Ir1–Ir9** (Scheme 1) were obtained in good yields as PF_6_ salts. All complexes were characterized using high-resolution ESI(+)/MS spectrometry, multinuclear NMR spectroscopy (see Figures S1–S67 in the Supporting Information), and elemental analysis. The ^1^H NMR spectra of all complexes in DMSO-*d*_6_ show the aromatic hydrogen peaks from 6 to 9.5 ppm, whereas the characteristic signal of the methyl group of the ttpy ligand around 2.5 ppm could be overlapped with the DMSO-*d*_6_ signal. The benzyl derivatives **Ir1**–**Ir4** also show a singlet due to CH_2_ around 6 ppm, whereas the methyl derivatives **Ir5**–**Ir8** exhibited a singlet around 4.5 ppm. **Ir9** also showed the characteristic signals of the ester group. The ^19^F NMR spectra also confirmed the formation and purity of the complexes. Thus, a singlet at approximately −63 ppm for the CF_3_ group of complexes containing a fluorinated C^N ligand, with the additional expected doublet due to PF_6_^–^ in complexes with this counter anion, with the correct −C–F vs PF_6_^–^ ratio. The ESI-MS spectra displayed the [M-PF_6_]^+^ peaks (Figures S68–S77). The purity of complexes was higher than 95% through high-performance liquid chromatography (HPLC) analysis (HPLC traces in Figures S78 and S79) and NMR.

**Figure 1:**
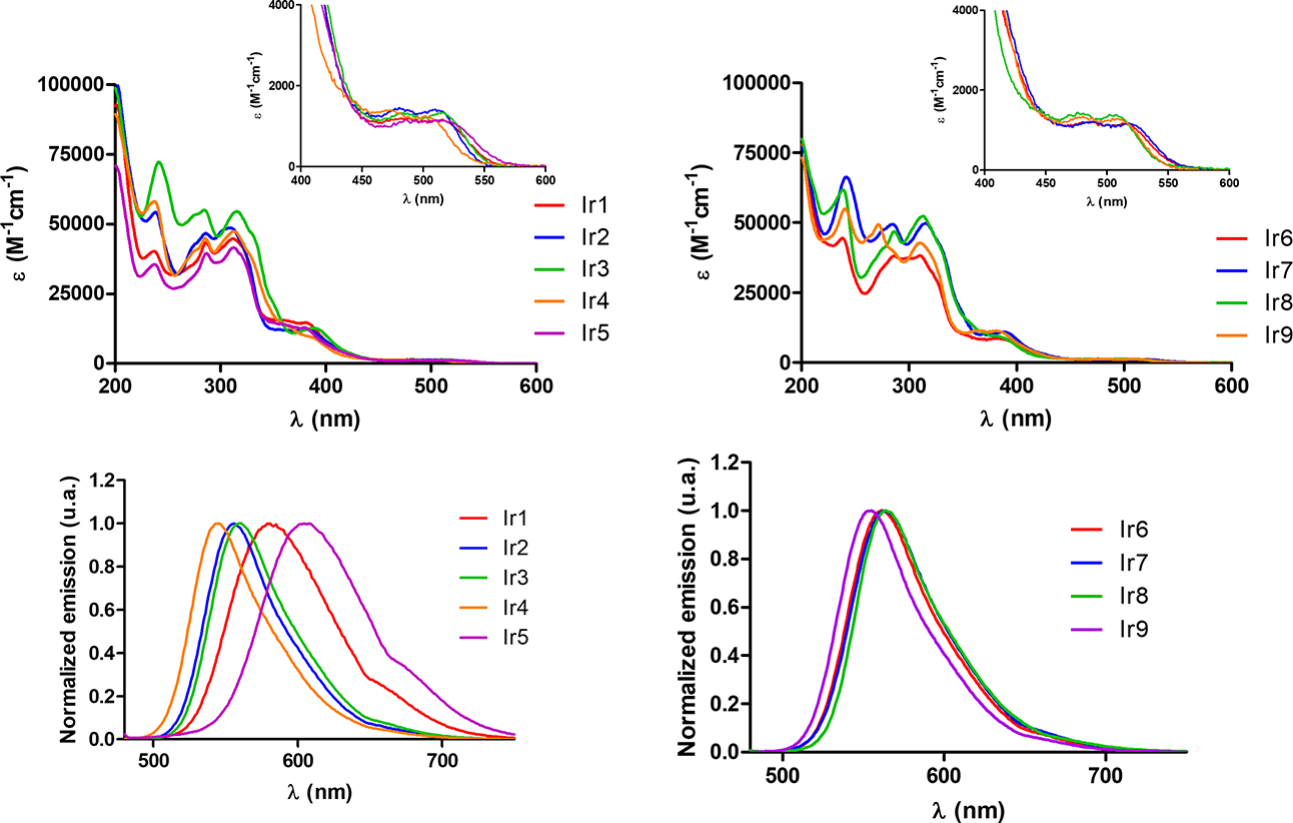
UV/Vis spectra (top) and normalized emission spectra (bottom) of **Ir1**–**Ir9** in aerated acetonitrile (λ_exc_ = 405 nm, 10 μM). The UV/Vis spectral insets show an expansion of the 400–600 nm region.

The UV/Vis absorption and emission spectra of **Ir1**–**Ir9** were measured in acetonitrile and water (1% DMSO) (Figure 1 and Figure S80, respectively) at room temperature. All complexes showed intense high-energy absorption bands in the 250–350 nm range due to π–π* electronic transitions located on the terpyridine and cyclometalating ligands. The broad bands at 350–430 nm could be attributed to the metal-to-ligand charge transfer (MLCT) and ligand-to-ligand charge transfer (LLCT) transition. In contrast, the weak absorption shoulders at ∼450 nm are considered spin-forbidden ^3^MLCT/LLCT transition as a consequence of the spin–orbit coupling of an Ir(III) heavy atom (ζ = 3909 cm^–1^),^34^ which allows for fast and efficient intersystem crossing (ISC) to convert singlet excitons to triplets.^26,35−38^ The triplet nature of these excited states could make them appropriate for bioimaging and photodynamic therapy.^39^ As observed, subtle structural modifications of the C^N ligand only moderately affected the UV/Vis absorption spectra of the corresponding Ir(III) complexes in both acetonitrile and water. It is interesting to note that upon excitation at 405 nm, complexes showed yellow to red emissions in both aerated acetonitrile and water (1% DMSO) (Figure 1 and Figure S80, respectively), with **Ir1** and **Ir5** showing the highest emission wavelengths in aerated acetonitrile, probably due to the presence of the thiophen-2-yl group in the C^N ligand. Table S1 lists some optical properties of the new iridium complexes in deaerated acetonitrile. The emission lifetimes for these complexes at room temperature were in the range of 0.34–5.99 μs, which is in the order of the values observed in the literature.^26^ As shown, complexes **Ir2** and **Ir9** exhibited quantum phosphorescence yields higher than 70%, whereas for **Ir1** and **Ir5**, the quantum yields were less than 10% in the same conditions. As shown in Figure S81, some quenching is observed for complex **Ir1** (λ_exc_ = 355 nm) both in water and PBS. Finally, it is worth noting that the maximum emission wavelengths of complex **Ir1** in different solvents gradually increase according to the order DCM < ACN < DMSO < H_2_O (Figure S82), i.e., by increasing the polarity of the solvents, suggesting the existence of a solvatochromic effect.

### Stability Studies

**Figure 2:**
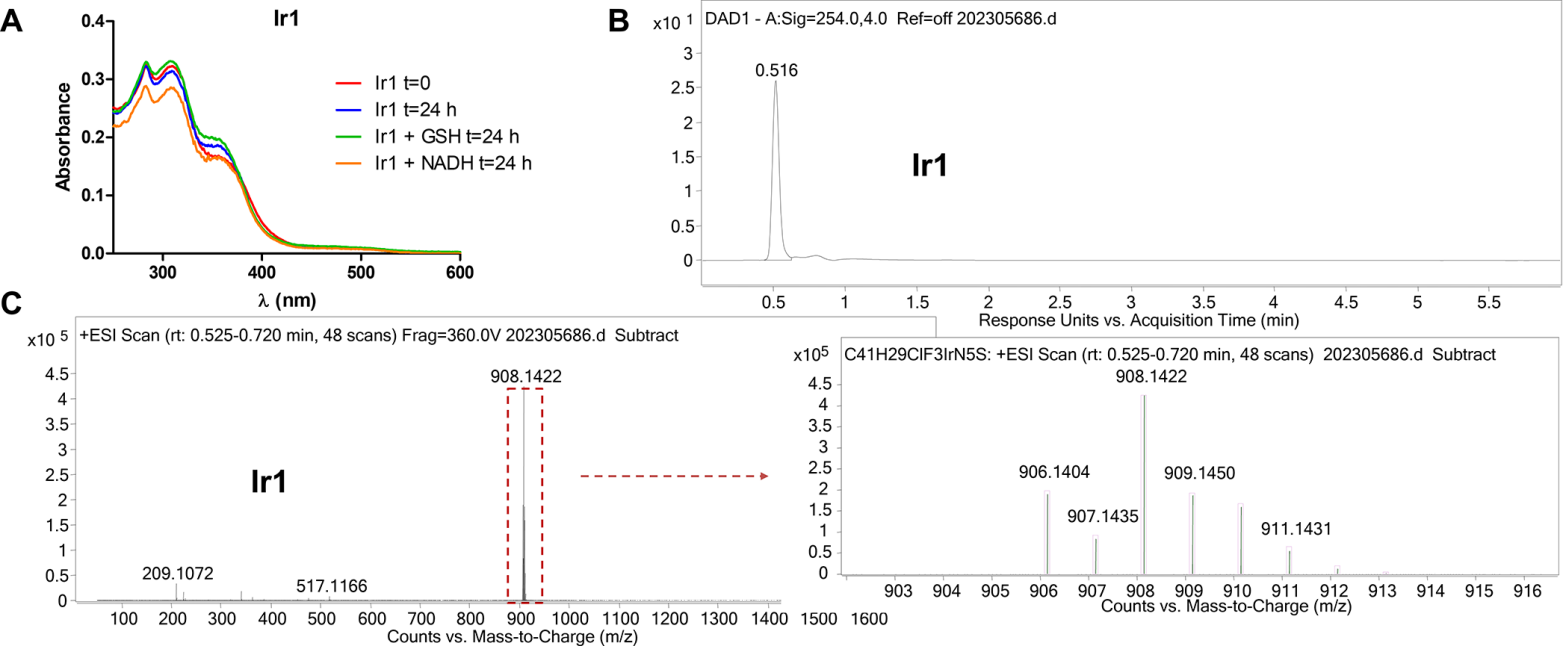
(A) Time evolution of the absorbance spectrum of complex **Ir1** (10 μM) in water (1% DMSO) in the absence and presence of an excess of GSH (10 mM) or NADH (100 μM). (B) HPLC chromatogram of complex **Ir1** (UV detection at 310 nm) using acetonitrile:water (80:20) as a mobile phase in isocratic mode (0.1% formic acid) and (C) the corresponding mass spectra showing the [M-PF_6_]^+^ peak.

The stability of the complexes **Ir1**–**Ir9** was studied in DMSO and RPMI (5% DMSO) using UV/Vis spectroscopy at different times (Figures S83–S85). The spectra remained constant in these conditions, suggesting that the investigated complexes are stable in DMSO or cell culture media. In addition, to further mimic the cellular and physiological conditions, the stability of the new complexes was also tested in the presence of 10 mM GSH or 100 μM NADH in water (1% DMSO) using UV/Vis spectroscopy (Figure 2A for **Ir1** and Figures S85–S88 for **Ir2**–**Ir9**). As shown, no changes in the shapes of the peaks were observed. In addition, further evidence of the stability of the complexes toward hydrolysis came from the HPLC studies when using acetonitrile:water (80:20) as a mobile phase in isocratic mode. As shown in Figure 2B for complex **Ir1**, only one peak was observed in the chromatogram (UV detection at 310 nm), which according to the mass spectra of the peak of interest extracted from the chromatogram (Figure 2C), corresponded to the adduct [M-PF_6_]^+^, indicating the strength of the Ir–Cl bond. Similar results were found for other selected complexes (Figure S86 for **Ir2**, **Ir7**, and **Ir8**).

### Antiproliferative Activity

One of the first steps in identifying new antitumor chemotherapeutics is an antiproliferative screening of candidate molecules *in vitro*. The antiproliferative activity of the investigated Ir(III) complexes containing a terdentate ligand was determined against the model human cancer cell lines, namely, the highly aggressive TNBC MDA-MB-231 cells and poorly aggressive and non-invasive MCF-7 breast cancer cells and, for comparative purposes, also a non-malignant breast MCF-10A cell line derived from the same epithelial origin. The IC_50_ values (IC_50_ is defined as the concentration of the agent inhibiting cell growth by 50%) determined against MDA-MB-231 cells ranged from 0.7 μM (compound **Ir4**) up to 3.9 μM (compound **Ir7**) with the mean IC_50_ value over the nine investigated Ir(III) complexes of 2.0 μM. Screening the antiproliferative activity against MCF-7 cells revealed the lowest IC_50_ value (1.1 μM) after the treatment with compound **Ir1** and the highest (5.0 μM) after the treatment with compound **Ir6**. The overall mean IC_50_ determined for all nine investigated Ir(III) compounds against the MCF-7 cell line was 2.3 μM.

The conventional chemotherapy for breast cancer includes doxorubicin.^9^ Thus, conventional doxorubicin was used in this study as well as the control for comparative purposes. The data in Table 1 show that the most effective Ir complex **Ir4** yielded the IC_50_ value comparable to that obtained for the treatment with doxorubicin, but only when MDA-MB-231 cells were treated. If MDA-MB-231 cells were treated with other investigated Ir complexes, **Ir1**–**Ir3** and **Ir5**–**Ir9**, these complexes were somewhat less effective than doxorubicin (IC_50_ values were 1.6–6.3-fold higher than that obtained for doxorubicin). Similarly, in the case of the treatment of MCF-7 cells, all investigated Ir complexes **Ir1**–**Ir9** were less effective than doxorubicin (IC_50_ values were 3.3–15.2-fold higher than that obtained for doxorubicin).

**Table 1:** IC_50_ Values (Mean ± SD, μM)^a^ Determined for the Investigated Ir Complexes and Doxorubicin by the Sulforhodamine B (SRB) Assay after 72 h of Incubation

IC_50,72h_ (μM)^a^	MDA-MB-231	MCF-7	MCF-10A	SI
**Ir1**	1.5 ± 0.2	1.1 ± 0.1	32 ± 5	25
**Ir2**	1.3 ± 0.1	2.1 ± 0.2	28 ± 4	16
**Ir3**	2.9 ± 0.4	2.7 ± 0.1	17 ± 2	6
**Ir4**	0.7 ± 0.1	1.1 ± 0.2	25 ± 2	28
**Ir5**	1.0 ± 0.2	2.9 ± 0.2	27 ± 2	14
**Ir6**	1.7 ± 0.4	5.0 ± 0.6	25 ± 3	8
**Ir7**	3.9 ± 0.6	2.0 ± 0.3	31 ± 5	10
**Ir8**	1.4 ± 0.4	1.2 ± 0.1	27 ± 5	20
**Ir9**	3.2 ± 0.2	2.4 ± 0.4	24 ± 4	8
Dox	0.62 ± 0.08	0.33 ± 0.07	8.6 ± 0.8	18

a Data represent the mean ± SD from at least three independent experiments. SI, average selectivity index calculated as IC_50_ (MCF-10A non-cancerous cells)/average IC_50_ (MDA-MB-231 and MCF-7 cancer cells). Data from the antiproliferative activity were subjected to detailed statistical analysis using ANOVA tests, and the data are listed in the Supporting Information (Tables S2–S5).

We evaluated the IC_50,72h_ values in Table 1 to determine the statistically significant differences among these values for complexes **Ir1**–**Ir9** and doxorubicin used for the treatment of three cell lines MDA-MB-231, MCF-7, and MCF-10A using ANOVA tests. The results are summarized in the Supporting Information (Tables S2–S5). Based on the evaluation of the *P* values, it is reasonable to suggest that there are no statistically significant differences among the IC_50,72h_ values obtained for complexes **Ir1**–**Ir9** and doxorubicin in two tumor cell lines (MDA-MB-231 vs MCF-7). In contrast, a statistically significant difference exists between the *P* values obtained for each tumor and noncancerous cell line (MDA-MB-231 vs MCF-10A and MCF-7 vs MCF-10A). However, from the results of comparing the IC_50,72h_ obtained for the individual investigated compounds with each other in the individual cell lines (Tables S3–S5), no clear generalizing conclusion can be made about the significance of the differences between the antiproliferative efficiencies of the individual investigated compounds.

Another parameter predicting effective and potentially non-toxic anticancer chemotherapy is the selectivity index (SI), which represents the ratio of the IC_50_ determined for the non-cancerous cell line over the IC_50_ determined for cancerous cell lines. We calculated the selectivity indices defined as IC_50_ (MCF-10A)/average IC_50_ (MDA-MB-231 and MCF-7 cancerous cell lines). The data showed a promising mean selectivity index for compounds **Ir1** and **Ir4** (SI ∼ 25 and 28, respectively). In both cases, **Ir1** and **Ir4** have considerably higher selectivity indices than the conventional control compound doxorubicin (SI ∼ 18). In summary, compounds **Ir1** and **Ir4** show good activity against MDA-MB-231 and MCF-7 cells, with distinct selectivity to these breast cancer cells.

### Accumulation and Localization in Cells

The biological effects of antitumor Ir compounds depend on their ability to accumulate inside cells. Therefore, to obtain some basic information about the mechanisms responsible for the antiproliferative activity of the Ir complexes investigated in this work, we determined the Ir content inside MDA-MB-231 cells treated with the equimolar concentrations of tested compounds by ICP/MS. It is important to mention that treatment of MDA-MB-231 cells with Ir complexes at the concentrations used in these experiments did not result in elevation of the number of dead cells. The viability of treated cells ranged from 93 to 97%, so the results were unaffected by the increased permeability of damaged cell membranes of dying/dead cells. Moreover, the log *P* values of Ir complexes were also measured to determine whether there is a correlation between the lipophilicity and intracellular accumulation of the investigated Ir compound (Table 2).

**Table 2:** Amount of Iridium Taken Up by MDA-MB-231 Cells within 24 h (at 1 μM Complex Concentration) and Log *P* Values of the Investigated Complexes

complex	cellular uptake (ng Ir/10^6^ cells)	log *P*^a^
**Ir1**	35 ± 1	0.42
**Ir2**	39 ± 2	0.65
**Ir3**	60 ± 2	0.95
**Ir4**	73 ± 3	0.83
**Ir5**	39 ± 4	0.40
**Ir6**	27 ± 1	0.46
**Ir7**	43 ± 5	0.77
**Ir8**	53.3 ± 0.3	0.76
**Ir9**	54 ± 3	0.85

a Log *P* (octanol/water) values for the tested platinum compounds determined by the “shake-flask” method.

The amount of iridium taken up by MDA-MB-231 cells incubated with Ir complexes **1**–**9** correlated with their lipophilicity (log *P* values) (Table 2) (Spearman’s correlation coefficient = 0.87). These data indicate that the ability of the investigated Ir complexes to cross the cell membrane is related to their lipophilicity. This is consistent with the premise that investigated Ir compounds likely penetrate the cytoplasmic membrane via passive diffusion.

The ability of antitumor active substances to localize in subcellular structures is their important property from the point of view of understanding their mechanism of biological action. Additionally, it has been shown^40−44^ that several structurally similar antitumor Ir(III) complexes are preferentially localized in the mitochondria of cancer cells. Further experiments performed were therefore aimed at finding out whether the tested Ir complexes after accumulation in MDA-MB-231 cells are localized in their mitochondria. As indicated in Figure S89, the phosphorescence signal from the investigated Ir complexes was not observed in the cell nucleus, but most of the signal was localized in the cytoplasm. It is therefore reasonable to assume that DNA in the cell nucleus cannot be considered the main pharmacological target of the investigated Ir complexes, as is the case, for example, with conventional platinum cytostatics. To determine the organelle in the tumor cell that could be considered as the target site of the antitumor action of the investigated Ir complexes, we performed colocalization experiments in which we compared the phosphorescence yielded by complexes **1**–**9** with that of mitochondria stained with MitoTracker. As indicated in Figure S89, the signal from **Ir1**–**Ir9** corresponded to the fluorescence signal provided by a red dye MitoTracker that selectively accumulates in mitochondria. The results of this experiment were further evaluated using a parametric correlation test. The Pearson correlation coefficient values found for **Ir1**–**Ir9** were 0.37–0.75, confirming for most investigated Ir(III) complexes colocalization with mitochondria and preferential accumulation of these complexes in this organelle of MDA-MB-231 cells; a high level of this colocalization was observed for complexes **Ir1**–**Ir3** and **Ir6**–**Ir8**.

### Mechanism of Cell Death

**Figure 3:**
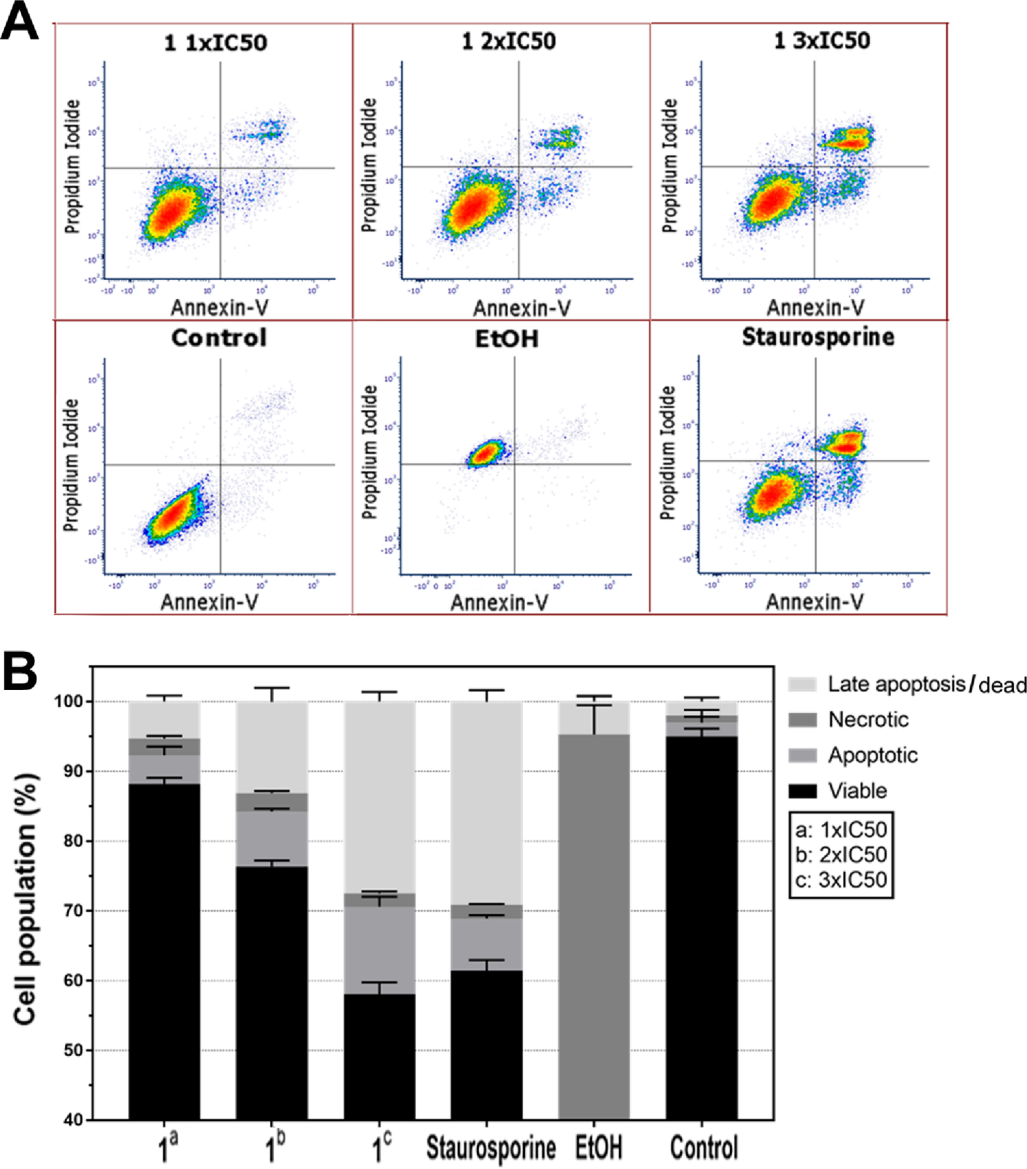
(A) Representative density plot indicating cell death determined by annexin V/PI assay. MDA-MB-231 cells were treated with an equitoxic concentration of **1** corresponding to 1-, 2-, or 3-fold IC_50,72h_. Staurosporine and EtOH represent positive controls for apoptosis and necrosis, respectively. (B) Quantitative analysis of MDA-MB-231 cell death induced by **1** at the indicated concentrations determined using annexin V/PI assay flow cytometry. Error bars are the SDs from three independent experiments.

To further explore whether the investigated Ir complexes belong to cytotoxic or cytostatic drugs, we examined whether the treatment of MDA-MB-231 cells with **1** induces cell death.^45^ We used annexin V/propidium iodide (PI) assay to quantify apoptosis and necrosis in MDA-MB-231 cells treated with **1** for 18 h (the concentration of **1** corresponded to its 1-, 2-, and 3-fold IC_50,72h_ values; Table 1). In addition, staurosporine- and ethanol-treated samples were added as respective apoptotic and necrotic inducer controls. As shown in Figure 3, the annexin V-positive/PI-negative cell population (right bottom quadrant in Figure 3A) increased as a consequence of treating MDA-MB-231 cells with **1**, indicating that **1** induced early apoptosis in these TNBC cells. In addition, treatment with **1** did not result in the formation of early necrotic cells (see the left upper quadrant in Figure 3A demonstrating no annexin V-negative/PI-positive cells). Our data (right upper quadrant in Figure 3A) also indicate that treating these TNBC cells with **1** led to changes in the population of the cells in the late period of apoptosis and dead cells. Qualitatively identical results were obtained if the cells were treated with **Ir2**–**Ir9** (Figure S90). Collectively, these findings demonstrate that the Ir complexes investigated in this study prevent MDA-MB-231 cancer cells from growing or multiplying and kill these TNBC cells in particular by a process of programmed cell death, such as apoptosis.

### Disruption of Cell Cycle Progression

Next, to gain insight into whether the investigated Ir(III) complexes can affect the cell cycle, we examined by flow cytometry the ability of the investigated Ir complexes to impair the cell cycle in MDA-MB-231 cells. After treatment, all compounds **Ir1**–**Ir9** caused, when compared to control cells, an increased population of cells at the G2/M phase, slight depletion of G0/G1-phase cells, and minor change in the population of S-phase cells (Figure S91). Thus, the observed G2 abrogation may force cancer cells into the M phase and the so-called “mitotic catastrophe” and apoptosis.

### Anti-Invasion Activity Determined by Real-Time Monitoring of the Cell Growth

One of the main processes during which cancer cells actively spread from primary tumors is invasion, which is the mechanism by which cancer cells expand and penetrate adjacent tissues. A closely related process is metastasis, i.e., the process involving the spread of tumor cells to a new distant tissue in the body, where a new or secondary tumor is formed. Thus, the acquisition of invasive behavior is an early step in metastasis.^46^

It has been shown that MDA-MB-231 cells display higher metastatic potential than the MCF-7 cells in mouse xenografts.^47^ For this reason, MDA-MB-231 cells were used as the model system for further studies focused on the anti-invasive and antimetastatic properties of the investigated Ir complexes.

**Figure 4:**
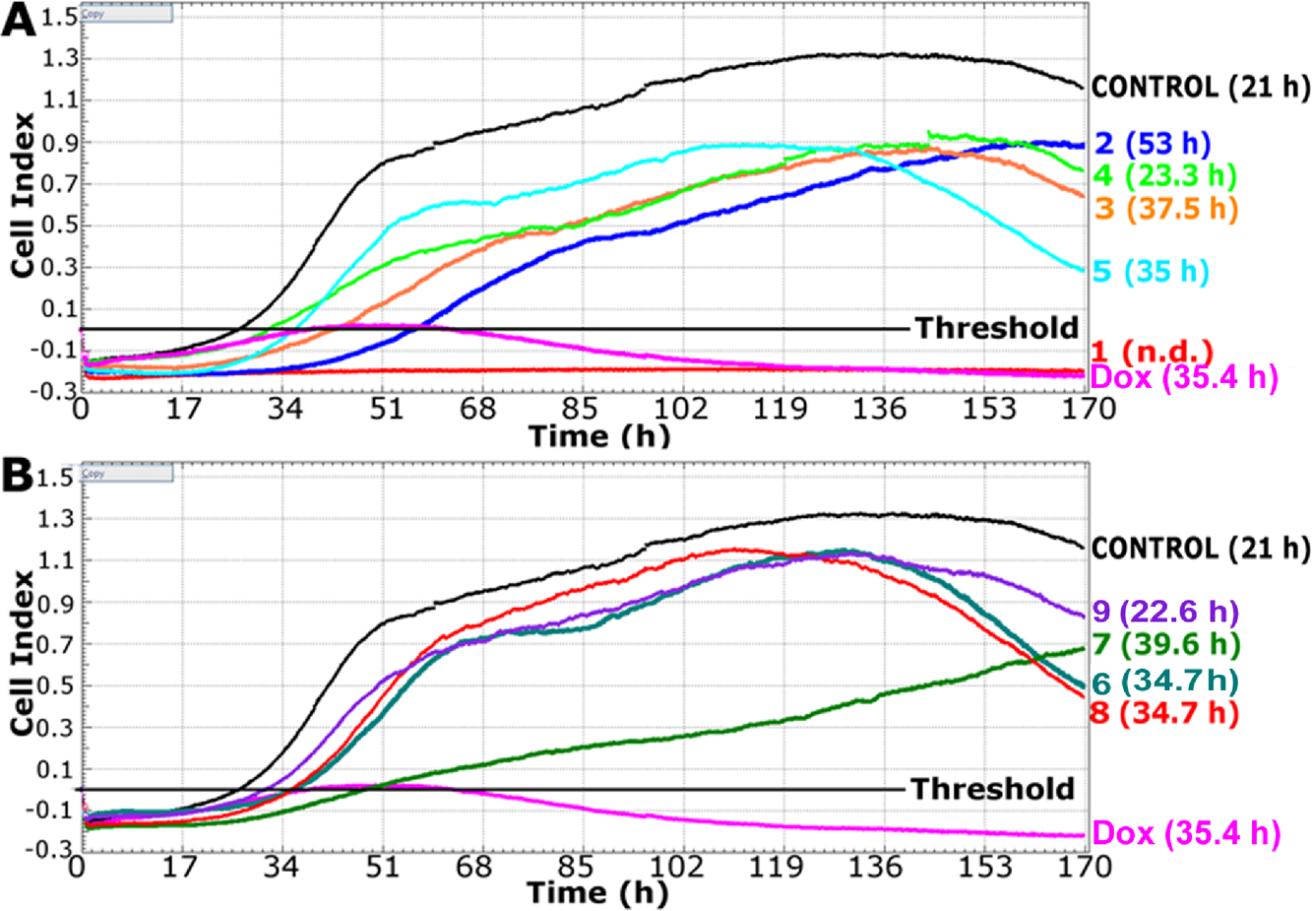
Effects of the investigated Ir complexes and doxorubicin on MDA-MB-231 cell invasiveness in a real-time model. Real-time cell invasion was continuously determined by the real-time cell analyzer (RTCA) xCelligence. Cells were treated with the equitoxic concentrations (IC_50,72h_) of **1**–**5** (A) or **6**–**9** (B) for 24 h before seeding for invasion assay. The time in the brackets indicates the period needed to invade treated cells through the matrix membrane (CI intersects the threshold, *t*_inv_ value); *t*_inv_ values represent the mean from three independent experiments and that each sample was treated in duplicate. Samples were monitored for 7 days.

The anti-invasive effects of the investigated Ir complexes in real time were assessed by the xCELLigence DP instrument with the migration RTCA CIM plate 16.^48^ This system is based on the principle of the Boyden chamber that allows real-time monitoring of the number of invading cells. When cells migrate from the upper to lower wells through the microporous membrane with integrated microelectrodes, they contact and adhere to the microelectrodes affecting the impedance. The output of the experiment performed with this system is the dimensionless cell index (CI); when the cells invade, the CI increases. Moreover, it is possible to analyze the time from cell seeding to the time when invasion through the membrane matrix for each sample treated with tested compounds started (*t*_inv_). This period is determined by the intersection of the curve corresponding to the particular time-dependent cell response profile (TCRP) with the straight line corresponding to the threshold value (CI = 0). The increasing values of *t*_inv_ correspond to the reduced invasiveness of MDA-MB-231 cells treated with the test compounds (Figure 4). The invasion process was monitored for 7 days to determine whether cell death occurred during the subsequent time after the invasion was observed.

It is also important to note that we determined the toxicity of the investigated compounds in the concentrations used in the experiments to assess their anti-invasive effects. We used the Trypan Blue Exclusion Test of Cell Viability for these analyses, which allows for determining the number of viable cells in a cell suspension. This analysis showed that the concentrations of the investigated compounds used in the anti-invasive and antimetastatic assays affected cell viability negligibly (cell viability decreased by only 3–12%). Thus, the data prove that the concentrations of the tested compounds used in the anti-invasive and antimetastatic assays were subtoxic and showed a negligible effect on the viability of treated cells. It should also be noted that for the trypan blue viability assays, we mimicked the experimental design used for all anti-invasive and antimetastatic assays, including cell seeding densities, the format of culture plastics used, etc.

The data in Figure 4 indicate a complete inhibition of MDA-MB-231 cell invasion after the treatment with compound **Ir1** (Figure 4A), indicating a complete abrogation of the invasiveness of this cell line exhibiting high metastatic potential. It is worth noting that the control compound, doxorubicin, often used to treat TNBC, yielded a different TCRP, showing that the value of *t*_inv_ was approximately 35 h. This result suggests a distinctly higher efficiency of compound **Ir1** to reduce the invasiveness of MDA-MB-231 in comparison with doxorubicin. The values of *t*_inv_ obtained for the samples of MDA-MB-231 cells treated with other investigated Ir complexes indicated that the efficiency of compound **Ir2** in reducing the invasiveness of MDA-MB-231 cells (*t*_inv_ = 53 h) was also higher than that of doxorubicin. In contrast, the efficiency of compounds **Ir3**, **Ir5**, **Ir7**, and **Ir8** (*t*_inv_ = 35–40 h) was similar to that of doxorubicin. On the other hand, compounds **Ir4** and **Ir9** slowed down the process of an invasion only negligibly (*t*_inv_ = 23 h) compared to the control, untreated samples (*t*_inv_ = 21 h).

TCRPs obtained for some of the investigated compounds (compounds **Ir3**–**Ir6**, **Ir8**, **Ir9**, and doxorubicin) and the untreated control showed a gradual decrease in CI after an initial increase. This observation can mean that cell death was triggered in invading cell subpopulations after more extended treatment periods.

### Flow Cytometric Quantification of Vimentin

The ability of the tested agents to act antimetastatically on tumor cells is often evaluated based on the ability of these agents to downregulate the expression of vimentin in tumor cells. Vimentin is a type III intermediate filament protein highly expressed in aggressive epithelial cancers, inducing tumor cell migration, and is thus associated with increased metastasis rates. Vimentin is therefore an important marker for epithelial–mesenchymal transition (EMT), i.e., the process employed by cancer cells to acquire the ability to invade the surrounding tissues, resist therapeutic agents, and escape immunity.

Vimentin has well-established roles in cell migration and motility. Furthermore, many studies identify vimentin as a new targetable protein for effectively eliminating drug resistance and recurrence potency in the case of TNBC.^49,50^ Therefore, we wanted to determine whether the expression of vimentin could be affected by the investigated Ir complexes directly in TNBC MDA-MB-231 VIM RFP cells with stable expression of RFP (red fluorescent protein) reporter associated with vimentin.

**Figure 5:**
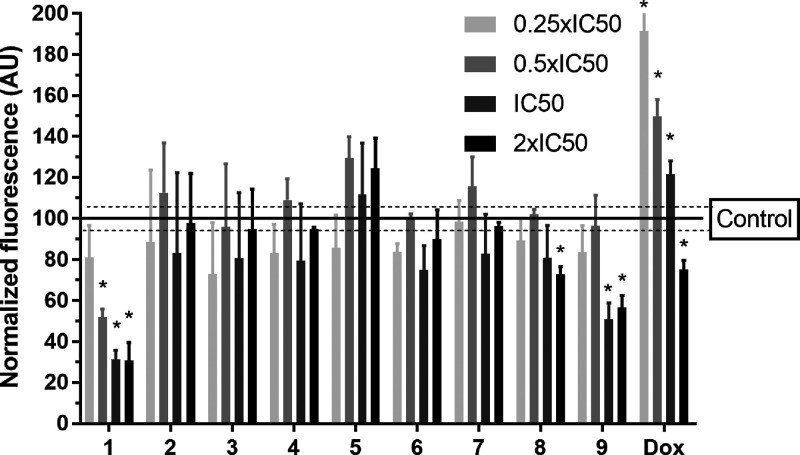
Flow cytometric analysis of MDA-MB-231 VIM RFP cells treated with the investigated compounds. Cells were treated for 72 h with increasing concentrations of the compounds and analyzed by flow cytometry to determine the fluorescence of the VIM RFP reporter. Values of the fluorescence were normalized to the untreated control. Data are the means from four independent experiments; error bars define the SDs, and stars (*) at the top of the bars denote a significant difference from untreated control samples (*P* ≤ 0.01) calculated by using Student’s *t*-test.

Expression of vimentin in MDA-MB-231 VIM RFP cells was analyzed by flow cytometry after 24, 48 (data not shown), and 72 h treatment with the investigated compounds used at the equitoxic concentrations corresponding to 0.25-, 0.5-, 1-, and 2-fold IC_50,72h_ (Figure 5). After 24 or 48 h, the treatment with the investigated compounds resulted in only insignificant alterations in vimentin expression (not shown). However, after the 72 h treatment, significantly opposite effects were observed after MDA-MB-231 VIM RPF cells were treated with **Ir1** and conventional doxorubicin. Only so high concentrations of doxorubicin as those corresponding to 2-fold higher IC_50,72h_ obtained for this drug somewhat suppressed the RFP fluorescence in MDA-MB-231 VIM RFP cells. In contrast, sub-toxic concentrations of doxorubicin considerably increased vimentin expression in the cells, consistent with the ability of doxorubicin to enhance breast cancer cell migration and invasion.^16,51^

On the other hand, **Ir1** significantly reduced the RFP fluorescence in MDA-MB-231 VIM RFP cells at its concentrations corresponding to half to twice the IC_50,72h_ value. Interestingly, the treatment with **Ir9** also downregulated the vimentin expression but was markedly less efficient than **Ir1**. Taken together, our findings confirm that treatment of TNBCs with conventional doxorubicin enhances their invasiveness and ability to migrate,^52^ which is one of the typical dose-dependent side effects of doxorubicin treatment.^53^ To more thoroughly examine mechanisms underlying antimetastatic effects of the investigated Ir complexes in TNBC cells, we analyzed the expression of vimentin and other typical EMT markers in more detail by reverse transcription quantitative PCR (RT-qPCR).

### Expression of Genes Related to EMT and MET

Information about the antimetastatic efficiency of the investigated Ir complexes can also be evaluated based on their ability to affect the expression of genes related to epithelial-to-mesenchymal transition (EMT) and its reverse mesenchymal-to-epithelial transition (MET) in tumor cells. It is so because EMT and MET have been suggested to play crucial roles in the metastatic dissemination of tumors.

For instance, the process of EMT is accompanied by the downregulation of expression of the epithelial cell marker E-cadherin and upregulation of expression of mesenchymal markers like vimentin, N-cadherin, β-catenin, and the transcription factor Snail1. In other words, the upregulation of expression of E-cadherin and downregulation of expression of vimentin, N-cadherin, β-catenin, and Snail1 induced by the investigated compound would indicate its antimetastatic activity.

**Figure 6:**
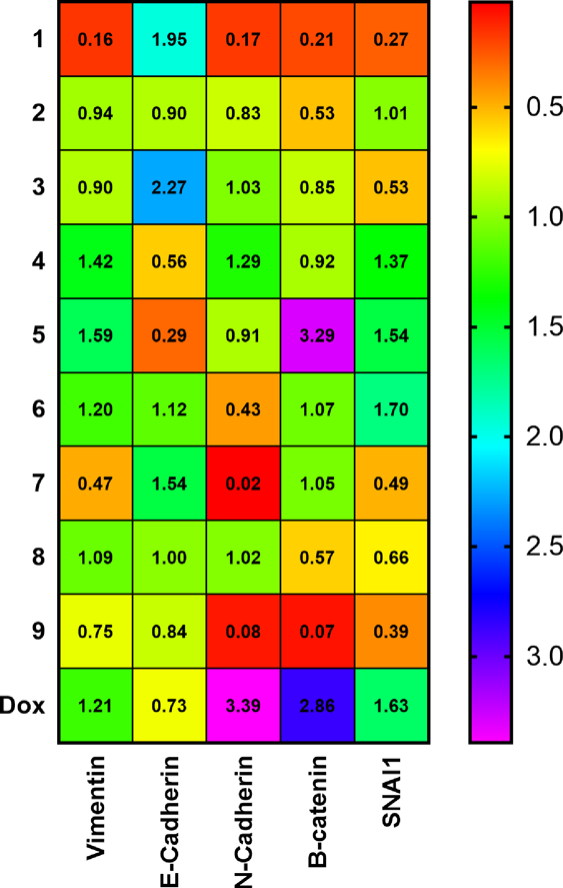
Relative mRNA expression of the EMT-related genes in MDA-MB-231 cells. Cells were treated for 24 h with the equitoxic concentration of tested compounds (IC_50,72h_). Relative quantification of the respective mRNA gene expression was calculated using the 2^–ΔΔ*C*t^ method. β-Actin was used as the endogenous reference control, and the untreated control samples were used for arbitrary calibration. Data were depicted as the heat map. The data are the means from four independent experiments, with a quadruplicate of each gene analyzed in the respective PCR run.

To determine the expression levels of various EMT- or MET-related genes, we performed the RT-qPCR analysis in MDA-MB-231 cells treated with the investigated Ir complexes and, for comparative purposes, also conventional doxorubicin. The cells were treated for 24 h with the equitoxic concentration of the investigated compounds (IC_50,72h_), and the analysis of expression of the EMT- or MET-related gene through messenger RNA (mRNA) quantitation was performed using the 2^–ΔΔ*C*t^ method (Figure 6).

The results show that some Ir complexes tested, particularly complex **Ir1**, met the above criteria for antimetastatic agents. The treatment of MDA-MB-231 cells with complex **Ir1** downregulated the expression of vimentin (∼6-fold), N-cadherin (∼6-fold), β-catenin (∼5-fold), and Snail1 (∼4-fold) and upregulated the expression of E-cadherin. These observations highlight the potential of **Ir1** to act as an efficient antimetastatic agent.

Next, we sought to gain more insight into the ability of doxorubicin, a widely used drug used to cure breast carcinoma (including its triple-negative sub-variant), to affect the expression of EMT-related genes. The treatment of MDA-MB-231 cells with doxorubicin upregulated the expression of N-cadherin (∼3-fold), β-catenin (∼3-fold), and SNAI1 (∼2-fold) and slightly upregulated also the expression of vimentin; in addition, the expression of E-cadherin slightly decreased. These results are consistent and support the view that sub-lethal doses of doxorubicin activate the EMT pathway in breast cancer cells.^52−56^ Thus, these findings confirm that anticancer chemotherapy using doxorubicin is inappropriate as it can cause drug-resistant TNBC cells to metastasize,^16^ so the search for new drugs to treat breast cancer that would show better antitumor effects than doxorubicin is justified.

The results described so far show that of the iridium complexes studied, especially **Ir1** exhibits the properties of an agent capable of acting antimetastatically on TNBCs. In contrast, these results also show that treatment of TNBC cells with doxorubicin used in conventional chemotherapy of TNBC enhances their invasiveness and ability to migrate. Therefore, next, we thought to gain more insight into the mechanism of the antimetastatic action of **Ir1** and, conversely, the promigratory activity of doxorubicin.

### Morphology of the Vimentin Intermediate Filament Network

Interestingly, there is an accumulation of evidence in the literature demonstrating that overexpression of vimentin and the formation of vimentin intermediate filaments (VIFs) in cancer cells lead to an augmentation of their motility, invasiveness, and metastasis formation.^57,58^ Additionally, VIFs are known to be critical for regulating cell shape and, consequently, migration.^59^ Thus, due to the assembly of vimentin filaments, cancer cells adopt the elongated shape typical for mesenchymal cells, whereas the cells that do not express VIFs approximate circular shapes.^60^

**Figure 7:**
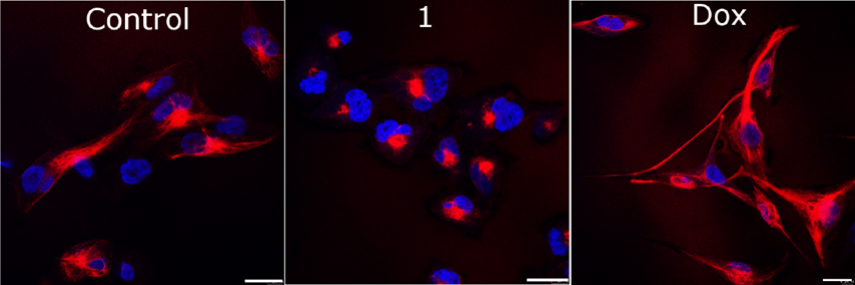
Confocal microphotograph of MDA-MB-231 VIM RFP cells. Cells were treated for 24 h with the equitoxic concentration of tested compounds corresponding to IC_50,72h_. Vimentin filaments were tagged with RFP (red channel), whereas cell nuclei were counterstained with DAPI (blue channel). Figures are the representatives of three independent experiments. Scale bars represent 20 μm.

We investigated the morphology of VIFs in MDA-MB-231 VIM RFP cells treated with **Ir1** or doxorubicin by fluorescence imaging using confocal microscopy (Figure 7). Control (untreated) cells displayed an apparent trailing type of vimentin polarization.^61^ On the other hand, most of VIFs in MDA-MB-231 VIM RFP cells treated with doxorubicin adopted a typical structure,^57,58^ supporting the view that doxorubicin induces overexpression of VIFs in TNBCs, promoting their motility and invasiveness. Conversely, the cells treated with **Ir1** exhibited significant depolymerization and condensation of the vimentin cytoskeletal network, i.e., an epithelial-like morphology. Taken together, the morphological analysis of VIFs in MDA-MB-231 VIM RFP cells treated with **Ir1** or doxorubicin revealed an undisputable ability of **Ir1** to act in TNBC cells as the antimetastatic agent. Contrary to this, treating MDA-MB-231 VIM RFP cells with doxorubicin stimulates TNBC cells to the pro-invasive character.

### Wound Healing Assay

**Figure 8:**
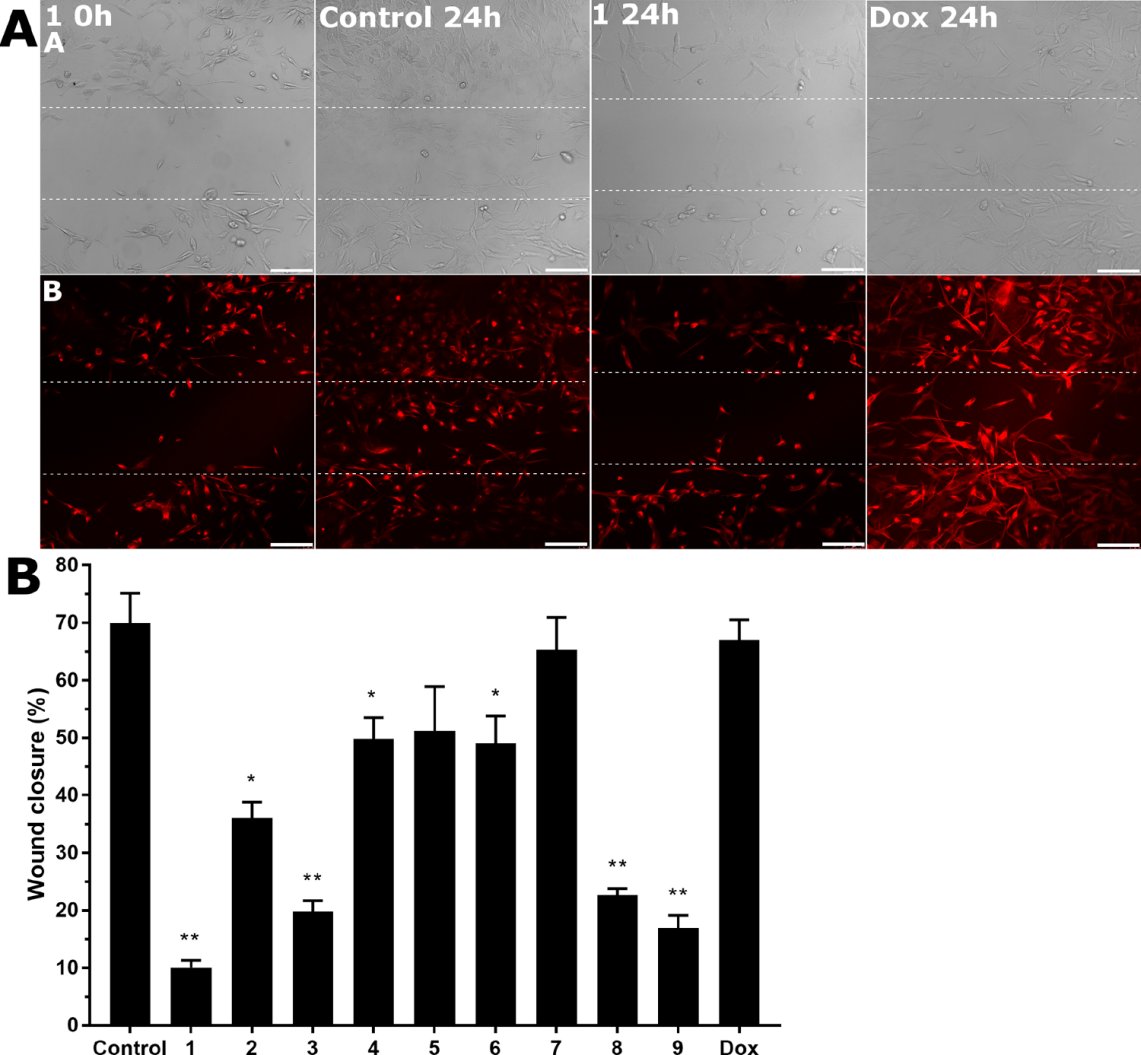
Migration of MDA-MB-231 VIM RFP cells by *in vitro* wound healing assay and confocal microscopy. MDA-MB-231 VIM RFP cells were treated for 24 h with an equitoxic concentration of **Ir1** or doxorubicin corresponding to their IC_50,72h_ values. (A) Images from a wound healing assay experiment were acquired at 0 and 24 h after treatment. Row A, bright field; row B, fluorescence from VIM RFP. The dashed horizontal straight lines mark scratch boundaries; scale bars represent 100 μm. (B) Analysis of the wound healing assay. Images were analyzed by ImageJ software, and both channels (bright field and fluorescence) were taken for further analysis. The wound closure area was calculated. Data were submitted to statistical analysis using Student’s *t*-test, and the stars at the top of the bars denote a significant difference from the untreated control with **P ≤ 0.001 and *P ≤ 0.05.

Scratch or wound healing assay is a straightforward method for probing cell migration in two dimensions toward the newly created free space to close the wound and establish new cell–cell contacts. This method represents the raw approximation of how cancer cells can spread.^62^ We used MDA-MB-231 VIM RFP cells and confocal microscopy to monitor wound closure more precisely and distinguish tiny pseudopods of migrating cells. Thus, we could monitor the expanding capacity of the cells treated with the investigated compounds (Figure 8A) and compare the wound closure capacity with that of the control (untreated) cells. The cells were treated with the equitoxic concentration of the compounds corresponding to the IC_50,72h_ values, and the wound closure capacity was evaluated 24 h after the treatment (Figure 8B). The wound closure capacity of **Ir1** was only 10%, demonstrating its strong antimigratory effects in MDA-MB-231 VIM RFP cells. Contrastingly, the wound closure capacity of conventional doxorubicin (67%) was very similar to that displayed by the control, untreated cells (70%). Additionally, the treatment of MDA-MB-231 VIM RFP cells with doxorubicin induced formation of the pseudopods visualized with the aid of the tagged vimentin (Figure 8A), indicating the efficient colonization of the wound area by cancer cells. Thus, these results suggest that doxorubicin at the concentration corresponding to IC_50,72h_ stimulates the TNBC cells to migratory and motility movements and can hardly be recommended for treatment of the highly invasive TNBC. In contrast, the result of the wound healing experiment confirmed the strong antimigratory effects of **Ir1** in TNBC cells, suggesting its considerable potential to inhibit the invasiveness of TNBC cells.

### Morphological Quantification of Tumor Spheroids and Quantification of Cell-Invasive Potential

The cancer cell transition from proliferation to invasion is usually initiated by nutrient stress and unfavorable conditions caused by the progressive growth of the primary tumor.^63^ To study the dynamics of cells to evade the primary tumor, we monitored the morphology of spheroids generated from MDA-MB-231 cells embedded in an agar matrix.^64^ Multicellular tumor spheroids represent the most used 3D *in vitro* model^65^ and offer the opportunity to study the invasive potential of cancer cells under conditions much closer to the *in vivo* situation than when the traditional Transwell assay is used.^64^ Morphological parameters were utilized to evaluate the invasive potential of spheroids generated from MDA-MB-231 cells.^66^ Spheroid perimeter is the most useful morphological parameter for quantifying overall proliferation, whereas roundness, shape integrity, and quantification of protrusion occurrence are the morphological parameters related to the invasion potential.^64,66^ At the most compact stage, cells on the surface follow the contour of the spheroid. This morphology resembles the proliferating but not invading character of the spheroid, whereas diffuse and rough borders are the signs of the invasive character of peripheral cells. Assessment of the spheroid circularity (roundness) was analyzed by the automated edge detection and measurement of the enclosed area. A circularity equal to one indicates a perfectly circular spheroid, whereas lower values indicate a loss of circularity. The solidity is an indicator of the regularity of the spheroids’ surface or its roughness and thus can be considered the indicator of the primary protrusions and the overall regularity of the spheroid.^66^

**Figure 9:**
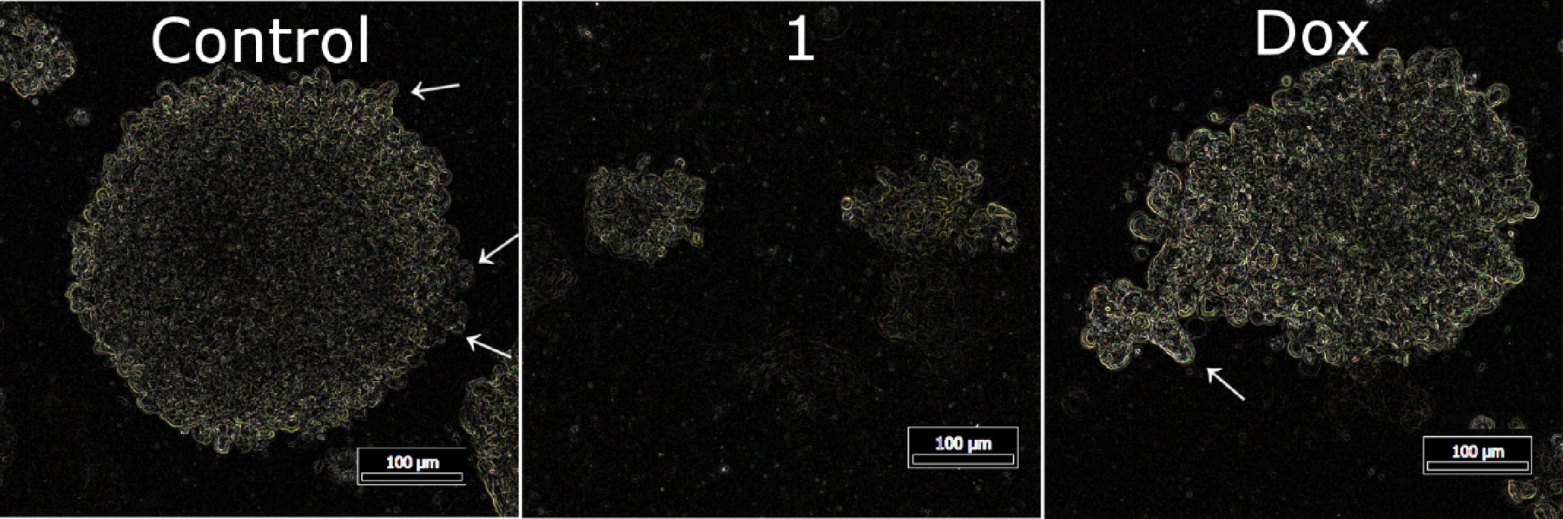
Morphological quantification of the invasive potential of MDA-MB-231 cells invading from the 3D tumor spheroids. One week-old spheroids were treated for 72 h with tested compounds at the concentration corresponding to IC_50,72h_ and analyzed by ImageJ software. The scale bar represents 100 μm (arrows, protrusions).

MDA-MB-231 cells were seeded to the agar matrix and cultured for 7 days to form regular mammospheres. Then, the spheroids were treated with the equitoxic concentrations of **Ir1** or doxorubicin corresponding to their IC_50,72h_ values. After 72 h of treatment, spheroids were photographed by a phase contrast microscope and subjected to morphological analysis with ImageJ software (Figure 9 and Table 3). The detailed morphological analysis of control (untreated) spheroids shows a mean perimeter of 2.1 mm, an almost perfect circularity of 0.96, and a concave character with a dark spheroid center indicating high compactness. On the other hand, the increased roughness of the untreated spheroids, along with a high occurrence of primary and secondary protrusions, is an indication of metastatic potential of MDA-MB-231 spheroids. Furthermore, secondary protrusions were identified in the samples treated with doxorubicin. These protrusions were up to 73 μm in length and belonged to the main characteristics of the high invasiveness of the peripherical cells. The high solidity (6.3) of spheroids treated with doxorubicin also indicates a high roughness of spheroids and, thus, an invasive potential of the cell structures on the periphery of spheroids. Notably, spheroids treated with doxorubicin also displayed a high perimeter of about 2.8 mm. In contrast, spheroids treated with **Ir1** display a low mean perimeter of 0.8 mm without the occurrence of protrusions. In addition, their structure collapsed, which resulted in a significant false positive increase in the solidity factor (Table 3). Spheroids treated with compound **Ir1** and doxorubicin also showed very loose compactness, which could be identified by the bright spheroid core. Contrary, spheroids (untreated controls) showed a dark core, indicating a compact character of spheroids. The tiny pseudopods were not captured mainly due to the low magnification used to capture the entire spheroid; thus, only large cellular structures evading the spheroid border could be determined.

**Table 3:** Morphological Analysis of 3D Spheroids Generated from MDA-MB-231 Cells^a^

	perimeter (μm)	circularity	solidity	protrusion occurrence (number: length); secondary protrusions (SP^b^)
control	2120 ± 310	0.96	3.0	4: 32 μm
**Ir1**	800 ± 111	0.82	4.7^c^	ND
Dox	2820 ± 506	0.66	6.3	2: 73 μm; SP

a Experiments were performed in triplicate, with the subsequent analysis of 10 spheroids per sample.

b SP, secondary protrusions or protrusion-related domains.

c The solidity was affected by the collapse of the spheroid structure and complete disaggregation.

## Conclusions

We have synthesized nine new anticancer cyclometalated Ir(III) agents of the type [Ir(ttpy)(C^N)Cl]PF_6_**Ir1**–**Ir9** to explore the effect of the modifications carried out within the 2-arylbenzimidazole C^N scaffold on their optical properties and their biological activity. In addition, an ester functionality was installed as a handle for further intended functionalization in **Ir9.** Stability tests of the coordination complexes in DMSO and cell culture medium were performed using UV/Vis spectroscopy, showing no tendency toward a decrease in absorption with time. The structural modifications within the C^N scaffold strongly impacted the biological properties. The antiproliferative activity of the investigated Ir(III) complexes was determined against the triple-negative breast cancer (TNBC) cells and a non-malignant breast MCF-10A cell line derived from the same epithelial origin. The results show that two of the investigated Ir complexes (**Ir1** and **Ir4**) exhibit in TNBC cells a potency similar to that found for doxorubicin used as a conventional drug to treat TNBC but higher selectivity for TNBC cells versus non-cancerous cells compared to doxorubicin (Table 1). Currently, antimetastatic targeted chemotherapy represents one of the most important cancer treatments, especially in tumors with high metastatic potential. The highly aggressive TNBC cell line MDA-MB-231 belongs to tumor cell lines with high metastatic potential. Therefore, our subsequent studies focused on evaluating the antimetastatic effectiveness of the investigated Ir(III) complexes in highly aggressive TNBC MDA-MB-231 cells. The anti-invasive effects of the investigated Ir complexes in real time were assessed using the real-time cell analyzer (RTCA) xCelligence. The results (Figure 4) showed that of the studied Ir complexes, especially **Ir1** exhibited distinctly higher anti-invasive activity in MDA-MB-231 cells than conventional doxorubicin, which is known to exhibit a proinvasive effect.^16,51,52^

To obtain further information about the ability of the studied Ir complexes to suppress the metastatic spreading of TNBC cells, we analyzed the expression of a type III intermediate filament protein vimentin in MDA-MB-231 VIM RFP cells by flow cytometry. Vimentin is highly expressed in aggressive epithelial cancers, inducing tumor cell migration, and is thus associated with increased metastasis rates. Our data (Figure 6) revealed that in particular, **Ir1** markedly downregulated the vimentin expression, whereas already sub-toxic concentrations of conventional doxorubicin considerably increased vimentin expression in the cells, consistent with the ability of doxorubicin to enhance breast cancer cell migration and invasion. The potential of **Ir1** to act as an efficient antimetastatic agent was further highlighted by its efficiency in affecting the expression of genes related to EMT and MET in TNBC MDA-MB-231 cells. **Ir1** effectively upregulated the expression of the genes associated with MET and downregulated the expression of the gene related to EMT (Figure 6). Contrastingly, the treatment of MDA-MB-231 cells with doxorubicin downregulated the expression of the genes associated with MET and upregulated the expression of the gene related to EMT, confirming that treatment of TNBC cells with doxorubicin used in conventional chemotherapy of TNBC enhances their invasiveness and ability to migrate. To further study the antimetastatic properties of Ir terdentate complexes, the morphology of VIFs in MDA-MB-231 VIM RFP cells treated with the investigated compounds was examined by fluorescence imaging using confocal microscopy (Figure 7). Indeed, the treatment of MDA-MB-231 VIM RFP cells with **Ir1** resulted in the epithelial-like morphology, confirming the ability of **Ir1** to act in TNBC cells as the antimetastatic agent. Expectably, the treatment with conventional doxorubicin led to the elongated shape typical for mesenchymal cells, supporting the view that doxorubicin induces overexpression of VIFs in TNBCs, promoting their motility and invasiveness. To further validate the results obtained from the previous assays, the wound healing assay was conducted as well (Figure 8). The results of this experiment confirmed the strong antimigratory effects of **Ir1** in TNBC cells, suggesting its considerable potential to inhibit the invasiveness of TNBC cells. On the other hand, these results demonstrated that doxorubicin rather stimulated the migratory and motility movements of TNBC cells so that it can hardly be recommended for treatment of the highly invasive TNBC.

We also verified the antimetastatic potential of **Ir1** and, conversely, the metastatic effects of conventional doxorubicin under conditions much closer to the *in vivo* situation. To perform this analysis, we monitored the morphology of 3D spheroids generated from MDA-MB-231 cells embedded in an agar matrix (Figure 9). The morphology of the cells treated with **Ir1** showed features typical of non-invasive cells, whereas the tumor sphere morphology after the treatment with doxorubicin revealed primary and secondary protrusions, representing the invading cells from the spheroid surface.

In summary, the results of this work testing antiproliferative and antimetastatic effects of the Ir(III) compounds containing a terdentate ligand by running several assays suggest that these Ir compounds may hold promise as a platform to improve the treatment of TNBC by inhibiting their antiproliferative and metastatic properties.

## Experimental Section

### Reagents, Chemicals, Cell Lines, and Culture Conditions

4′-(*p*-Tolyl)-2,2′:6′,2″-terpyridine, 4-(trifluoromethyl)benzyl bromide, iodomethane, 2-phenylbenzimidazole, 4-chloro-3-nitrobenzoic acid, butylamine, zinc in powder, ammonium formate, 3,4-(methylenedioxy)benzaldehyde, 2-naphthaldehyde, 1,2-phenylenediamine, 2-thiophenecarboxaldehyde, triethylamine, trifluoroacetic acid, magnesium sulfate, potassium hexafluorophosphate, dimethyl sulfoxide (DMSO), and ethylene glycol were obtained from Sigma-Aldrich (Madrid, Spain). IrCl_3_ was obtained from Johnson Matthey. Deuterated solvents were obtained from Euriso-top.

MDA-MB-231, MDA-MB-231 VIM RFP, MCF-7, and MCF10A were from ATCC. Cells were maintained in Dulbecco’s minimum essential medium (Biosera) supplemented with 10% FBS (Biosera) and gentamicin (Merck). Cells were cultured in a humidified CO_2_ (5%) incubator and subcultured 2–3 times per week according to the proliferation of each cell line, but still at the sub-confluent state, to maintain exponential growth characteristics. Sulforhodamine B, doxorubicin, and propidium iodide were from Merck (Germany). The Geltrex matrix was from Gibco. All the chemicals were in a purity suitable for cell culture conditions. The purity ≥95% of the synthesized complexes used for biological evaluation was determined by NMR and RP-HPLC.

### Synthesis Procedures

[Ir(ttpy)Cl_3_] was prepared based on the procedure reported by Porras et al.^67^ The terdentate ligand 4′-(*p*-tolyl)-2,2′:6′,2″-terpyridine was commercially available.

#### Synthesis of HC^N Proligands (**HL1**–**HL8**)

The preparation of proligands **HL1**–**HL8** was carried out with slight modifications of the literature method.^32,33^ In the first step, the corresponding aldehyde (1 mmol) and sodium bisulfide (10 mmol) were stirred in water at 100 °C for 1 h. Then, *o*-phenylenediamine (1 mmol) was dissolved in EtOH and added to the reaction mixture that was heated overnight at 80 °C. The corresponding 2-(aryl)benzimidazole **A1**–**A8** (Scheme 2) were filtered and washed with water and hexane. In the second step, the corresponding 2-(aryl)benzimidazole (0.5 mmol) and 4-(trifluoromethyl)benzyl bromide or iodomethane (0.6 mmol) and Cs_2_CO_3_ (1 mmol) were stirred in acetonitrile at 45 °C for 24 h. The solvent was removed under reduced pressure, and dichloromethane was added and extracted with water (3 × 30 mL). The organic phase was dried, and a pure white solid was obtained.

##### HL4

White solid. Isolated yield: 43%. ^1^H NMR (400 MHz, CDCl_3_) δ 7.86 (dd, *J* = 8.0, 1.0 Hz, 1H, H_b_), 7.60 (d, *J* = 8.1 Hz, 2H, H_l_), 7.32 (tt, *J* = 7.2, 1.1 Hz, 1H, H_c_), 7.23 (ddd, *J* = 8.0, 7.1, 0.9 Hz, 1H, H_d_), 7.21 (d, *J* = 8.1 Hz, 2H, H_k_), 7.16 (d, *J* = 1.7 Hz, 1H, H_o_), 7.13 (d, *J* = 8.3 Hz, 1H, H_e_), 7.09 (dd, *J* = 8.0, 1.7 Hz, 1H, H_s_), 6.86 (dd, *J* = 8.0, 0.6 Hz, 1H, H_r_), 6.03 (s, 2H, H_t_), 5.50 (s, 2H, H_i_). ^13^C{^1^H} NMR (151 MHz, CDCl_3_) δ 153.9 (C_g_), 149.4 (C_q_), 148.3 (C_p_), 143.2 (C_a_), 140.5 (C_j_), 135.9 (C_f_), 130.4 (C_n_), 126.4 (C_k_), 126.3 (C_l_), 125.0 (C_m_), 123.6 (C_h_), 123.5 (C_s_), 123.3 (C_d_), 123.1 (C_c_), 120.2 (C_b_), 110.2 (C_e_), 109.7 (C_o_), 108.8 (C_r_), 101.7 (C_t_), 48.1 (C_i_). Mass ESI-MS (pos. ion mode, DMSO): calc.: [M + H]^+^ = 397.1158 *m*/*z*; exp.: 397.1154 *m*/*z*. Anal. calc. for C_22_H_15_F_3_N_2_O_2_: %C, 66.67; %H, 3.81; %N, 7.07. Found: %C, 66.81; %H, 3.80; %N, 7.06.

The synthesis of proligand **HL9** was performed in some different steps. The diamine precursor **B1** was obtained as previously described from the corresponding nitro derivative (Scheme S1).^29^ Also, the condensation between the diamine and the aldehyde followed the same method as **A1**–**A8** (Scheme 2).

#### Synthesis of Ir(III) Complexes (**Ir1**–**Ir9**)

All complexes were synthesized after slightly modifying the reported method.^68^ Complex [Ir(ttpy)Cl_3_] (0.1 mmol), potassium hexafluorophosphate (0.6 mmol), the corresponding HC^N proligand (0.2 mmol), and 4 mL of ethylene glycol were added to the microwave vial and were heated for 12 min at 240 °C. The reaction mixture was cooled to room temperature, and an orange precipitate was observed. It was filtered and washed with water and diethyl ether. The resulting solid was solved in acetonitrile and passed through an alumina column. The pure orange product was obtained in good yields.

##### Ir1

Orange solid. Isolated yield: 79%. ^1^H NMR (400 MHz, DMSO-*d*_6_) δ 9.25 (s, 2H, H_7_), 8.98 (d, 2H, H_4_), 8.94 (dd, 1H, H_b_), 8.26 (d, 2H, H_10_), 8.22 (d, 2H, H_3_), 7.93 (m, 3H, H_e+1_), 7.75 (d, *J* = 8.2 Hz, 2H, H_l_), 7.61 (t, 2H, H_2_), 7.57–7.42 (m, 7H, H_11+k+q/p+d+c_), 6.16 (s, 2H, H_i_), 6.13 (d, *J* = 4.9 Hz, 1H, H_p/q_), 2.50 (s, 3H, H_13_). ^13^C{^1^H} NMR (101 MHz, DMSO-*d*_6_) δ 159.5 (C_g_), 158.0 (C_5_), 155.8 (C_6_), 152.4 (C_1_), 151.2 (C_8_), 146.4 (C_h_), 141.1 (C_12_), 140.9 (C_a_), 140.4 (C_j_), 139.9 (C_3_), 134.8 (C_f_), 132.6 (C_q/p_), 132.1 (C_9_), 129.7 (C_11_), 128.9 (C_2_), 128.5 (C_p/q_), 128.3, 128.0, 127.9 (C_10_), 126.9 (C_k_), 125.7 (C_l_), 125.6, 125.5 (C_4_), 125.2, 124.1 (C_c_), 123.2 (C_d_), 123.0 (C_o_), 122.5, 120.9 (C_7_), 117.0 (C_b_), 111.2 (C_e_), 47.6 (C_i_), 20.7 (C_13_). ^19^F{^1^H} NMR (377 MHz, DMSO-*d*_6_): δ −63.11 (s, 3F, CF_3_), −72.95 (d, *J*_P-F_ = 714.5 Hz, 6F, PF_6_). Mass ESI-MS (pos. ion mode, DMSO): calc.: [M-PF_6_]^+^ = 908.1414 *m*/*z*; exp.: 908.1434 *m*/*z*. Anal. calc. for C_41_H_29_ClF_9_IrN_5_PS: %C, 46.75; %H, 2.78; %N, 6.65; %S, 3.04. Found: %C, 46.79; %H, 2.84; %N, 6.63; %S, 3.19.

##### Ir2

Orange solid. Isolated yield: 65%. ^1^H NMR (400 MHz, DMSO-*d*_6_) δ 9.26 (s, 2H, H_7_), 9.17 (dd, *J* = 8.0, 0.7 Hz, 1H, H_b_), 8.98 (dd, *J* = 8.0, 0.6 Hz, 2H, H_4_), 8.27 (d, *J* = 8.3 Hz, 2H, H_10_), 8.23 (td, *J* = 8.0, 1.4 Hz, 2H, H_3_), 8.05 (dd, *J* = 8.2, 0.4 Hz, 1H, H_e_), 7.90 (dd, *J* = 5.7, 1.4 Hz, 2H, H_1_), 7.76 (d, *J* = 8.2 Hz, 2H, H_l_), 7.62 (dd, *J* = 7.6, 1.3 Hz, 1H, H_s_), 7.58–7.50 (m, 8H, H_d+c+k+2+11_), 6.81 (td, *J* = 7.6, 1.0 Hz, 1H, H_r_), 6.71 (td, *J* = 7.6, 1.3 Hz, 1H, H_q_), 6.40 (s, 2H, H_i_), 6.13 (dd, *J* = 7.6, 1.0 Hz, 1H, H_p_), 2.50 (s, 3H, H_13_). ^13^C{^1^H} NMR (101 MHz, DMSO-*d*_6_) δ 160.8 (C_g_), 157.9 (C_5_), 155.4 (C_6_), 152.1 (C_1_), 151.0 (C_8_), 142.4 (C_o_), 141.1 (C_12_), 140.7 (C_j_), 140.3 (C_a_), 139.8 (C_3_), 136.1 (C_f_), 133.0 (C_h_), 132.1 (C_9_), 130.7 (C_p_), 130.3 (C_q_), 129.7 (C_11_), 128.8 (C_2_), 128.2, 127.8 (C_10_), 126.7 (C_k_), 125.7 (C_4+l_), 125.5 (C_s_), 125.2, 124.3 (C_c_), 124.2 (C_d_), 123.4 (C_r_), 120.9 (C_7_), 118.2 (C_b_), 111.2 (C_e_), 47.0 (C_i_), 20.7 (C_13_). ^19^F{^1^H} NMR (377 MHz, DMSO-*d*_6_): δ −63.12 (s, 3F, CF_3_), −72.97 (d, *J*_P-F_ = 709.3 Hz, 6F, PF_6_). Mass ESI-MS (pos. ion mode, DMSO): calc.: [M-PF_6_]^+^ = 902.1849 *m*/*z*; exp.: 902.1879 *m*/*z*. Anal. calc. for C_43_H_31_ClF_9_IrN_5_P: %C, 49.31; %H, 2.98; %N, 6.69. Found: %C, 49.57; %H, 2.96; %N, 6.52.

##### Ir3

Orange solid. Isolated yield: 70%. ^1^H NMR (600 MHz, DMSO-*d*_6_) δ 9.30 (s, 2H, H_7_), 9.24 (d, *J* = 8.2 Hz, 1H, H_b_), 9.00 (d, *J* = 7.9 Hz, 2H, H_4_), 8.32 (d, *J* = 8.1 Hz, 2H, H_10_), 8.27 (s, 1H, H_s_), 8.20 (td, *J* = 7.9, 0.8 Hz, 2H, H_3_), 8.15 (d, *J* = 8.4 Hz, 3H, H_e_), 7.94 (d, *J* = 5.1 Hz, 2H, H_1_), 7.77 (d, *J* = 8.2 Hz, 2H, H_l_), 7.65 (m, 4H, H_t+d+k_), 7.58 (d, *J* = 7.9 Hz, 2H, H_11_), 7.55 (d, *J* = 7.2 Hz, 1H, H_c_), 7.52 (t, *J* = 7.9 Hz, 2H, H_2_), 7.32 (d, *J* = 6.9 Hz, 1H, H_v_), 7.24 (m, 2H, H_u+w_), 6.55 (s, 2H, H_i_), 6.47 (s, 1H, H_p_), 2.52 (s, 3H, H_13_). ^13^C{^1^H} NMR (75 MHz, DMSO-*d*_6_) δ 159.7 (C_g_), 158.1 (C_5_), 155.6 (C_6_), 152.1 (C_1_), 151.1 (C_8_), 141.2 (C_12_), 140.9, 140.5 (C_a_), 139.8 (C_3_), 136.7 (C_f_), 134.9, 133.4, 132.2 (C_9_), 131.7, 129.8 (C_11_), 129.6, 128.7 (C_2_), 128.57, 128.3 (C_p_), 127.9 (C_10_), 127.6 (C_w_), 127.0 (C_k_), 125.8 (C_4+s+d+v+t+l_), 124.7 (C_u_), 124.4 (C_c_), 122.1, 121.0 (C_7_), 118.6 (C_b_), 111.4 (C_e_), 47.2 (C_i_), 20.8 (C_13_).

^19^F{^1^H} NMR (377 MHz, DMSO-*d*_6_): δ −63.14 (s, 3F, CF_3_), −72.95 (d, *J*_P-F_ = 697.1 Hz, 6F, PF_6_). Mass ESI-MS (pos. ion mode, DMSO): calc.: [M-PF_6_]^+^ = 952.2006 *m*/*z*; exp.: 952.2016 *m*/*z*. Anal. calc. for C_47_H_33_ClF_9_IrN_5_P: %C, 51.44; %H, 3.03; %N, 6.38. Found: %C, 51.38; %H, 2.91; %N, 6.28.

##### Ir4

Orange solid. Isolated yield: 83%. ^1^H NMR (400 MHz, DMSO-*d*_6_) δ 9.17 (s, 2H, H_7_), 9.14 (d, *J* = 8.3 Hz, 1H, H_b_), 8.96 (d, *J* = 8.0 Hz, 2H, H_4_), 8.29–8.19 (m, 4H, H_3+10_), 8.01 (d, *J* = 8.3 Hz, 1H, H_e_), 7.90 (dd, *J* = 5.6, 0.8 Hz, 2H, H_1_), 7.76 (d, *J* = 8.3 Hz, 2H, H_l_), 7.60 (ddd, *J* = 7.1, 5.6, 1.0 Hz, 2H, H_2_), 7.57–7.46 (m, 6H, H_11+k+c+d_), 7.29 (d, *J* = 8.3 Hz, 1H, H_s_), 6.45 (d, *J* = 8.3 Hz, 1H, H_r_), 6.33 (s, 2H, H_i_), 5.33 (s, 2H, H_t_), 2.50 (s, 3H, H_13_). ^13^C{^1^H} NMR (101 MHz, DMSO-*d*_6_) δ 161.1 (C_g_), 158.9 (C_5_), 156.6 (C_6_), 152.3 (C_1_), 151.9 (C_p_), 151.1 (C_8_), 146.8 (C_q_), 141.2, 140.9 (C_12_), 140.5 (C_j_), 140.2 (C_3_), 136.3 (C_f_), 132.4 (C_9_), 129.9 (C_11_), 128.9 (C_2_), 128.4, 128.1 (C_h_), 127.9 (C_10_), 126.9 (C_k_), 125.9 (C_l_), 125.5 (C_4_), 124.3 (C_c_), 124.1 (C_d_), 122.8, 121.7 (C_s_), 120.3 (C_7_), 118.2 (C_b_), 116.1 (C_o_), 111.2 (C_e_), 104.6 (C_r_), 99.8 (C_t_), 47.2 (C_i_), 20.9 (C13). ^19^F{^1^H} NMR (377 MHz, DMSO-*d*_6_): δ −63.14 (s, 3F, CF_3_), −72.97 (d, *J*_P-F_ = 702.4 Hz, 6F, PF_6_). Mass ESI-MS (pos. ion mode, DMSO): calc.: [M-PF_6_]^+^ = 946.1748 *m*/*z*; exp.: 946.1771 *m*/*z*. Anal. calc. for C_44_H_31_ClF_9_IrN_5_O_2_P: %C, 48.42; %H, 2.86; %N, 6.42. Found: %C, 48.57; %H, 3.09; %N, 6.27.

##### Ir5

Orange solid. Isolated yield: 62%. ^1^H NMR (600 MHz, DMSO-*d*_6_) δ 9.26 (s, 2H, H_7_), 8.98 (d, *J* = 8.1 Hz, 2H, H_4_), 8.83 (d, *J* = 8.1 Hz, 1H, H_b_), 8.26 (d, *J* = 8.3 Hz, 2H, H_10_), 8.22 (td, *J* = 8.1, 1.4 Hz, 2H, H_3_), 7.91 (d, *J* = 8.2 Hz, 1H, H_e_), 7.84 (d, *J* = 5.3 Hz, 2H, H_1_), 7.61 (d, *J* = 4.9 Hz, 1H, H_q_), 7.57–7.52 (m, 4H, H_11+2_), 7.49 (ddd, *J* = 8.2, 7.2, 0.9 Hz, 1H, H_d_), 7.41 (ddd, *J* = 8.1, 7.2, 0.9 Hz, 1H, H_c_), 6.16 (d, *J* = 4.9 Hz, 1H, H_p_), 4.29 (s, 3H, H_i_), 2.50 (s, 3H, H_13_). ^13^C{^1^H} NMR (151 MHz, DMSO-*d*_6_) δ 159.7 (C_g_), 158.3 (C_5_), 156.1 (C_6_), 152.7 (C_1_), 151.3 (C_8_), 145.2 (C_h_), 141.3 (C_12_), 141.0 (C_a_), 140.0 (C_3_), 135.3 (C_f_), 132.4 (C_9_), 132.0 (C_q_), 129.9 (C_11_), 128.9 (C_2_), 128.8 (C_p_), 128.1 (C_10_), 125.7 (C_4_), 124.0 (C_o_), 123.8 (C_c_), 122.9 (C_d_), 121.1 (C_7_), 116.8 (C_b_), 111.4 (C_e_), 31.8 (C_i_), 21.0 (C_13_). Mass ESI-MS (pos. ion mode, DMSO): calc.: [M-PF_6_]^+^ = 764.1227 *m*/*z*; exp.: 764.1227 *m*/*z*. Anal. calc. for C_34_H_26_ClF_6_IrN_5_PS: %C, 44.91; %H, 2.88; %N, 7.70; %S, 3.53. Found: %C, 45.08; %H, 2.91; %N, 7.58; %S, 3.62.

##### Ir6

Orange solid. Isolated yield: 57%. ^1^H NMR (600 MHz, DMSO-*d*_6_) δ 9.27 (s, 2H, H_7_), 9.05 (d, *J* = 8.4 Hz, 1H, H_b_), 8.99 (d, *J* = 8.0 Hz, 2H, H_4_), 8.28 (d, *J* = 7.8 Hz, 2H, H_10_), 8.20 (ddd, *J* = 8.0, 7.3, 1.2 Hz, 2H, H_3_), 8.04 (d, *J* = 8.2 Hz, 2H, H_e+s_), 7.80 (d, *J* = 6.0 Hz, 2H, H_1_), 7.56 (m, 3H, H_d+11_), 7.50 (ddd, *J* = 7.3, 6.0, 1.2 Hz, 2H, H_2_), 7.45 (ddd, *J* = 8.4, 7.6, 0.9 Hz, 1H, H_c_), 7.00 (ddd, *J* = 8.4, 7.8, 0.8 Hz, 1H, H_r_), 6.80 (ddd, *J* = 8.4, 7.8, 0.8 Hz, 1H, H_q_), 6.14 (dd, *J* = 7.8 Hz, 1H, H_p_), 4.48 (s, 3H, H_i_), 2.50 (s, 3H, H_13_). ^13^C{^1^H} NMR (151 MHz, DMSO-*d*_6_) δ 161.1 (C_g_), 158.2 (C_5_), 155.8 (C_6_), 152.4 (C_1_), 151.2 (C_8_), 142.0 (C_o_), 141.3 (C_12_), 140.3 (C_a_), 139.9 (C_3_), 136.3 (C_f_), 134.4 (C_h_), 132.4 (C_9_), 130.9 (C_p_), 130.3 (C_q_), 130.0 (C_11_), 128.8 (C_2_), 128.1 (C_10_), 126.1 (C_s_), 125.9 (C_4_), 123.8 (C_c_), 123.7 (C_d+r_), 121.1 (C_7_), 118.0 (C_b_), 111.5 (C_e_), 32.4 (C_i_), 21.0 (C_13_). ^19^F{^1^H} NMR (377 MHz, DMSO-*d*_6_): δ −72.96 (d, *J*_P-F_ = 707.0 Hz, 6F, PF_6_). Mass ESI-MS (pos. ion mode, DMSO): calc.: [M-PF_6_]^+^ = 758.1663 *m*/*z*; exp.: 758.1664 *m*/*z*. Anal. calc. for C_36_H_28_ClF_6_IrN_5_P: %C, 47.87; %H, 3.12; %N, 7.75. Found: %C, 47.82; %H, 3.36; %N, 7.78.

##### Ir7

Orange solid. Isolated yield: 66%. ^1^H NMR (600 MHz, DMSO-*d*_6_) δ 9.31 (s, 2H, H_7_), 9.12 (d, *J* = 8.2 Hz, 1H, H_b_), 9.00 (d, *J* = 8.2 Hz, 2H, H_4_), 8.66 (s, 1H, H_s_), 8.32 (d, *J* = 8.4 Hz, 2H, H_10_), 8.19 (td, *J* = 8.2, 1.1 Hz, 2H, H_3_), 8.11 (ddd, *J* = 8.5, 7.2, 0.9 Hz, 1H, H_e_), 7.96 (dd, *J* = 8.2, 1.5 Hz, 1H, H_w_), 7.86 (dd, *J* = 5.7, 1.1 Hz, 2H, H_1_), 7.62 (dd, *J* = 7.2, 1.0 Hz, 1H, H_d_), 7.59 (d, *J* = 8.4 Hz, 2H, H_11_), 7.50–7.45 (m, 3H, H_c+2_), 7.36 (dd, *J* = 7.6, 1.3 Hz, 1H, H_t_), 7.33–7.26 (m, 2H, H_v+u_), 6.49 (s, 1H, H_p_), 4.62 (s, 3H, H_i_), 2.52 (s, 3H, H_13_). ^13^C{^1^H} NMR (151 MHz, DMSO-*d*_6_) δ 160.1 (C_g_), 158.3 (C_5_), 155.9 (C_6_), 152.3 (C_1_), 151.2 (C_8_), 141.3 (C_12_), 140.5 (C_a_), 139.9 (C_3_), 136.7 (C_f_), 135.0 (C_o_), 133.6 (C_q_), 133.0 (C_h_), 132.4 (C_9_), 130.1 (C_r_), 130.0 (C_11_), 129.0 (C_w_), 128.7 (C_2_), 128.4 (C_p_), 128.1 (C_10_), 127.5 (C_u_), 126.0 (C_s_), 125.9 (C_t_), 124.8 (C_v_), 124.1 (C_c_), 123.9 (C_d_), 121.1 (C_7_), 118.3 (C_b_), 111.6 (C_e_), 32.7 (C_i_), 21.0 (C_13_). Mass ESI-MS (pos. ion mode, DMSO): calc.: [M-PF_6_]^+^ = 808.1819 *m*/*z*; exp.: 808.1829 *m*/*z*. Anal. calc. for C_40_H_30_ClF_6_IrN_5_P: %C, 50.40; %H, 3.17; %N, 7.35. Found: %C, 50.46; %H, 3.18; %N, 7.23.

##### Ir8

Orange solid. Isolated yield: 59%. ^1^H NMR (600 MHz, DMSO-*d*_6_) δ 9.15 (s, 2H, H_7_), 9.00 (d, *J* = 8.2 Hz, 1H, H_b_), 8.94 (d, *J* = 8.1 Hz, 2H, H_4_), 8.25–8.19 (m, 4H, H_3+10_), 7.98 (d, *J* = 8.2 Hz, 1H, H_e_), 7.80 (dd, *J* = 6.1, 1.0 Hz, 2H, H_1_), 7.70 (d, *J* = 8.3 Hz, 1H, H_s_), 7.56–7.49 (m, 5H, H_11+2+d_), 7.40 (ddd, *J* = 8.2, 7.4, 0.8 Hz, 1H, H_c_), 6.62 (d, *J* = 8.3 Hz, 1H, H_r_), 5.36 (s, 2H, H_t_), 4.40 (s, 3H, H_i_), 2.50 (s, 3H, H_13_). ^13^C{^1^H} NMR (151 MHz, DMSO-*d*_6_) δ 161.3 (C_g_), 159.0 (C_5_), 156.7 (C_6_), 152.3 (C_1_), 152.0 (C_p_), 151.1 (C_8_), 146.7 (C_q_), 141.2 (C_12_), 140.4 (C_a_), 140.2 (C_3_), 136.3 (C_f_), 132.5 (C_9_), 130.0 (C_11_), 129.3 (C_h_), 128.8 (C_2_), 128.0 (C_10_), 125.5 (C_4_), 123.7 (C_c_), 123.5 (C_d_), 122.0 (C_s_), 120.4 (C_7_), 117.8 (C_b_), 115.6 (C_o_), 111.3 (C_e_), 104.6 (C_r_), 99.8 (C_t_), 32.4 (C_i_), 21.0 (C_13_). Mass ESI-MS (pos. ion mode, DMSO): calc.: [M-PF_6_]^+^ = 802.1560 *m*/*z*; exp.: 802.1559 *m*/*z*. Anal. calc. for C_37_H_28_ClF_6_IrN_5_O_2_P: %C, 46.91; %H, 2.98; %N, 7.39. Found: %C, 46.92; %H, 3.02; %N, 7.40.

##### Ir9

Orange solid. Isolated yield: 59%. ^1^H NMR (400 MHz, DMSO-*d*_6_) δ 9.77 (d, *J* = 0.8 Hz, 1H, H_b_), 9.27 (s, 2H, H_7_), 8.99 (d, *J* = 7.9 Hz, 2H, H_4_), 8.28 (d, *J* = 7.9 Hz, 2H, H_10_), 8.22 (dt, *J* = 7.9, 1.0 Hz, 2H, H_3_), 8.19–8.13 (m, 2H, H_d+e_), 7.92 (d, *J* = 7.7 Hz, 1H, H_s_), 7.83 (dd, *J* = 5.6, 1.0 Hz, 2H, H_1_), 7.56 (d, *J* = 8.0 Hz, 2H, H_11_), 7.49 (ddd, *J* = 7.9, 5.6, 1.2 Hz, 2H, H_2_), 7.04 (dt, *J* = 7.7, 0.9 Hz, 1H, H_r_), 6.82 (dt, *J* = 7.7, 0.8 Hz, 1H, H_q_), 6.18 (dd, *J* = 7.7, 0.9 Hz, 1H, H_p_), 4.95 (t, *J* = 6.9 Hz, 2H, H_i_), 3.86 (s, 3H, H_u_), 2.50 (s, 3H, H_13_), 2.00 (m, 2H, H_j_), 1.58 (m, 2H, H_k_), 0.99 (t, *J* = 7.2 Hz, 3H, H_l_). ^13^C{^1^H} NMR (101 MHz, DMSO-*d*_6_) δ 166.4 (C_t_), 162.6 (C_g_), 158.1 (C_5_), 155.7 (C_6_), 152.5 (C_1_), 151.3 (C_8_), 143.2 (C_o_), 141.4 (C_12_), 140.2 (C_a_), 140.0 (C_3_), 139.3 (C_f_), 133.4 (C_h_), 132.3 (C_9_), 131.2 (C_p_), 130.9 (C_q_), 130.0 (C_11_), 129.0 (C_2_), 128.1 (C_10_), 126.2 (C_s_), 125.9 (C_4_), 125.6 (C_c_), 124.7 (C_d_), 124.1 (C_r_), 121.2 (C_7_), 120.2 (C_b_), 111.9 (C_e_), 52.3 (C_u_), 44.8 (C_i_), 31.4 (C_j_), 21.0 (C_13_), 19.5 (C_k_), 13.9 (C_l_). ^19^F{^1^H} NMR (377 MHz, DMSO-*d*_6_): δ −72.96 (d, *J*_P-F_ = 707.0 Hz, 6F, PF_6_). Mass ESI-MS (pos. ion mode, DMSO): calc.: [M-PF_6_]^+^ = 858.2186 *m*/*z*; exp.: 858.2172 *m*/*z*. Anal. calc. for C_41_H_36_ClF_6_IrN_5_O_2_P: %C, 49.08; %H, 3.62; %N, 6.98. Found: %C, 49.21; %H, 3.57; %N, 6.97.

### Methods and Instrumentation

#### Microwave

Complexes were synthesized in a 10 mL vial in an Anton Paar Monowave 50 (315 W) microwave.

#### Nuclear Magnetic Resonance (NMR) Spectroscopy

The ^1^H, ^13^C{^1^H}, and bidimensional NMR spectra were recorded on a Bruker AC 300E, Bruker AV 400, or Bruker AV 600 NMR spectrometer, and chemical shifts were determined by reference to the residual ^1^H and ^13^C{^1^H} solvent peaks.

#### Elemental Analysis

The C, H, N, and S analyses were performed with a Carlo Erba model EA 1108 microanalyzer with EAGER 200 software.

#### Mass Spectrometry (MS)

ESI mass (positive mode) analyses were performed on an RP/MS TOF 6220. The isotopic distribution of the heaviest set of peaks matched very closely to that calculated for formulating the complex cation in every case.

#### Photophysical Characterization

UV/Vis spectroscopy was performed on a PerkinElmer Lambda 750 S spectrometer with operating software. Solutions of all complexes were prepared in acetonitrile and water (1% DMSO) at 10 μM. The emission spectra were obtained with a Horiba Jobin Yvon Fluorolog 3-22 modular spectrofluorometer with a 450 W xenon lamp. Measurements were performed in a right-angled configuration using 10 mm quartz fluorescence cells for solutions at 298 K. Emission lifetimes (τ) were measured using an IBH FluoroHub TCSPC controller and a NanoLED (372 nm) pulse diode excitation source (τ < 10 μs); the estimated uncertainty is ±10% or better. Emission quantum yields (Φ) were measured using a Hamamatsu C11347 absolute PL quantum yield spectrometer; the estimated uncertainty is ±10% or better. Solutions of all complexes were prepared in acetonitrile at 10 μM. For lifetimes and quantum yield measurements, the samples were previously degassed by bubbling argon for 20 min.

#### Stability in Cell Culture Medium

The stability of complexes in the cell culture medium was evaluated by the UV/Vis spectra at *t* = 0 and after 24 h at 37 °C. The solutions were prepared in RPMI (5% DMSO) at 10 μM.

### Antiproliferative Activity

The antiproliferative activity of the investigated compounds was evaluated using the SRB assay. The cells were seeded in 96-well tissue culture plates at a density of 3 × 10^3^ cells/well in 100 μL of the medium. After overnight incubation in a humidified CO_2_ incubator, the cells were treated with the tested compounds in a final volume of 200 μL/well. After an additional 72 h, cells were washed with PBS and fixed for 1 h at 4 °C with trichloroacetic acid. Then, the cells were stained with 0.4% SRB dye for 30 min. Stained cells were dissolved with Tris (10 mM). The cell viability was evaluated by measurement of the absorbance at 570 nm using an absorbance reader (SPARK TECAN, SCHOELLER). The reading values were converted to the percentage of control (% cell survival). Antiproliferative effects were expressed as IC_50_ values calculated from curves constructed by plotting cell survival (%) versus drug concentration (μM) (IC_50_ = concentration of the agent inhibiting cell growth by 50%). Concentrations of compounds present in the medium during the treatment were verified by FAAS or ICP-MS.

### Trypan Blue Exclusion Test

MDA-MB-231 or MDA-MB-231 VIM RFP cells were seeded at the same density and format of used culture plastic, depending on each experiment. After the overnight incubation, the cells were treated with the concentrations of the investigated compounds corresponding to IC_50,72h_ and further incubated for 24 h. Then, samples were collected by trypsinization, and an equal amount of 0.4% trypan blue (Bio-Rad, USA) was added to the cell suspension. Finally, samples were analyzed for cell viability on an automated TC10 cell counter (Bio-Rad, USA).

### Cellular Uptake

The cellular accumulation of studied complexes was determined on the MDA-MB-231 cell line. Cells were seeded on 100 mm Petri dishes at the density of 1.5 × 10^6^ cells per dish. After overnight pre-incubation, the cells were exposed to the investigated Ir(III) compounds for 24 h. After the treatment, the cells were harvested, counted, washed twice with ice-cold PBS, and collected by centrifugation. Cells were also analyzed for cell viability using a trypan blue exclusion test to verify the condition of harvested samples and exclude the possible cell membrane permeability impairment. Finally, the cell pellets were digested using a microwave acid (HCl, 5 M) digestion system (CEM Mars). The quantity of metal taken up by the cells was determined by ICP-MS.

### Measurement of the Partition Coefficient (Log *P*)

The partition coefficient log *P* was measured by the “shake-flask” method. Water and *n*-octanol were pre-saturated with *n*-octanol and water, respectively, with the addition of NaCl (100 mM). Compounds were dissolved in the water phase, and the defined volume of the solution was mixed with water-saturated octanol in a volumetric ratio of 1:6. The samples were exhaustively vortexed overnight at room temperature to establish the equilibrium. To separate the phases, the samples were centrifuged at 3000*g* for 5 min. The iridium concentration in the water phase ([Ir]_osw_) was determined by ICP-MS; the iridium concentration in the octanol phase ([Ir]_wso_) was calculated as the difference between the initial concentration and the concentration in the water phase after shaking, and the resulting log *P* was calculated by using the following formula: log *P* = log([Ir]_WSO_/[Ir]_OSW_)·(1/6)] = log([Ir]_init_ – [Ir]_OSW_)/[Ir]_OSW_)·(1/6)].

### Cellular Localization Study

MDA-MB-231 cells were seeded on 35 mm glass-bottom confocal culture dishes (Mattek Co., MA, USA) at 1 × 10^5^ cells/dish density and incubated overnight. Then, the cells were treated with tested compounds (5 μM) and incubated for 5 h. After incubation, cells were co-stained with MitoTracker (Thermo Fisher Scientific, Waltham, MA, USA). Samples were fixed with 3.7% formaldehyde, mounted with ProLong Diamond Antifade Mountant medium (Thermo Fisher Scientific), and then analyzed on a confocal laser-scanning microscope Leica TCS SP5 (Leica Microsystems GmbH, Wetzlar, Germany). The investigated Ir complexes were excited at 355 nm. Samples were scanned sequentially. Colocalization analysis of acquired images was performed using ImageJ software. Briefly, the Pearson coefficient of correlation (PCC) was measured for entire images by default, and the Costes regression method was used to estimate the threshold. Values of PCC are expressed as the mean from two independent experiments ± SDs.

### Detection of Cell Death

Cell death was determined using the standard annexin V/PI assay and analyzed by flow cytometry. Briefly, MDA-MB-231 cells were seeded on 96-well plates at 3 × 10^4^ cells/well density. After the overnight incubation, the cells were treated with **1** at the concentration corresponding to the multiple of IC_50,72h_ (1×, 2×, and 3× IC_50,72h_) or with other compounds **2**–**9** at 2× IC_50,72h_ and incubated for a further 18 h. Vehicle controls were treated with 1% DMSO. In addition, positive controls for apoptosis were treated for 3 h with 1 μM staurosporine, whereas controls for necrosis positivity were treated for 1 h with 10% EtOH. After that, samples were washed with PBS and detached using non-enzymatic harvesting with CellStriper (Corning). Samples were transferred to annexin V binding buffer and stained with annexin V Pacific Blue (Thermo Fisher Scientific)/PI (Merck, 1 μg mL^–1^) staining solution. After 15 min of incubation at RT, samples were analyzed by a flow cytometer Cell Stream (Luminex, USA). The data were processed in Cell Stream analysis software or FCS Express 7 (DeNovo Software, CA). At least 30,000 events were analyzed for each sample made in triplicate, and the experiment was independently repeated four times.

### Flow Cytometric Analysis of the Cell Cycle

MDA-MB-231 cells were seeded at the density of 5 × 10^5^ cells per well in six-well culture plates. After overnight pre-incubation in a drug-free medium, the cells were treated for 24 h with the tested Ir(III) terdentate compounds and doxorubicin at their final concentrations corresponding to 2× IC_50,72 h_ values. Samples were harvested by trypsinization, washed twice with PBS, resuspended in 70% ethanol, and kept at 4 °C overnight. Fixed cells were rinsed twice in PBS and stained with propidium iodide diluted to 50 μg mL^–1^ in Vindel’s solution (10 mM Tris–Cl, pH 8.0, 10 mM NaCl, 0.1% Triton X-100, 100 μg mL^–1^ RNase A) for 30 min at 37 °C. Cell cycle profiles were measured with a FACS Verse flow cytometer (Becton Dickinson, Germany). Data analysis was processed in FCS Express 7 (DeNovo Software, CA). Results were confirmed in two independent experiments. Each analysis consists of at least 20,000 single cells, gated by using the FCS-H to FCS-A method. Data were submitted to statistical analysis using the Student’s *t*-test, and the significant differences (*P* ≤ 0.05) from vehicle DMSO-treated control were marked with a star.

### Anti-Invasion Activity Determined by Real-Time Monitoring of the Cell Growth

Anti-invasion activity was monitored by the xCELLigence system (Roche). This system enables the monitoring of invading cells in real time and thus provides more detailed data about the antimigratory effects stimulated after the treatment with tested compounds. MDA-MB-231 cells were treated in a six-well plate with the concentration of tested compounds corresponding to IC_50,72h._ After the 24 h incubation period, cells were trypsinized and seeded at the density of 1 × 10^4^ cells/well on the top well of the sandwich chamber system. Before the cell seeding, the inserts (top chambers) of the sandwich chamber system were coated with the Geltrex matrix (Gibco) and left for 1 h at 37 °C to coat the chamber surface. Cells (top chambers) were seeded in a culture medium without FBS, supplemented with 0.1% bovine serum albumin. The bottom chambers were filled with the complete medium, supplemented with 10% FBS (chemoattractant). Samples were continuously monitored every 5 min for an additional 7 days. The time of cell invasion was defined as the time point where the invading cells exceeded the cell index (CI) above zero. The period necessary for invasion through the Geltrex matrix could more precisely define the anti-invasion effects of tested compounds on MDA-MB-231 cells.

### Flow Cytometric Quantification of Vimentin

Quantification of vimentin expression after the treatment of MDA-MB-231 VIM RFP cells with tested compounds was determined with flow cytometry. MDA-MB-231 VIM RFP cells were seeded at the density of 1 × 10^4^ cells/well and incubated overnight. After that, cells were treated with the concentration of compounds corresponding to the respective multiple of IC_50,72h_ (0.25×, 0.5×, 1×, and 2×). After 72 h of incubation, cells were trypsinized and analyzed on a flow cytometer Cell Stream (Luminex, USA). The data were processed using Cell Stream analysis software or FCS Express 7 (DeNovo Software, CA). The fluorescence coming from the VIM RFP tag is proportional to the expression of vimentin in treated cells. Each sample analyzed by flow cytometry consisted of at least 30,000 events.

### RT-qPCR of the EMT-Related Gene Transcripts

MDA-MB-231 cells were seeded on a six-well plate at the density of 0.5 × 10^6^ cells per well and incubated overnight. Then, the cells were treated with equitoxic concentrations of tested compounds corresponding to IC_50,72h_ and incubated for 24 h. Cells were harvested, and total RNA was isolated using NucleoSpin RNA columns (Machery Nagel, GE). One-step qPCR combining reverse transcription followed by amplification thermal cycling was applied using a Luna Universal One-Step RT-qPCR (New England BioLabs, MA, USA). Experiments were performed on an Illumina Eco Real-Time PCR (Illumina, CA, USA). The thermal profile was as follows: reverse transcription for 10 min at 55 °C and initial denaturation for 1 min at 95 °C, followed by 43 thermal cycles of denaturation for 10 s at 95 °C and extension for 30 s at 60 °C. Primer sequences were as follows: Β-actin-F: CACCATTGGCAATGAGCGGTTC; Β-actin-R: AGGTCTTTGCGGATGTCCACGT; Vim-F: AGGCAAAGCAGGAGTCCACTGA; Vim-R: ATCTGGCGTTCCAGGGACTCAT; E-Cadherin-F: GCCTCCTGAAAAGAGAGTGGAAG; E-Cadherin-R: TGGCAGTGTCTCTCCAAATCCG; N-Cadherin-F: CCTCCAGAGTTTACTGCCATGAC; N-Cadherin-R: GTAGGATCTCCGCCACTGATTC; B-Catenin-F: CACAAGCAGAGTGCTGAAGGTG; B-Catenin-R: GATTCCTGAGAGTCCAAAGACAG; SNAI1-F: TGCCCTCAAGATGCACATCCGA; SNAI1-R: GGGACAGGAGAAGGGCTTCTC. Purified primers were from Generi Biotech (Czech Republic). All runs included melting curve analysis and template-free negative technical controls to confirm specific single-product amplification. Β-actin was used as an internal control. The relative expression of mRNA is represented as a fold increase (2^–ΔΔ*C*t^).^69^

### Morphology of Treated MDA-MB-231 Cells and Qualitative Analysis of the Vimentin Filament Network Determined by Confocal Microscopy

MDA-MB-231 VIM RFP cells were seeded on 35 mm confocal Petri dishes (Mattek) at the density of 2.5 × 10^5^ cells per dish and incubated overnight. Then, the cells were treated with tested compounds at the concentration corresponding to IC_50,72h_ and incubated for a further 24 h. Then, samples were washed with PBS, fixed with 3.7% formaldehyde (10 min, RT), and mounted with ProLong Diamond Antifade Mountant with DAPI. Samples were visualized with a confocal microscope Leica SP8 SMD operated under the deconvolution lightning mode.

### Morphological Quantification of Tumor Spheroids and Quantification of Cell-Invasive Potential

MDA-MB-231 cells were seeded in a sandwich agar matrix (0.5% bottom gel and 0.35% upper cell-containing gel). First, wells of 24-well plates were covered with 0.5% agar gel, and then MDA-MB-231 cells were resuspended in 0.35% agar matrix. The detailed protocol was as follows: Stock solution of agar gel was prepared by autoclaving 1 g of agar in 100 mL of PBS for at least a 20 min liquid cycle. Twenty-four-well plates were covered with 1 mL of 0.5% agar created by mixing 1% agar solution with cultivation media in 1:1 ratio. The dish was then layered with 1 mL of 0.35% agar composed of 0.35 mL of 1% agar and 0.65 mL of cultivation media containing 10^5^ MDA-MB-231 cells. After gel solidification, the dish was covered with 2 mL of cultivation media. Mixing of agar solution and media was carried out after the agar solution cooled to approximately 40 °C to prevent damage by high temperature. Cells were cultured for an additional 7 days to establish spheroid structures, then treated with tested compounds at the equitoxic concentration corresponding to IC_50,72h_, and cultured for an additional 72 h. Samples were photographed on a phase contrast microscope (Olympus CKX41), and images were processed and analyzed for morphology parameters in ImageJ software.

### Wound Healing Assay

MDA-MB-231 VIM RFP cells were seeded on 35 mm confocal Petri dishes (Mattek) at the density of 3 × 10^5^ cells/dish and incubated overnight or for a further 24–48 h to reach approximately 70% confluency. Defined scratch (220 μm width) was done under continuous suction with the pipette tip. Cells were washed, and the medium was replaced with the culture medium supplemented with tested compounds, applied at the equitoxic concentration (IC_50,72h_). Samples were incubated for an additional 24 h before fixation with 3.7% formaldehyde (10 min, RT) and mounted with ProLong Diamond Antifade Mountant (LifeSciences, Thermo Fisher Scientific). Samples were visualized with a confocal microscope Leica SP8 SMD operated under the deconvolution lightning mode. Images were analyzed by using ImageJ software. The scratch boundaries were identified with the MRI wound healing tool ImageJ plugin or identified manually.

## Supporting Information Available

Synthetic schemes of the diamine **B1**, nuclear magnetic resonance (NMR), mass spectrometry, and stability studies (PDF)
Molecular formula strings and biological data (CSV)

## Abbreviations Used

CI: cell index
DMSO: dimethyl sulfate
EMT: epithelial–mesenchymal transition
FBS: fetal bovine serum
IC_50_: concentration of the agent inhibiting cell growth by 50%
ICP-MS: inductively coupled plasma mass spectrometry
LLCT: ligand-to-ligand charge transfer
MET: mesenchymal-to-epithelial transition
MLCT: metal-to-ligand charge transfer
mRNA: messenger RNA
RFP: red fluorescent protein
RTCA: real-time cell analyzer
RT-qPCR: reverse transcription quantitative PCR
SRB: sulforhodamine B
SI: selectivity index
TCRP: time-dependent cell response profile
TNBC: triple-negative breast cancer
VIF: vimentin intermediate filament
