# Supplementary material for: Ir(III) Compounds Containing a Terdentate Ligand Are Potent Inhibitors of Proliferation and Effective Antimetastatic Agents in Aggressive Triple-Negative Breast Cancer Cells
Source: J Med Chem. 2023 Jul 6;66(14):9766–83. doi: 10.1021/acs.jmedchem.3c00586 (PMC10388354; doi:10.1021/acs.jmedchem.3c00586)
Supplement: Supplementary file 1 — jm3c00586_si_001.pdf [file jm3c00586_si_001.pdf]

Supporting Information

Ir(III) compounds containing a terdentate ligand are potent inhibitors of proliferation and effective antimetastatic agents in aggressive triple-negative breast cancer cells

Vojtech Novohradsky[a,1], Alicia Marco [b,1], Lenka Markova[a], Natalia Cutillas[b], José Ruiz[b,*], Viktor Brabec[b,*]

[a] *Czech Academy of Sciences, Institute of Biophysics, Kralovopolska 135, CZ-61200 Brno, Czech Republic*
[b] *Departamento de Química Inorgánica, Universidad de Murcia, and Institute for Bio-Health Research of Murcia (IMIB-Arrixaca), E-30100 Murcia, Spain*

[1] These authors contributed equally to this work.

Table of Contents

1. Synthetic scheme of the diamine **B**	S1
2. Nuclear Magnetic Resonance (NMR)	S2–S35
3. Mass spectrometry	S35–S41
4. High performance liquid chromatography (HPLC) analysis	S42–S44
5. Photophysical properties	S45
6. Stability studies	S45–S50
7. Statistical analysis of the antiproliferative activity	S54-S55
8. Intracellular localization, cell death and cell cycle analysis	S56-S58
9. Reference	S59

1. Synthetic scheme of diamine B

The diamine **B** (Scheme S1) was obtained as previously described.[1]

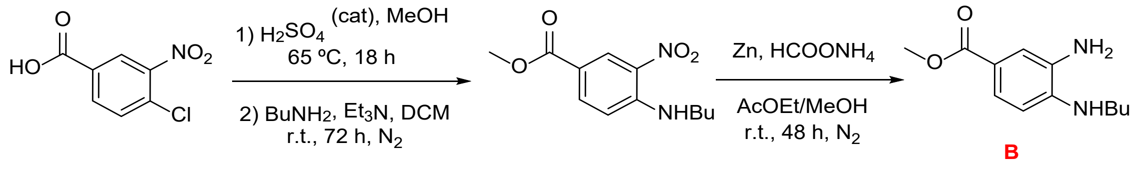

Scheme S1. Synthetic procedure for the diamine **B**.

2. Nuclear Magnetic Resonance (NMR)

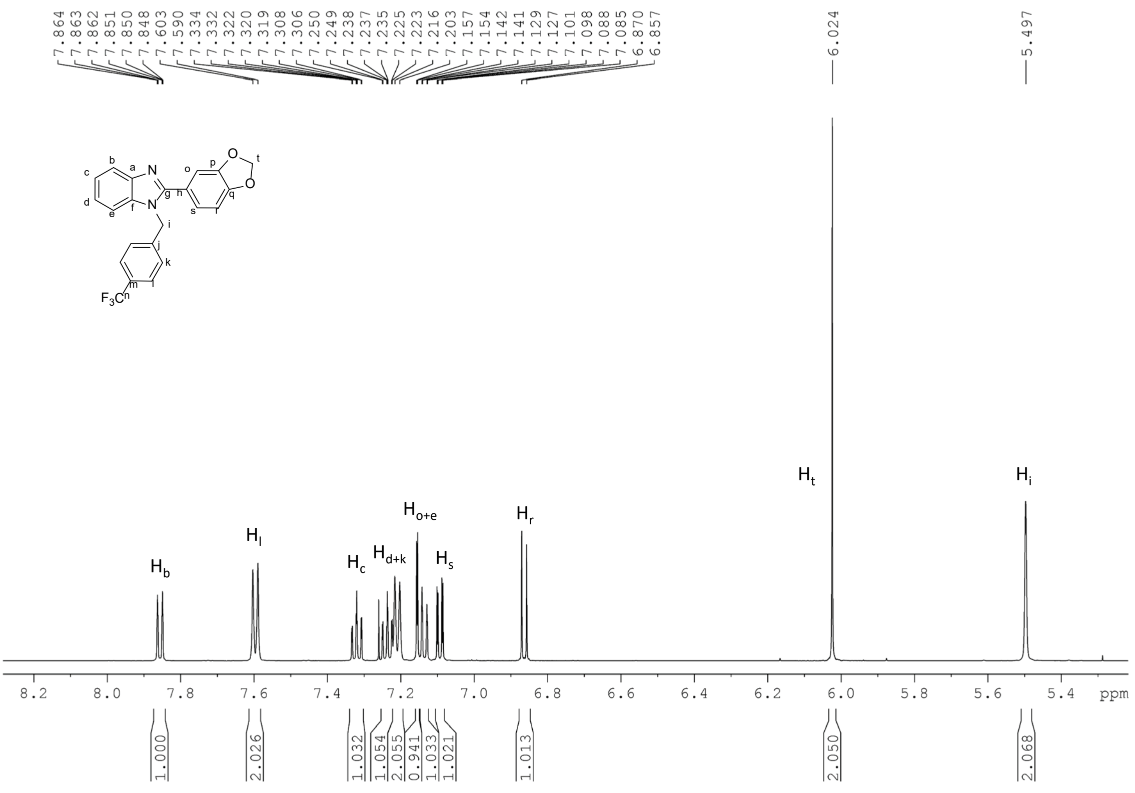

Figure S1. ^{1}H NMR spectrum of **HL4**, 400 MHz, $CDCl_3$.

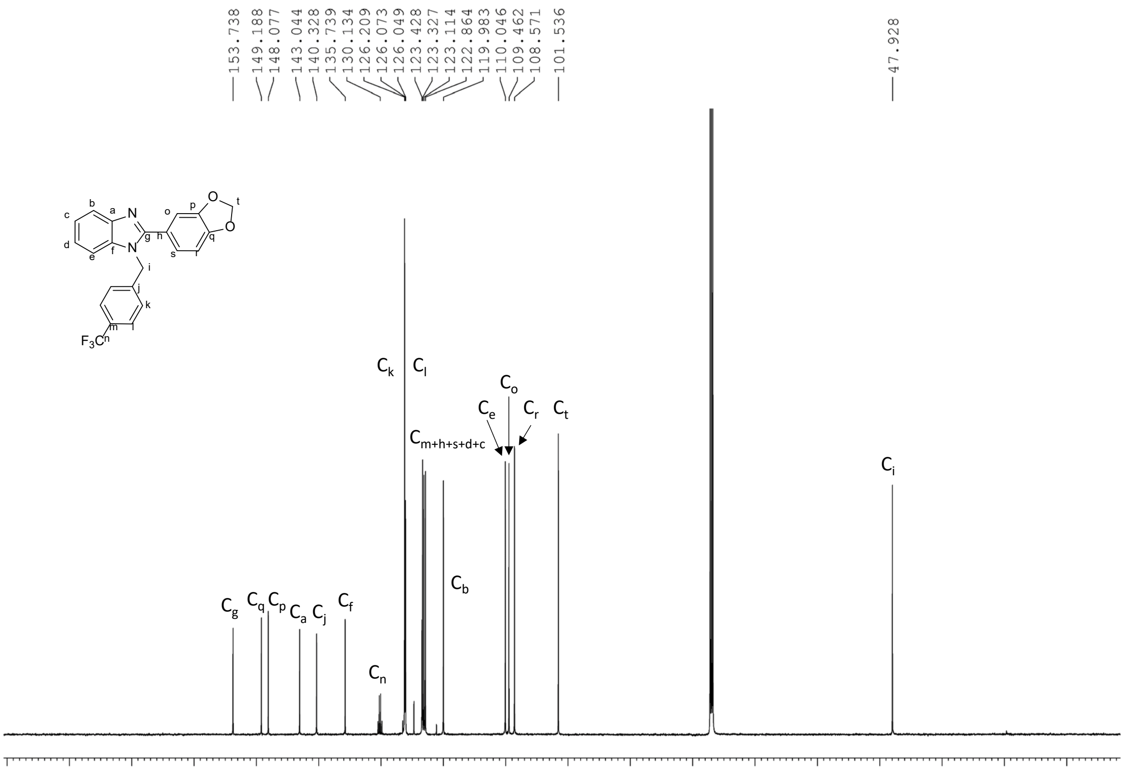

Figure S2. ^{13}C NMR spectrum of **HL4**, 101 MHz, $CDCl_3$.

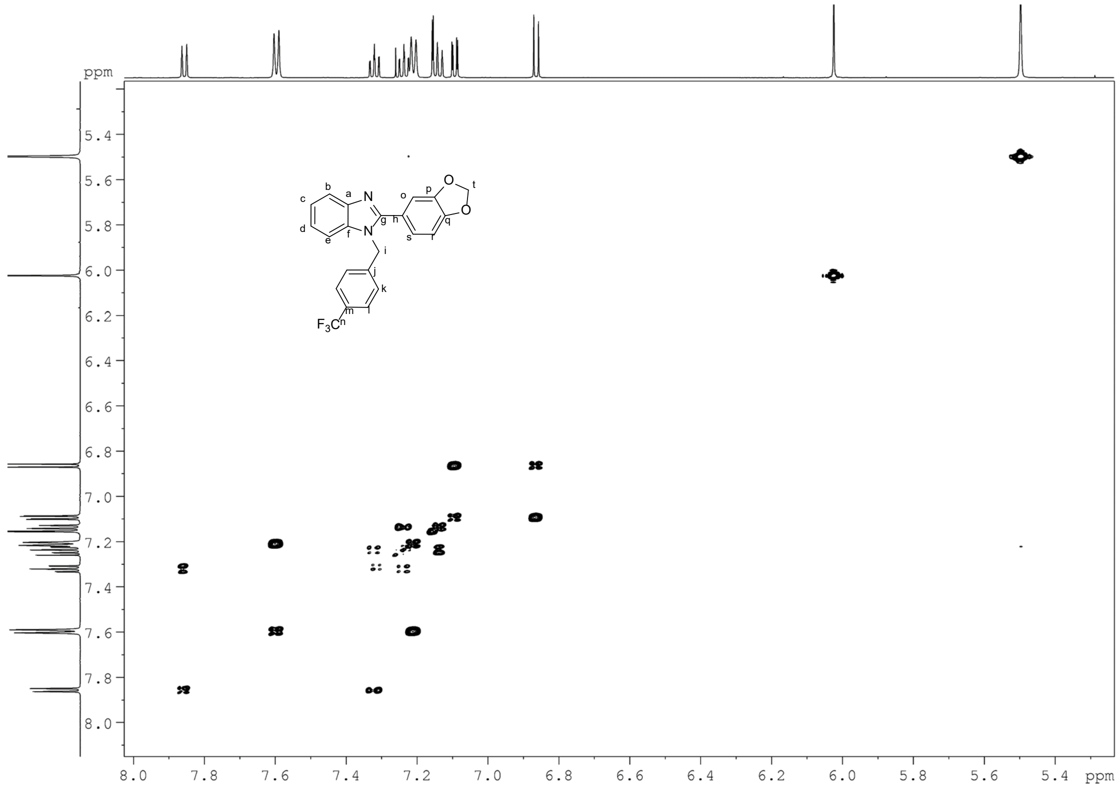

Figure S3. ^{1}H-^{1}H COSY NMR spectrum of **HL4**, 400 MHz, $CDCl_3$.

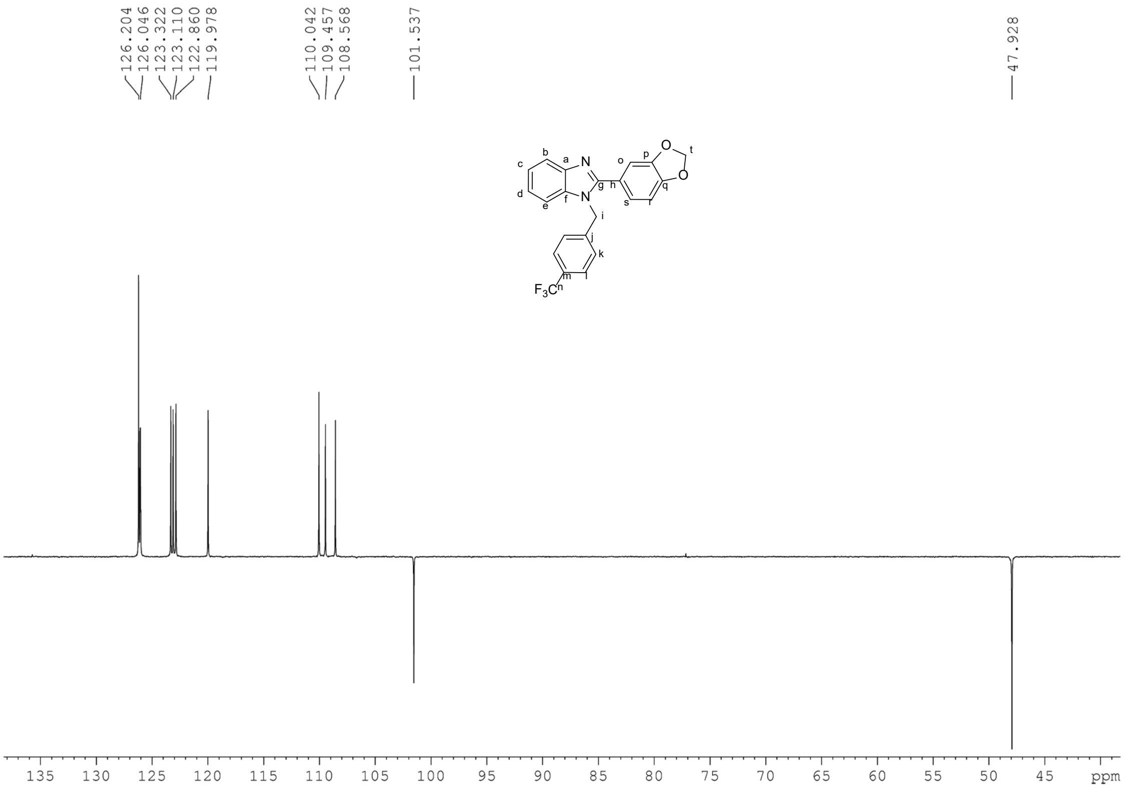

Figure S4. DEPT-135 NMR spectrum of **HL4**, 101 MHz, $CDCl_3$.

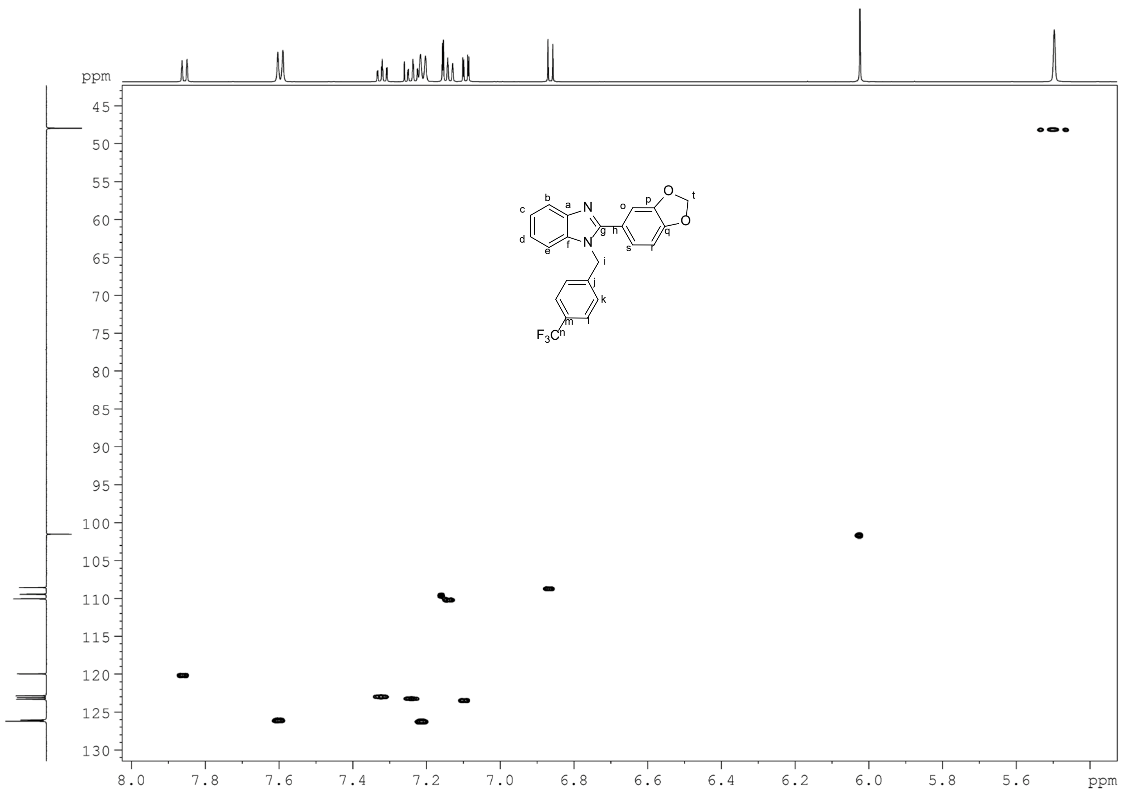

Figure S5. ^{1}H-^{13}C HSQC NMR spectrum of **HL4**, 400 MHz, $CDCl_3$.

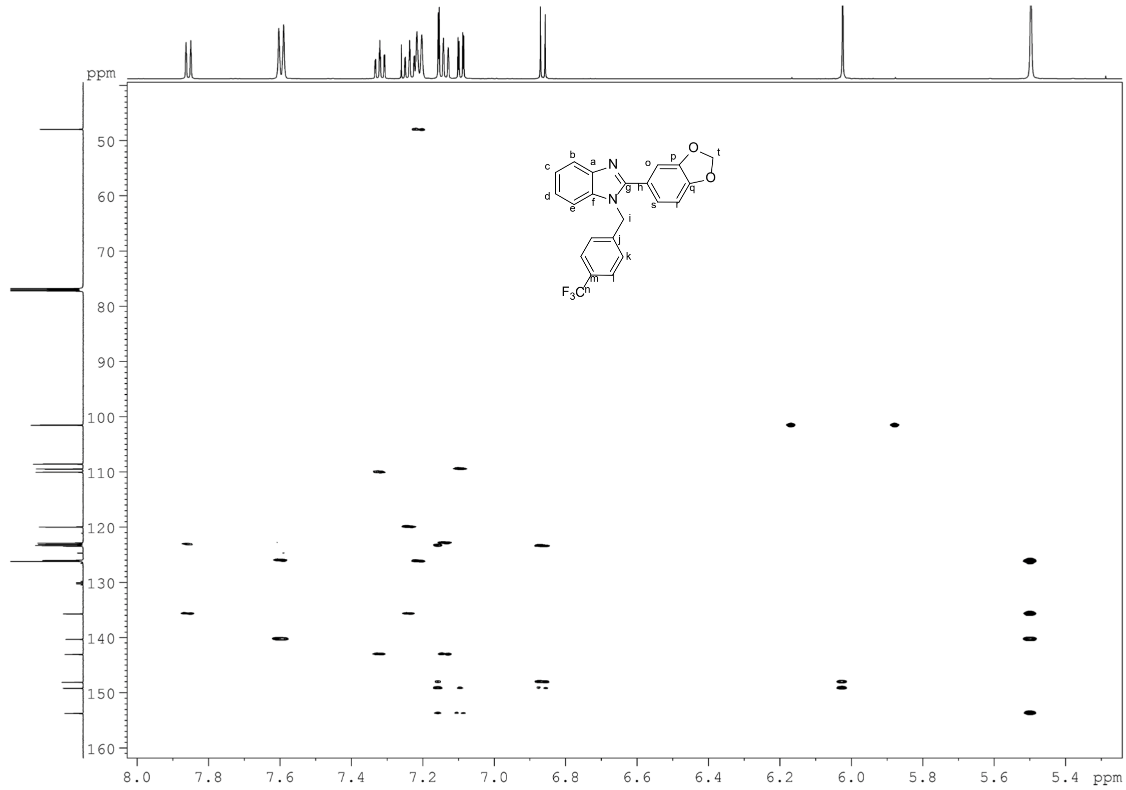

Figure S6. ^{1}H-^{13}C HMBC NMR spectrum of **HL4**, 400 MHz, $CDCl_3$.

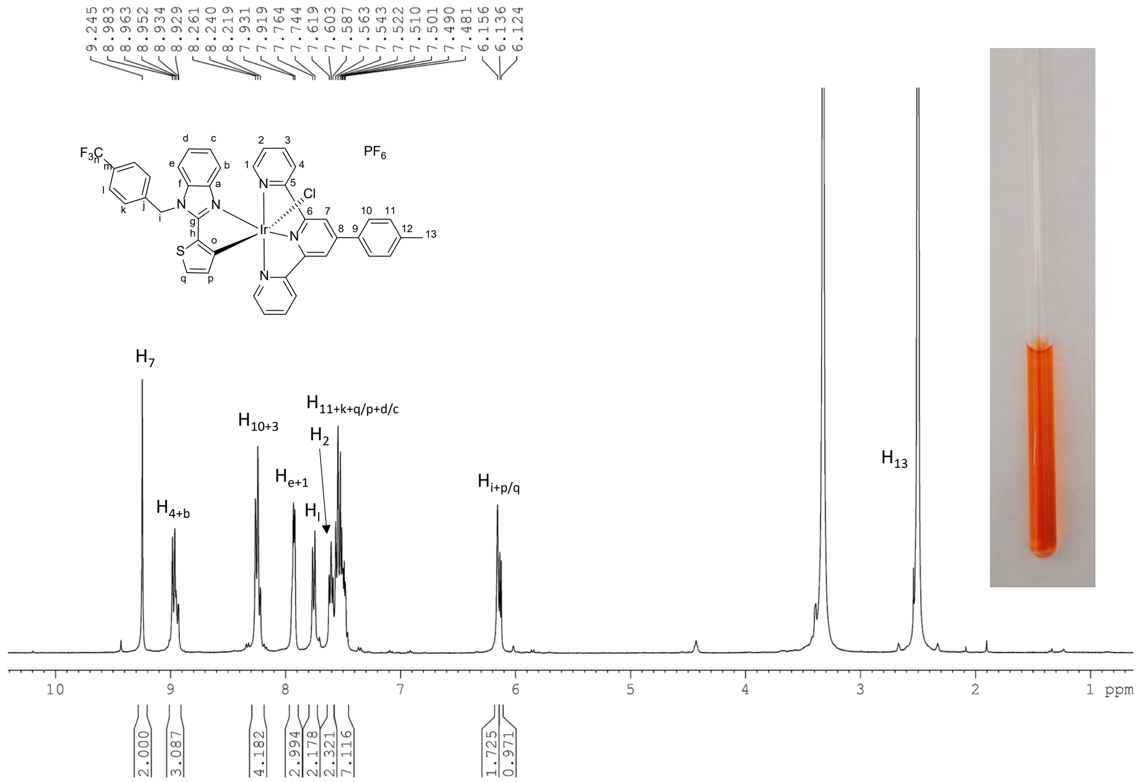

Figure S7. ^{1}H NMR spectrum of **Ir1**, 400 MHz, DMSO-d_6.

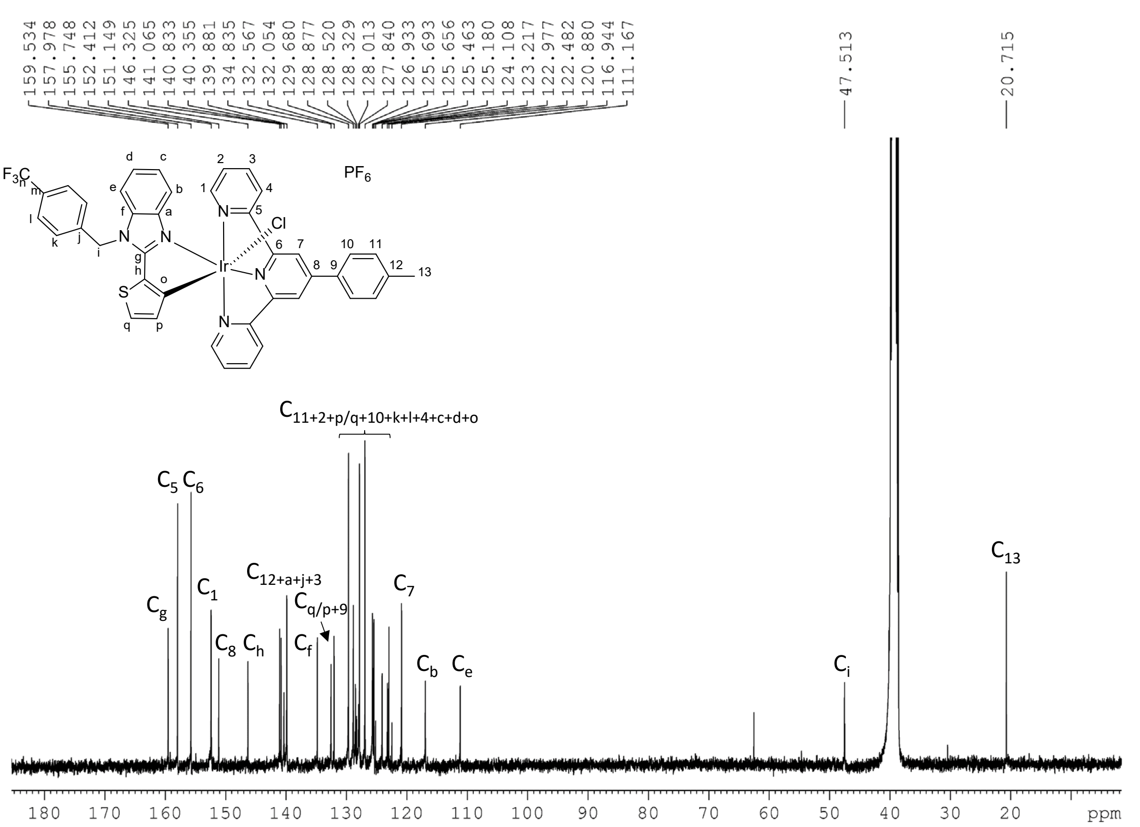

Figure S8. ^{13}C NMR spectrum of **Ir1**, 101 MHz, DMSO-d_6.

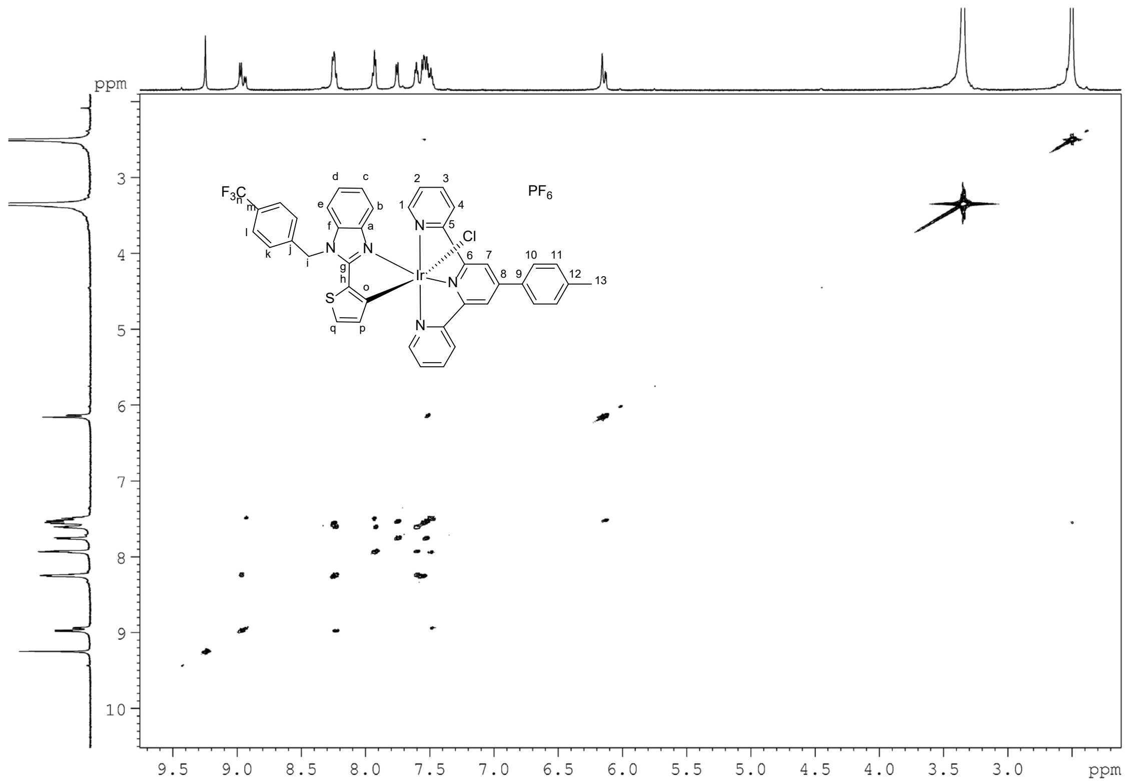

Figure S9. ^1H-^1H COSY NMR spectrum of **Ir1**, 600 MHz, DMSO-d_6.

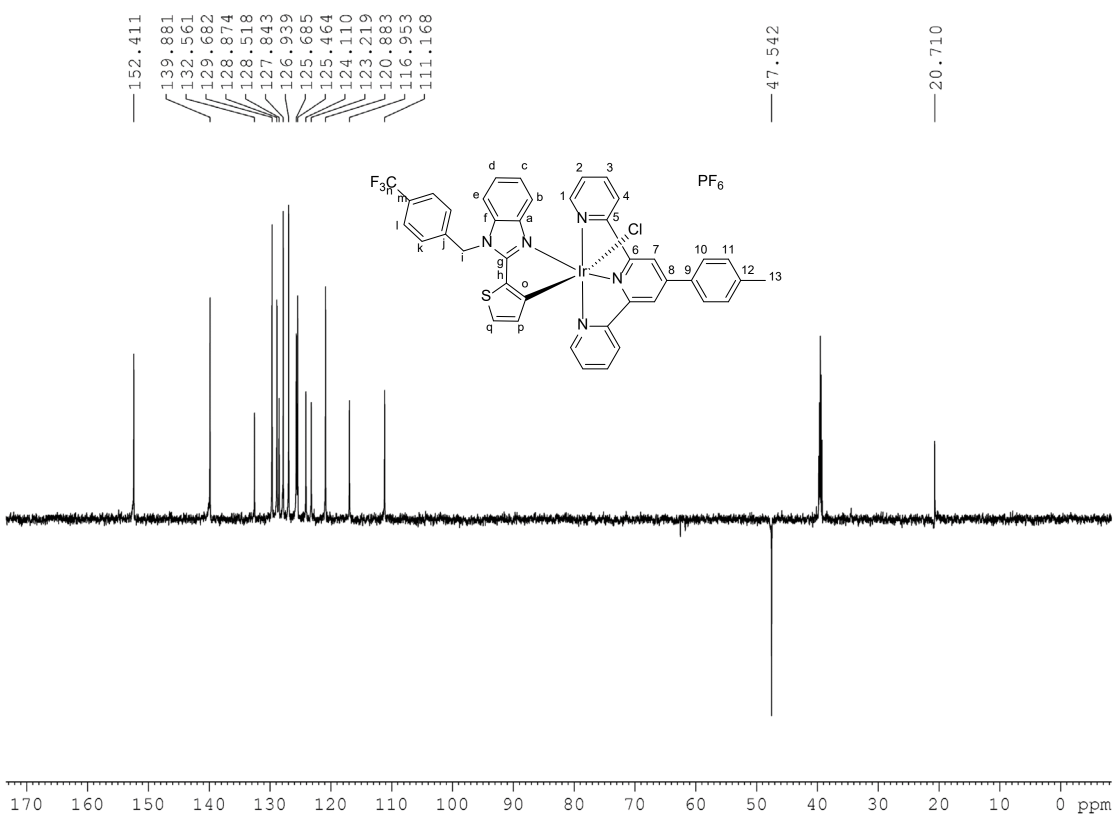

Figure S10. DEPT-135 NMR spectrum of **Ir1**, 101 MHz, DMSO-d_6.

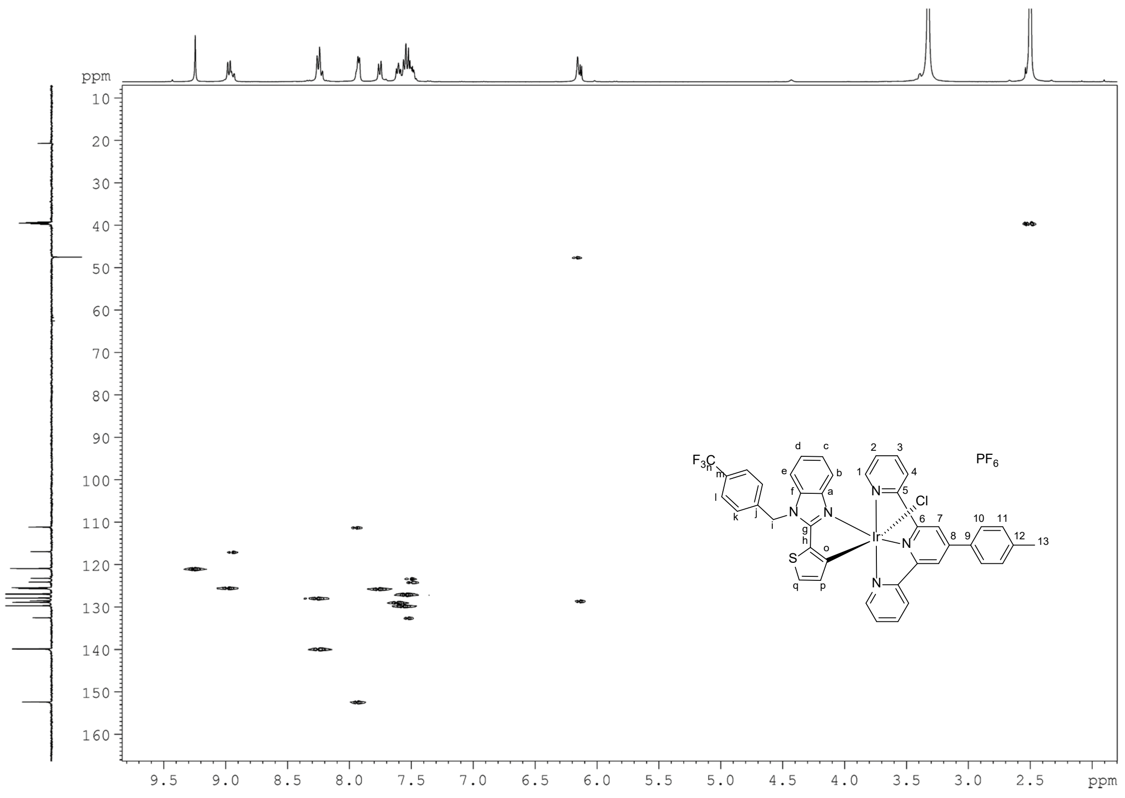

Figure S11. ^{1}H-^{13}C HSQC NMR spectrum of **Ir1**, 600 MHz, DMSO-*d$_6$*.

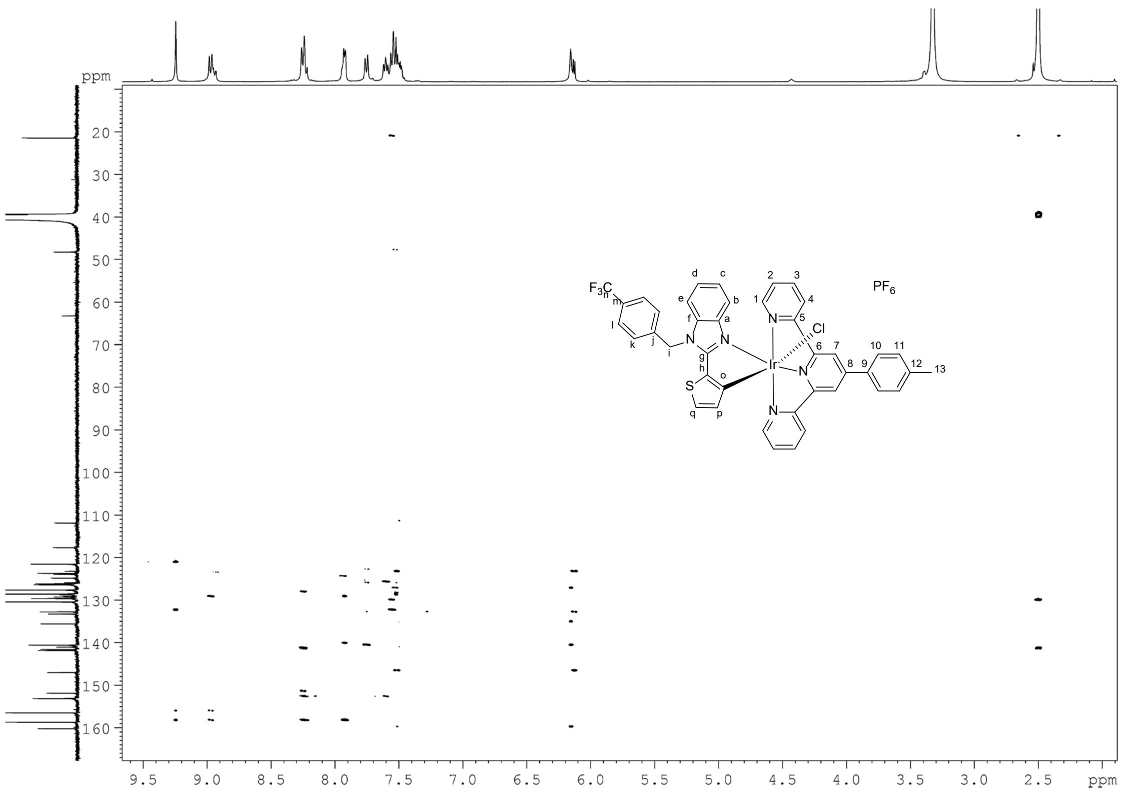

Figure S12. ^{1}H-^{13}C HMBC NMR spectrum of **Ir1**, 600 MHz, DMSO-*d$_6$*.

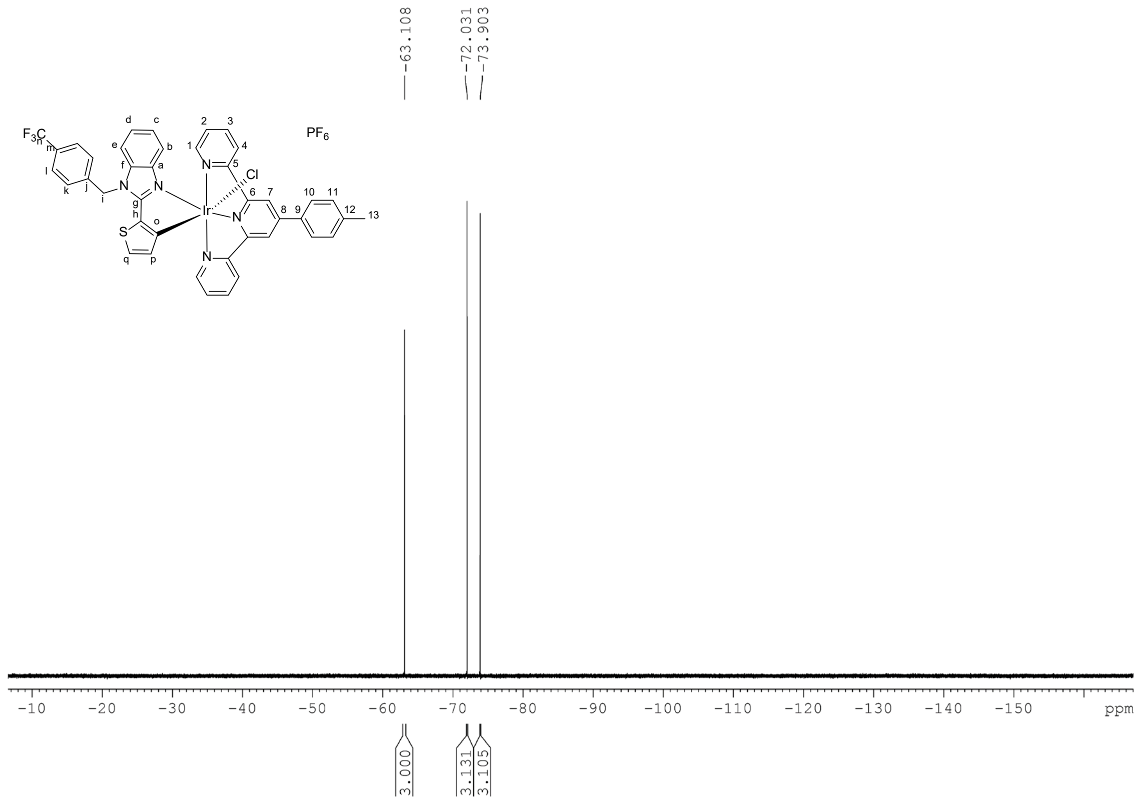

Figure S13 ^{19}F NMR spectrum of **Ir1**, 377 MHz, DMSO-d_6.

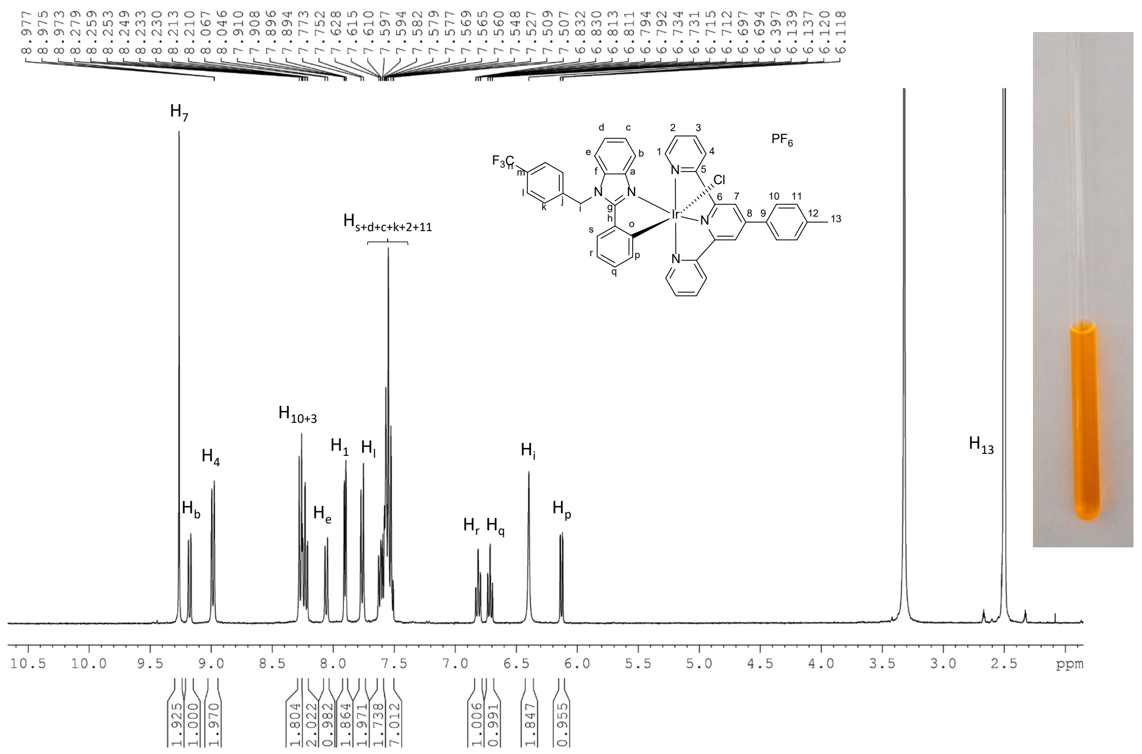

Figure S14. ^{1}H NMR spectrum of **Ir2**, 400 MHz, DMSO-d_6.

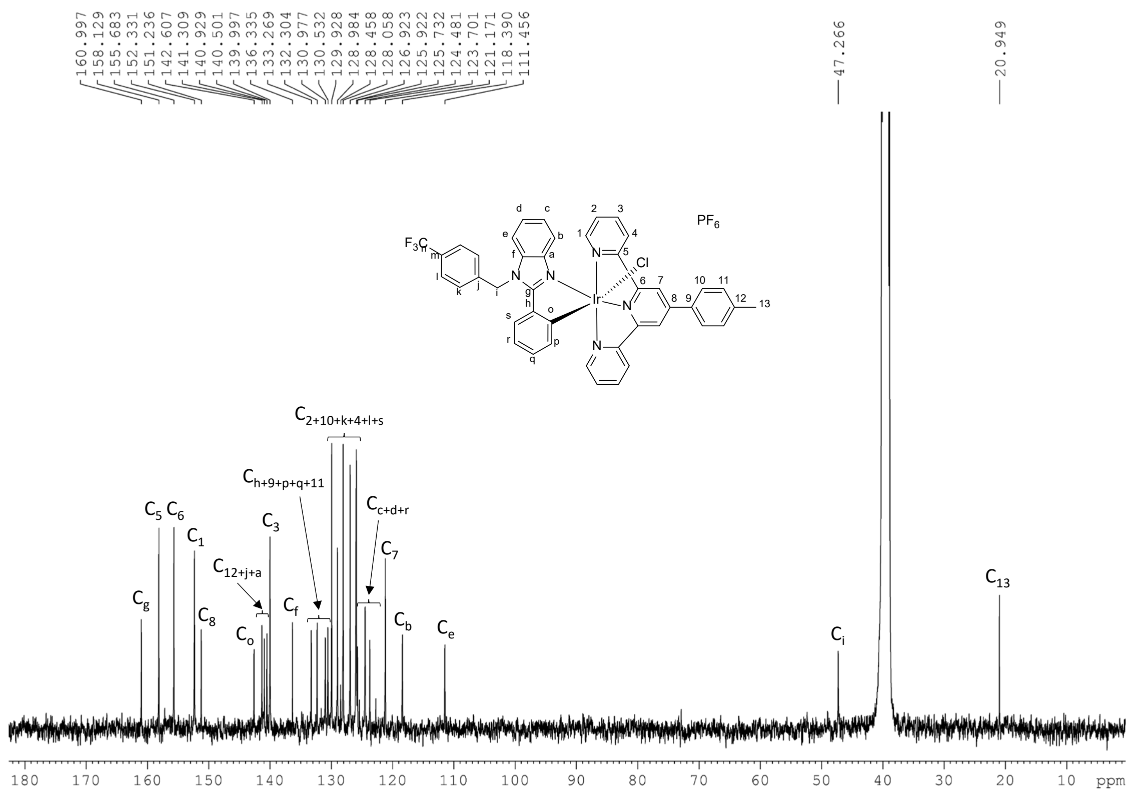

Figure S15. ^{13}C NMR spectrum of **Ir2**, 101 MHz, DMSO-d_6.

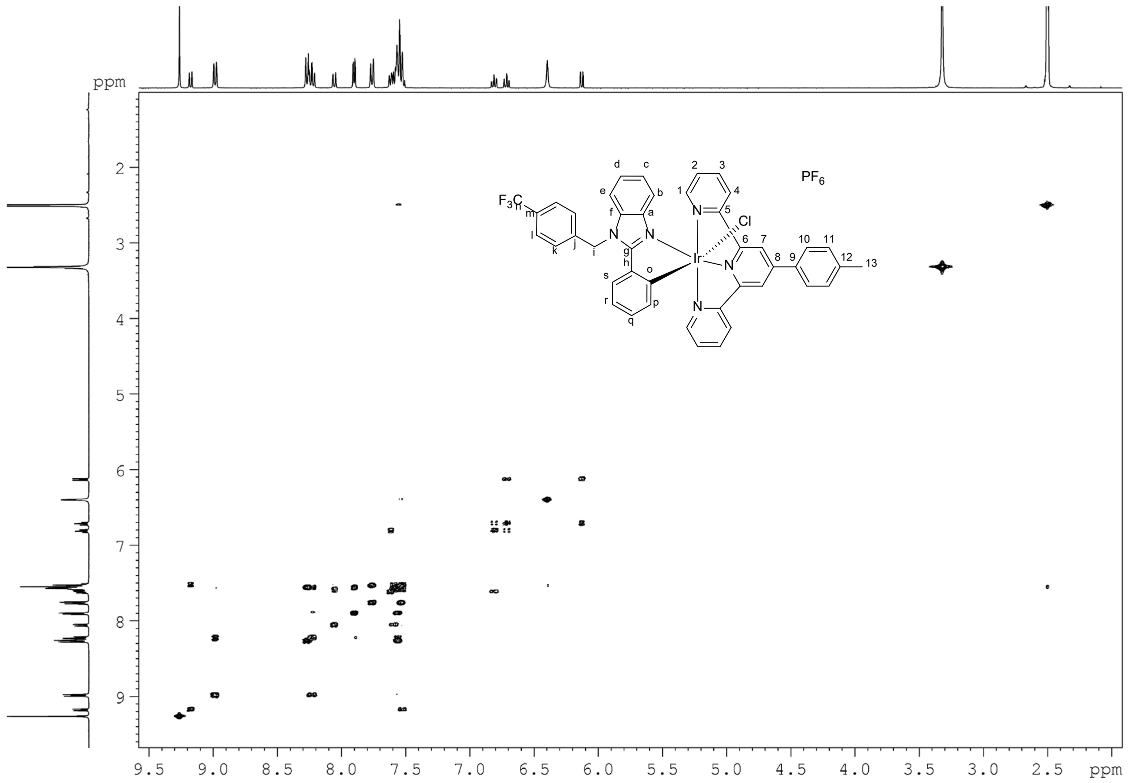

Figure S16. ^{1}H-^{1}H COSY NMR spectrum of **Ir2**, 600 MHz, DMSO-d_6.

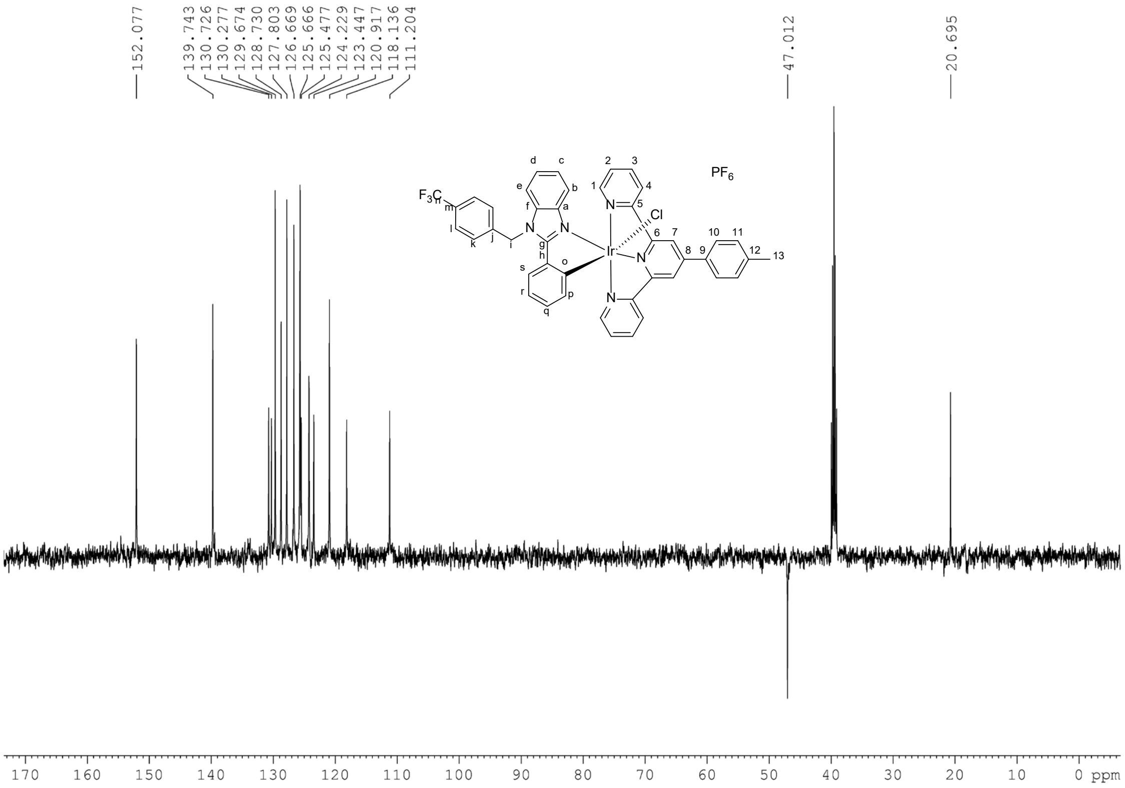

Figure S17. DEPT-135 NMR spectrum of **Ir2**, 101 MHz, DMSO-d_6.

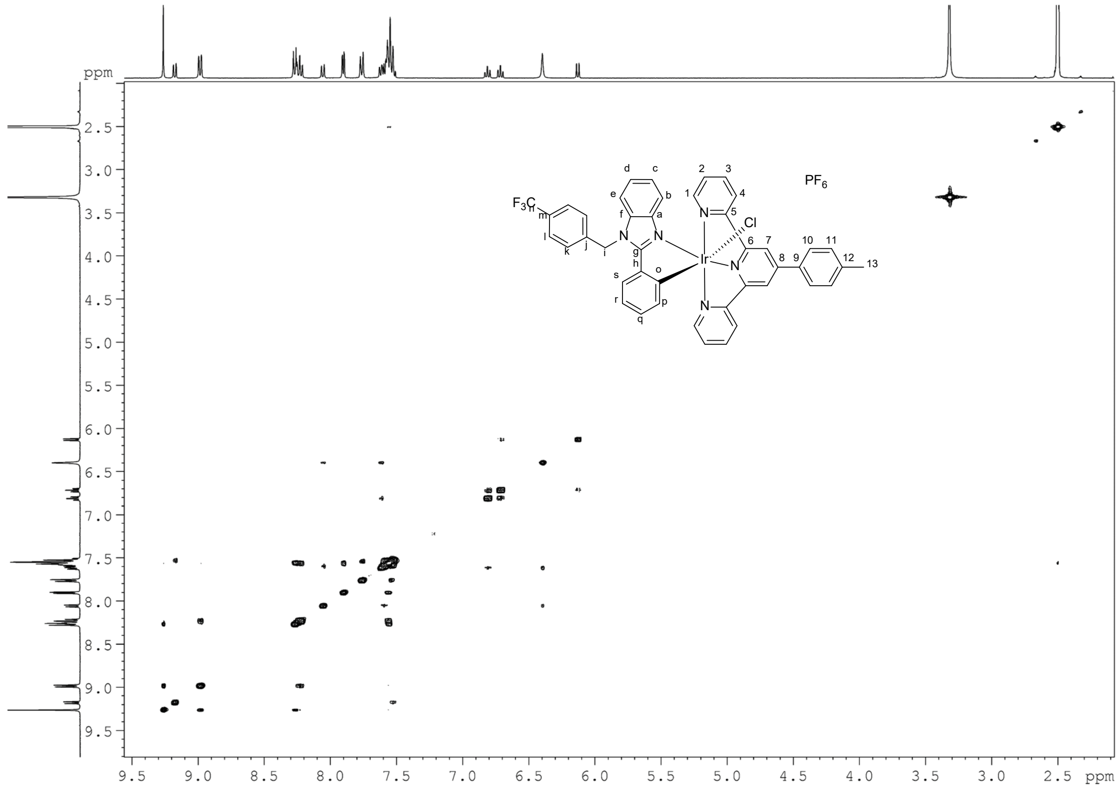

Figure S18. ^{1}H-^{1}H NOESY NMR spectrum of **Ir2**, 600 MHz, DMSO-d_6.

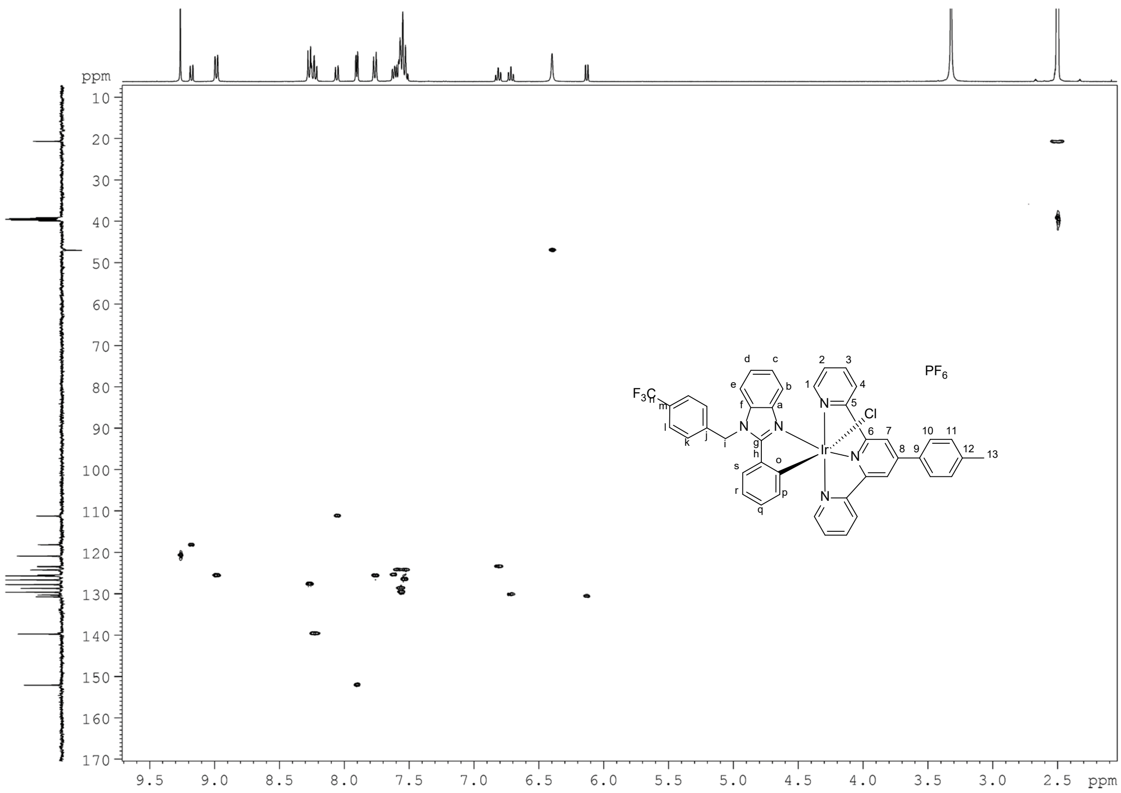

Figure S19. ^{1}H-^{13}C HSQC NMR spectrum of **Ir2**, 600 MHz, DMSO-d_6.

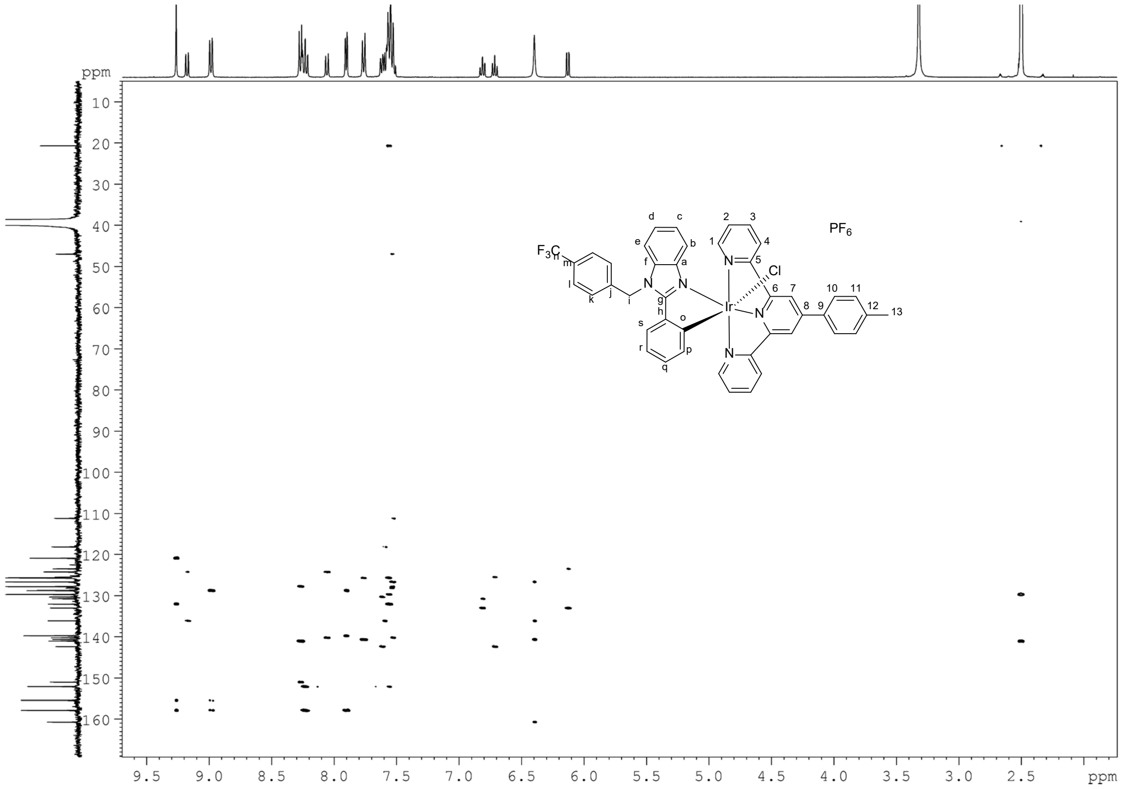

Figure S20. ^{1}H-^{13}C HMBC NMR spectrum of **Ir2**, 600 MHz, DMSO-d_6.

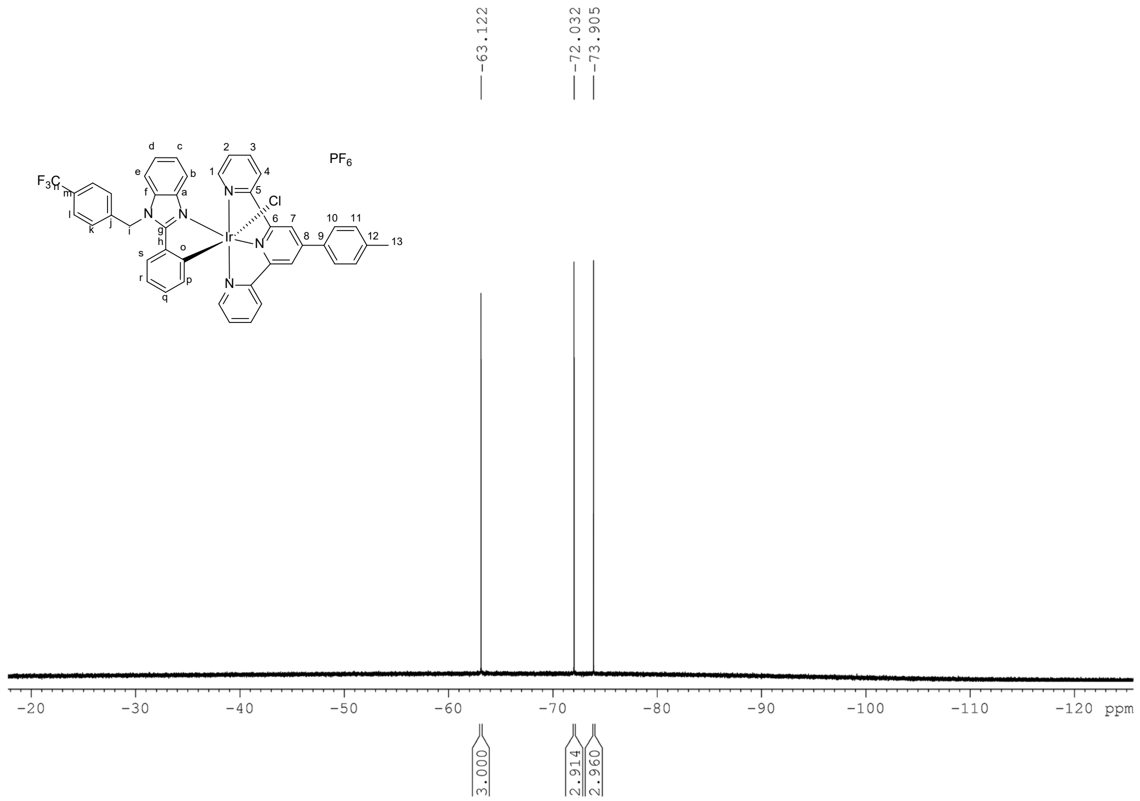

Figure S21. ^{19}F NMR spectrum of **Ir2**, 377 MHz, DMSO-d_6.

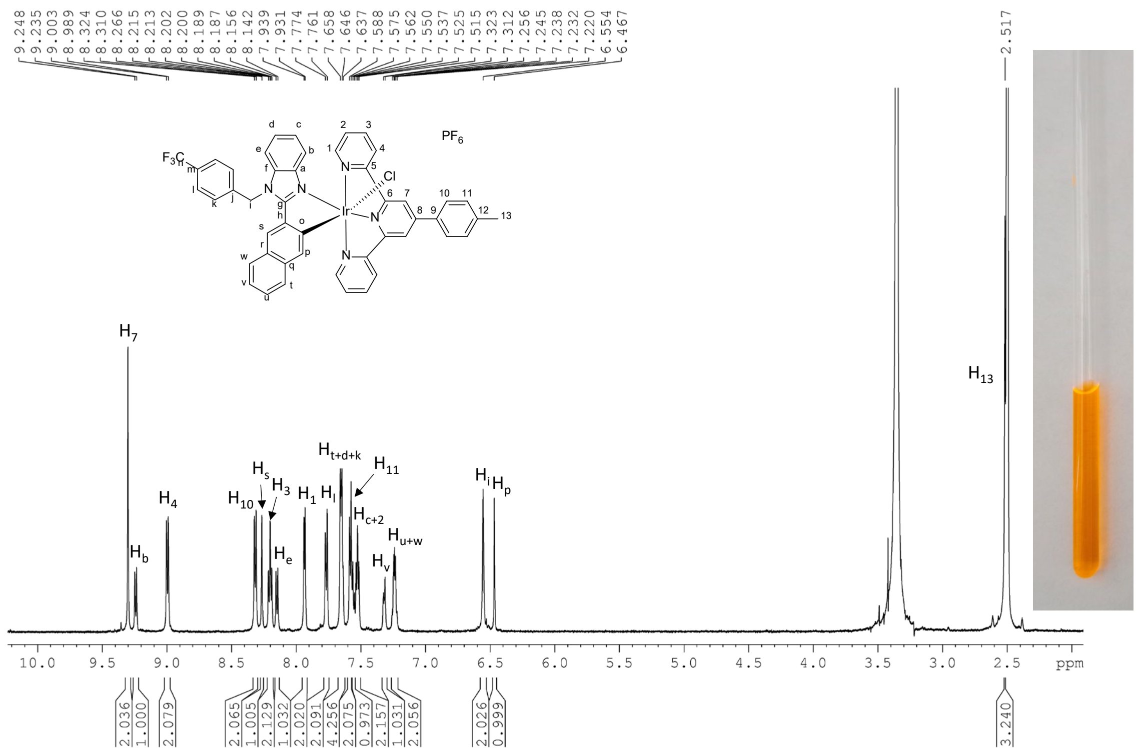

Figure S22. ^{1}H NMR spectrum of **Ir3**, 300 MHz, DMSO-d_6.

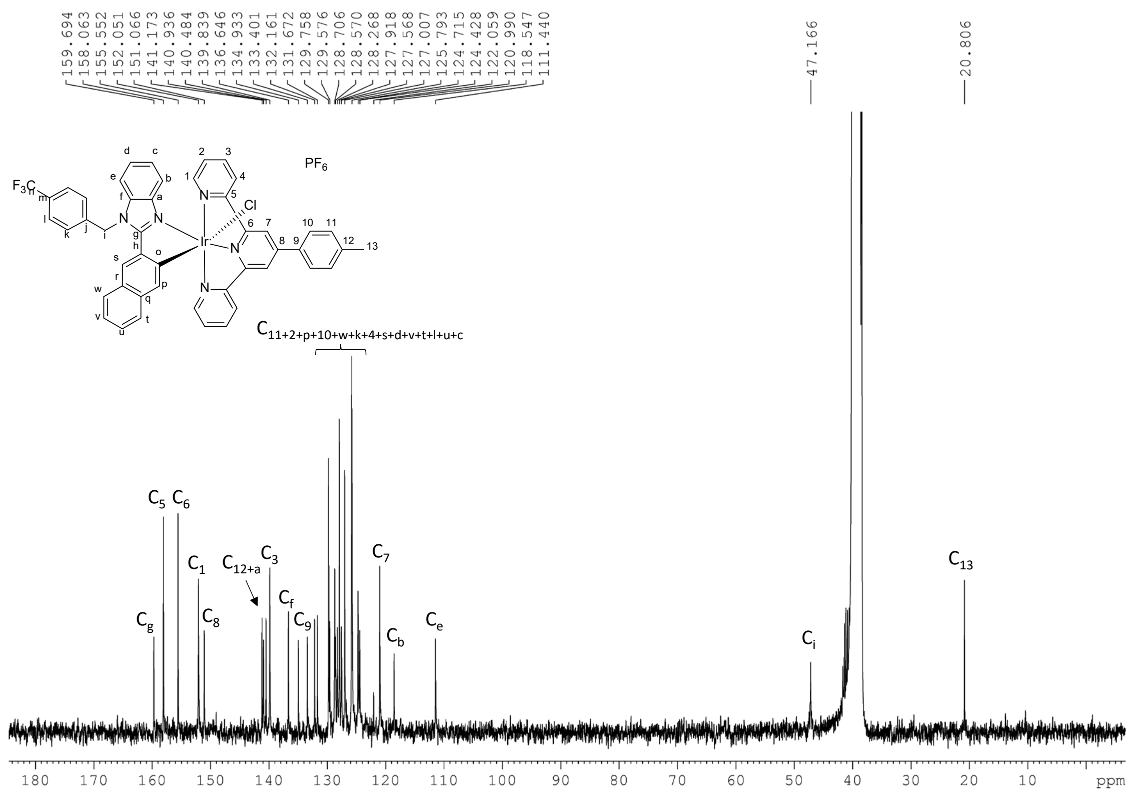

Figure S23. ^{13}C NMR spectrum of **Ir3**, 75 MHz, DMSO-d_6.

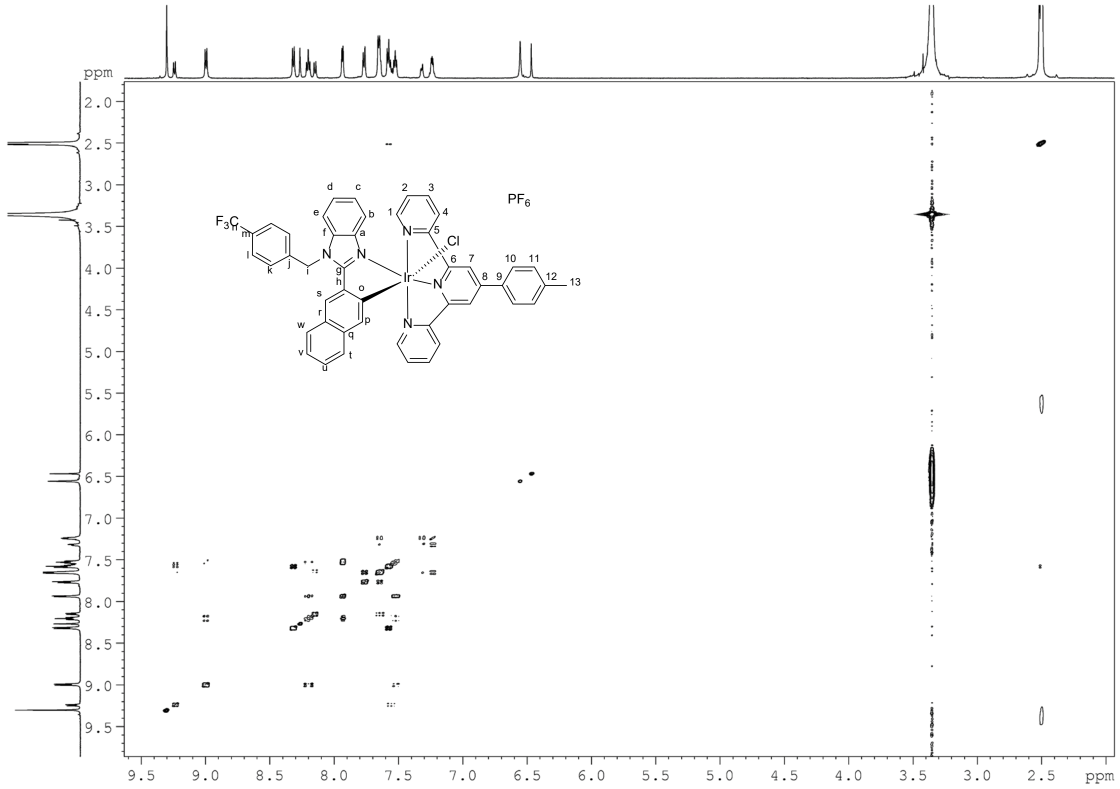

Figure S24. ^1H-^1H COSY NMR spectrum of **Ir3**, 300 MHz, DMSO-d_6.

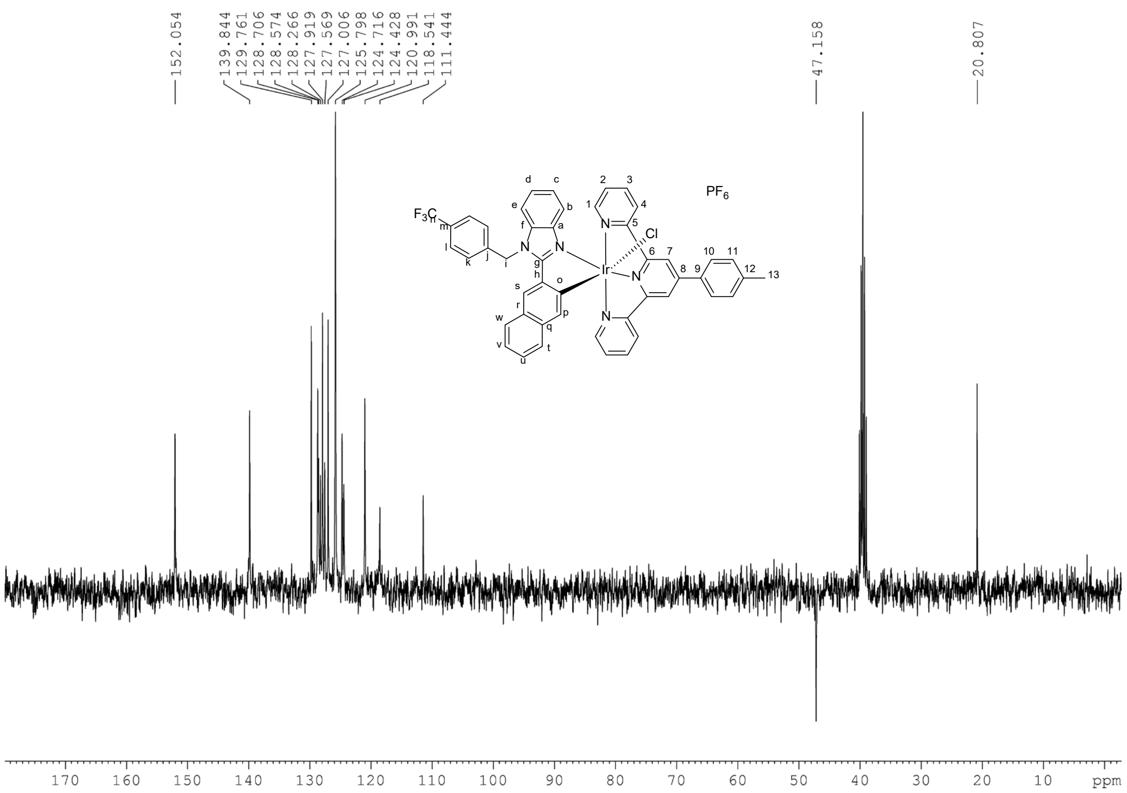

Figure S25. DEPT-135 NMR spectrum of **Ir3**, 75 MHz, DMSO-d_6.

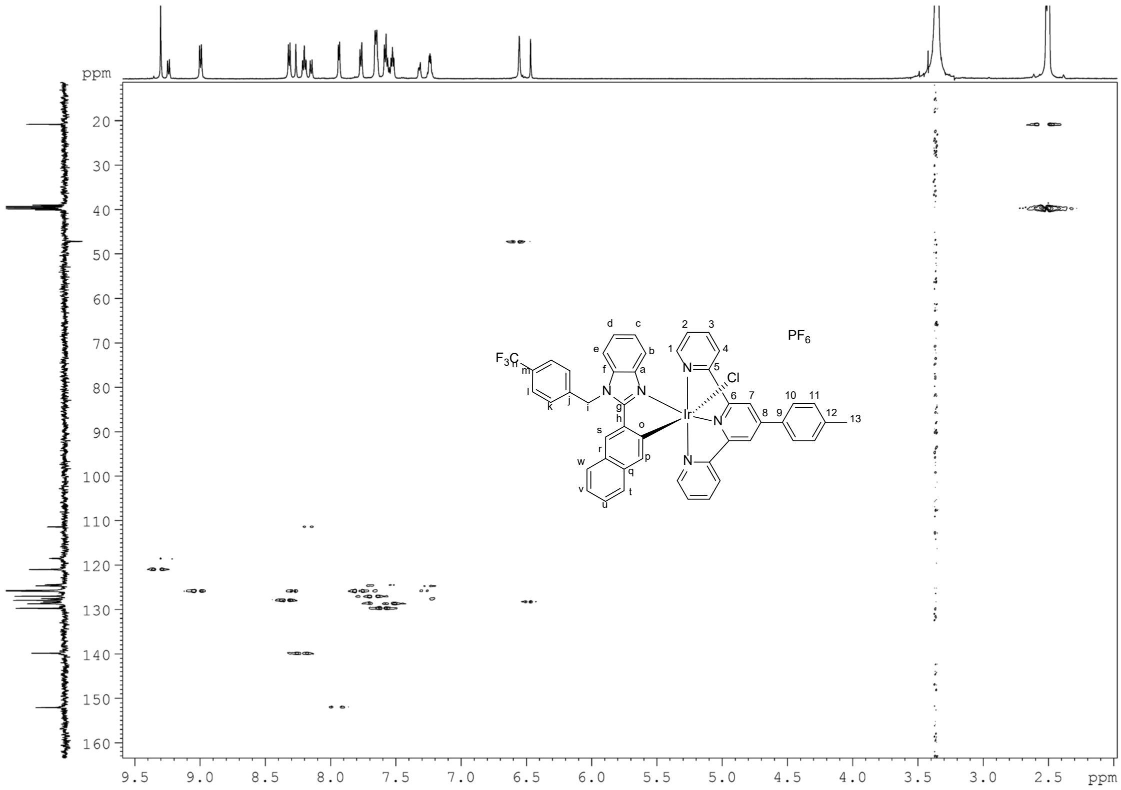

Figure S26. ^{1}H-^{13}C HSQC NMR spectrum of **Ir3**, 300 MHz, DMSO-d_6.

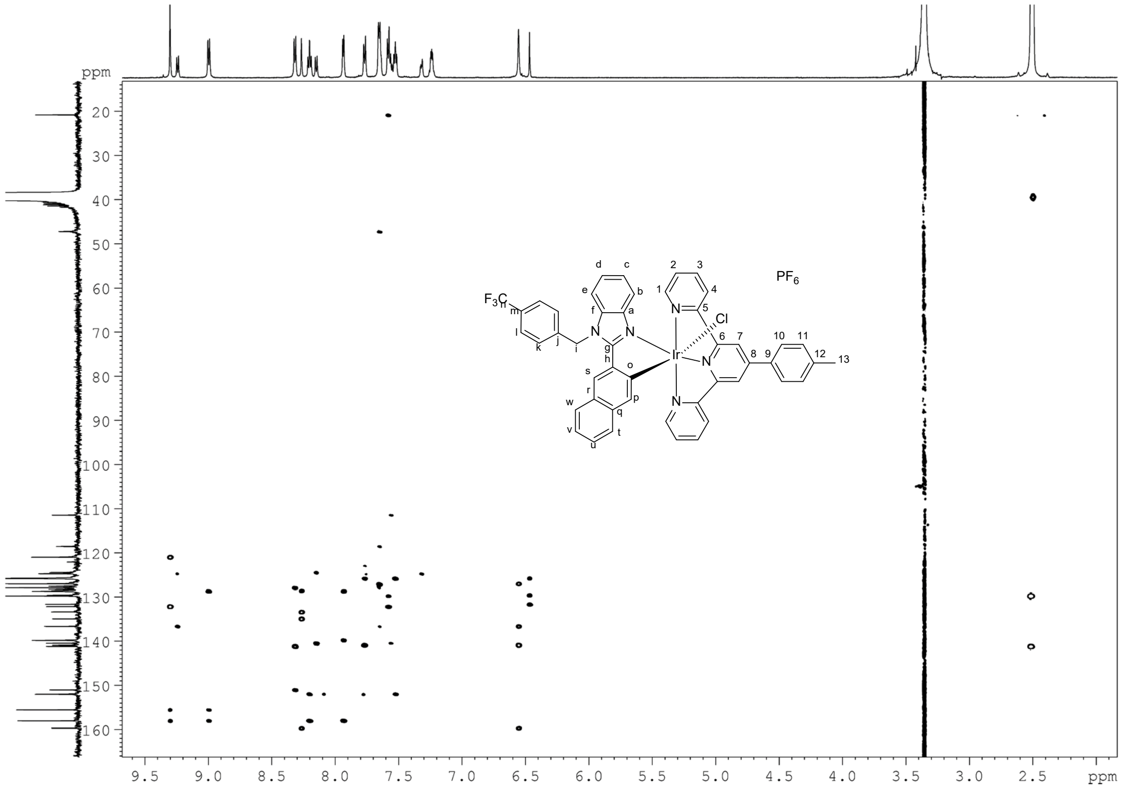

Figure S27. ^{1}H-^{13}C HMBC NMR spectrum of **Ir3**, 300 MHz, DMSO-d_6.

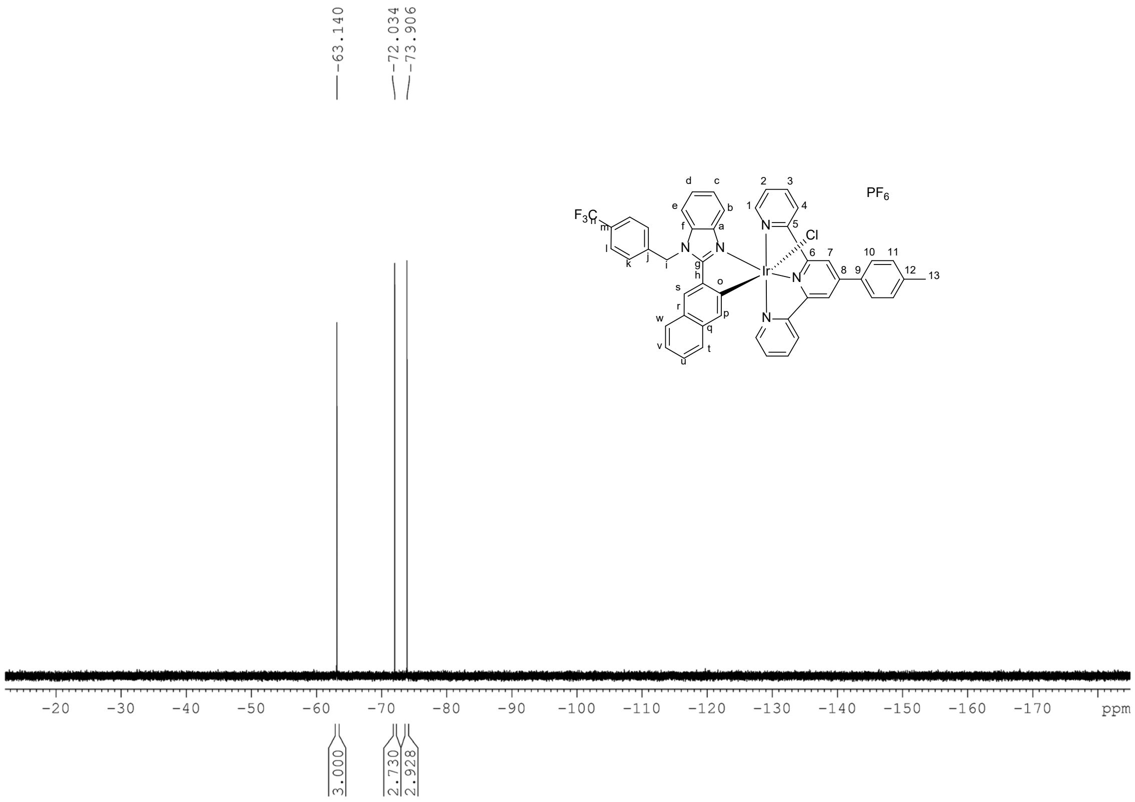

Figure S28. ^{19}F NMR spectrum of **Ir3**, 377 MHz, DMSO-d_6.

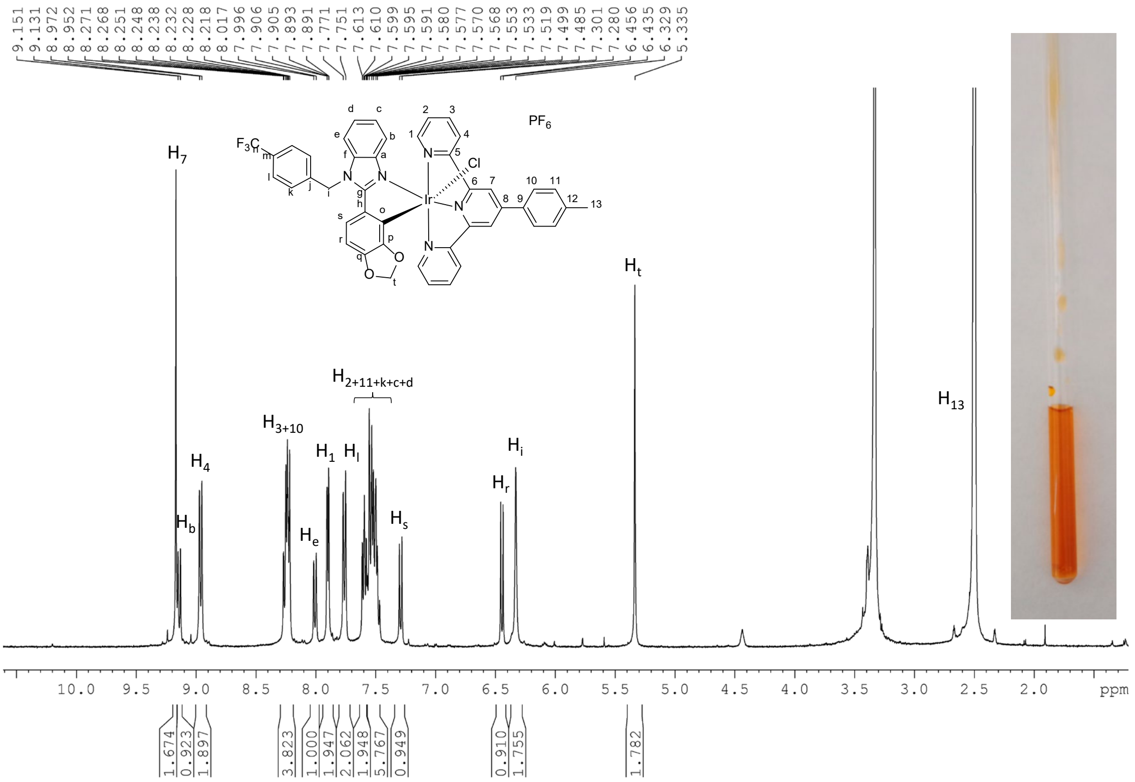

Figure S29. ^{1}H NMR spectrum of **Ir4**, 400 MHz, DMSO-*d*$_6$.

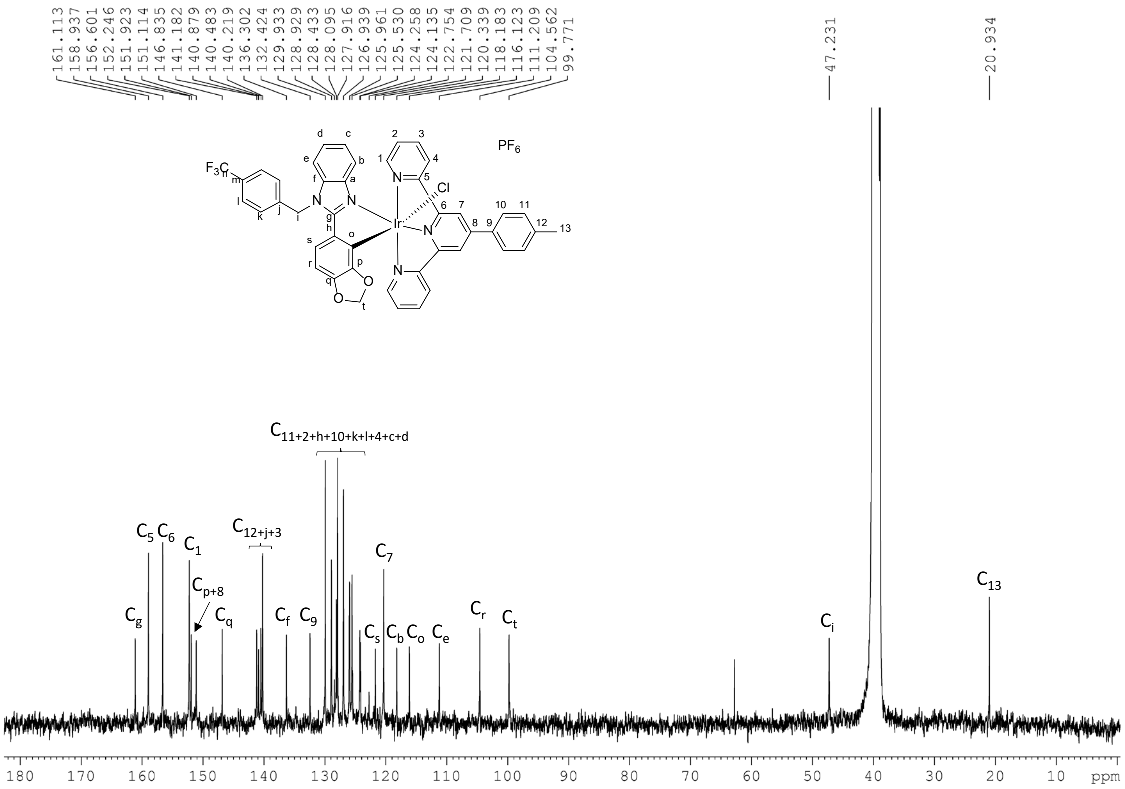

Figure S30. ^{13}C NMR spectrum of **Ir4**, 101 MHz, DMSO-*d*$_6$.

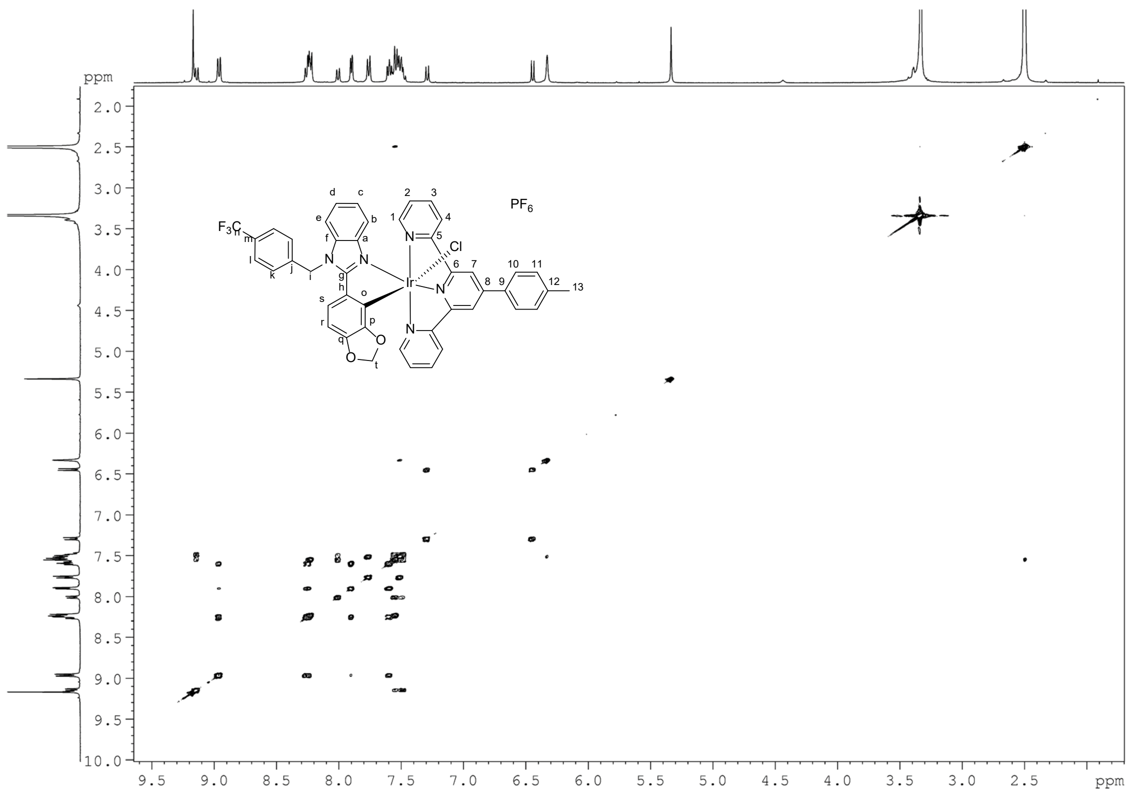

Figure S31. ^1H-^1H COSY NMR spectrum of **Ir4**, 400 MHz, DMSO-d_6.

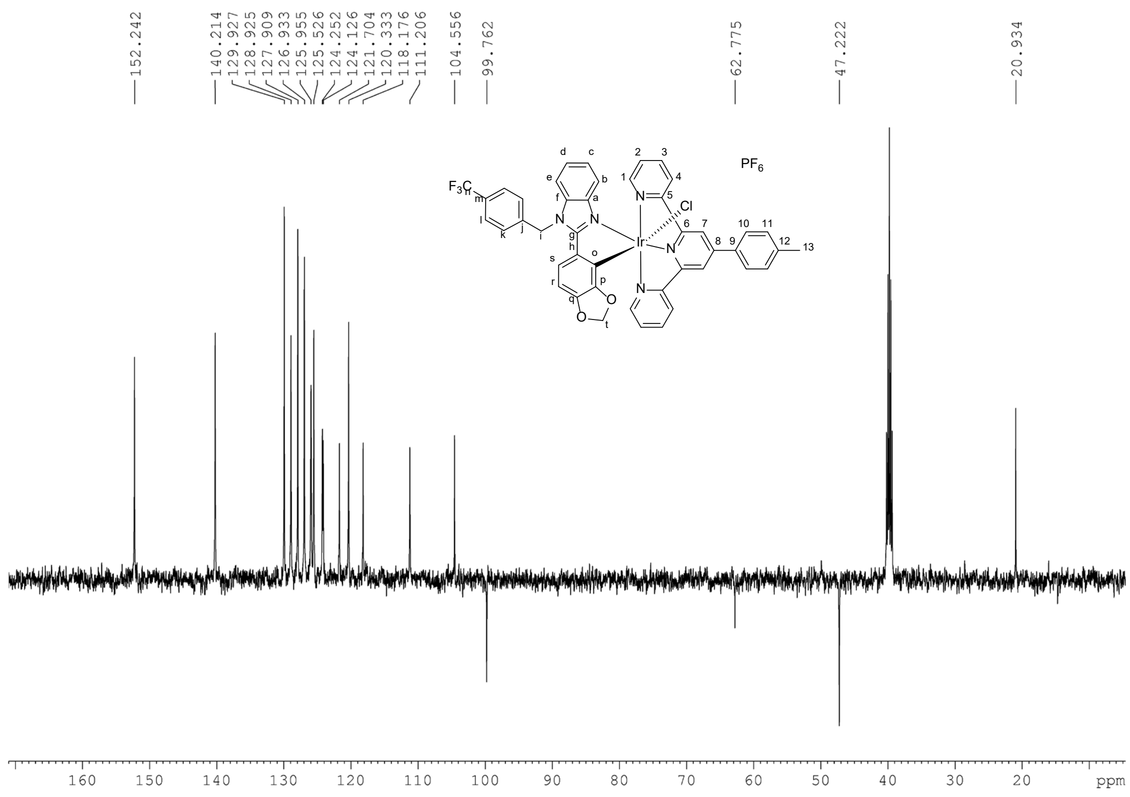

Figure S32. DEPT-135 NMR spectrum of **Ir4**, 101 MHz, DMSO-d_6.

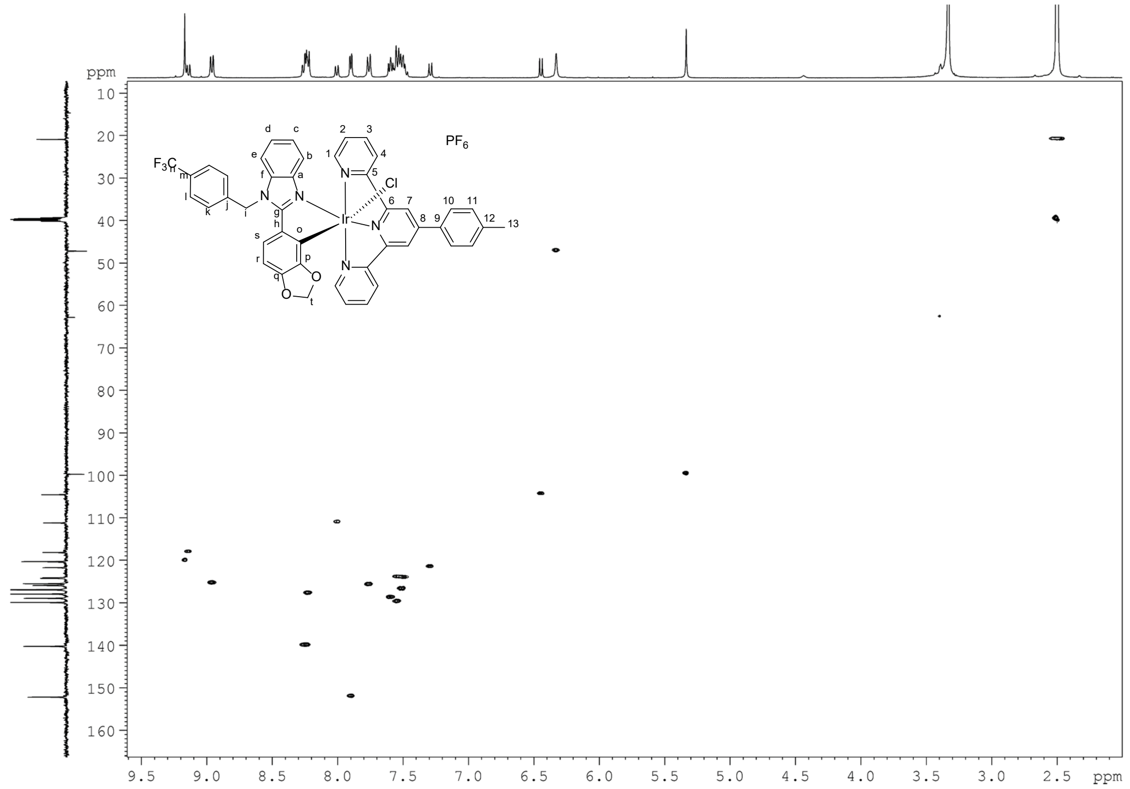

Figure S33. ^{1}H-^{13}C HSQC NMR spectrum of **Ir4**, 400 MHz, DMSO-d_6.

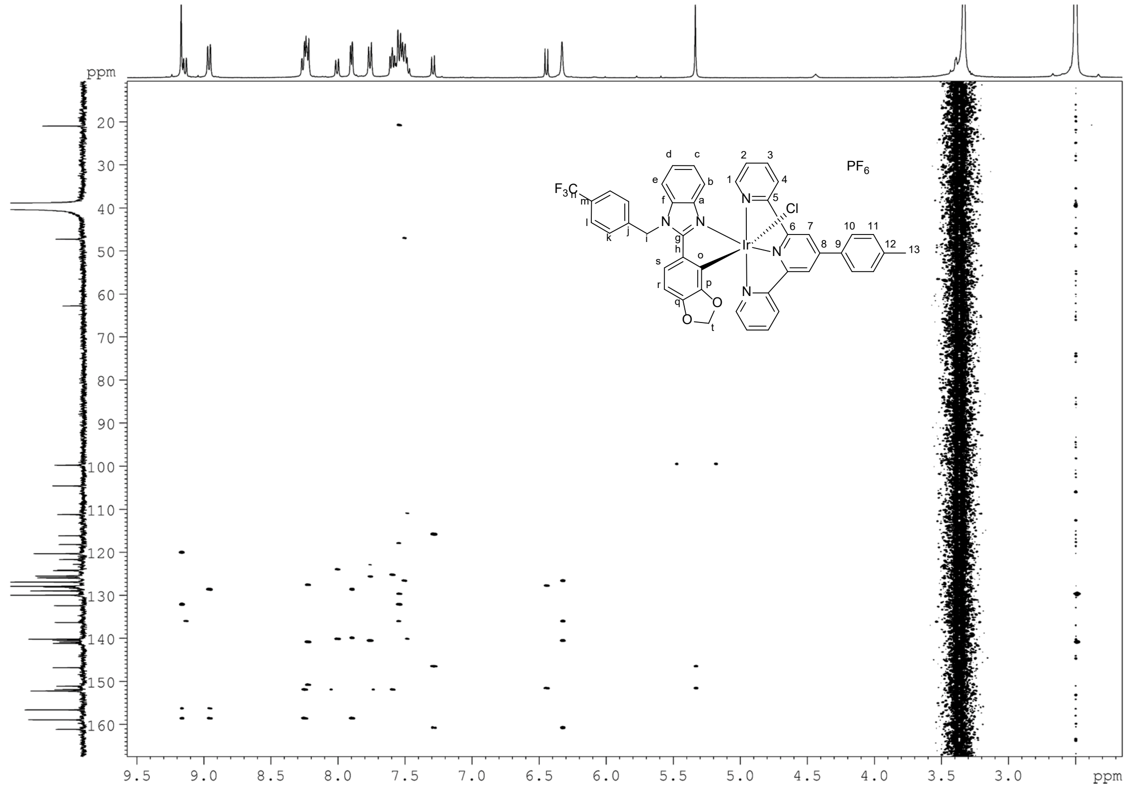

Figure S34. ^{1}H-^{13}C HMBC NMR spectrum of **Ir4**, 400 MHz, DMSO-d_6.

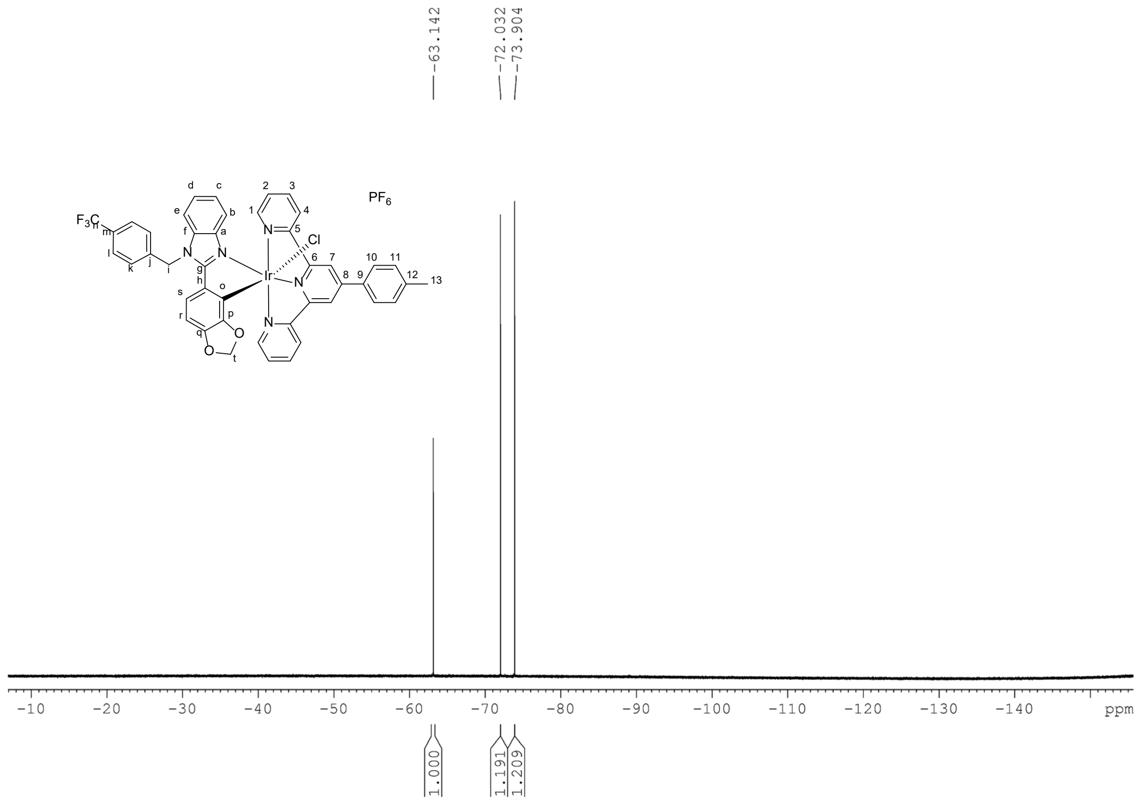

Figure S35. ^{19}F NMR spectrum of **Ir4**, 377 MHz, DMSO-d_6.

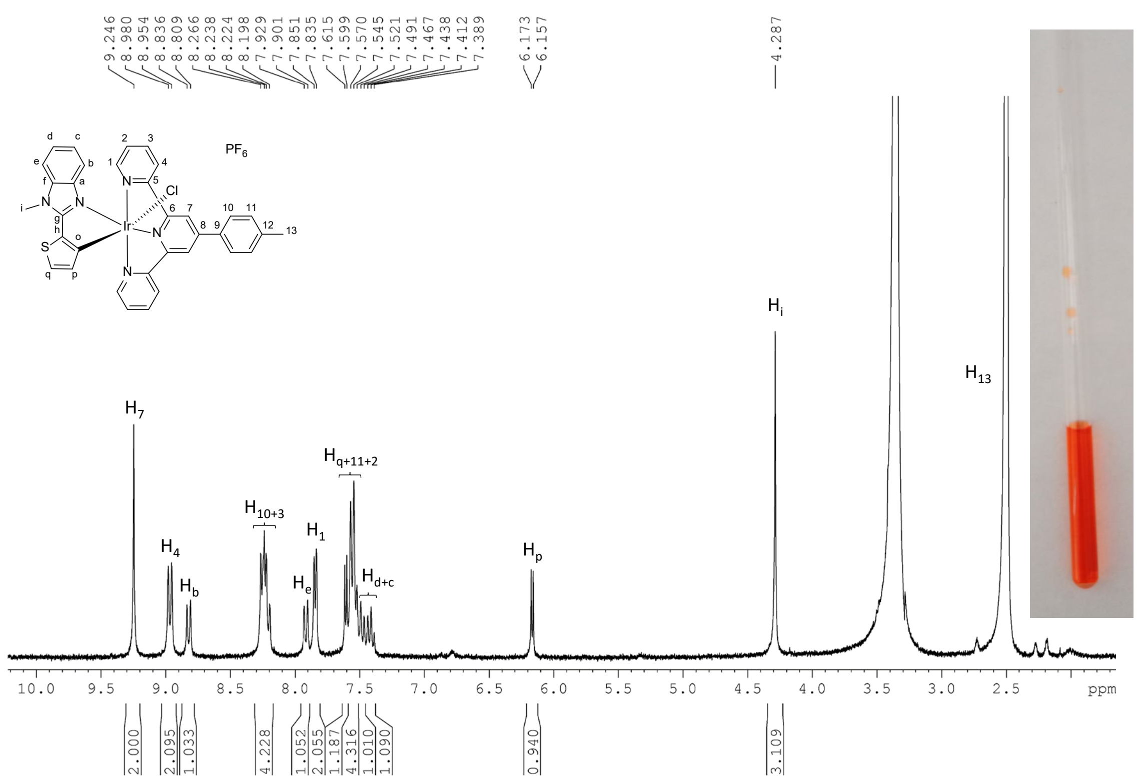

Figure S36. ^{1}H NMR spectrum of **Ir5**, 600 MHz, DMSO-d_6.

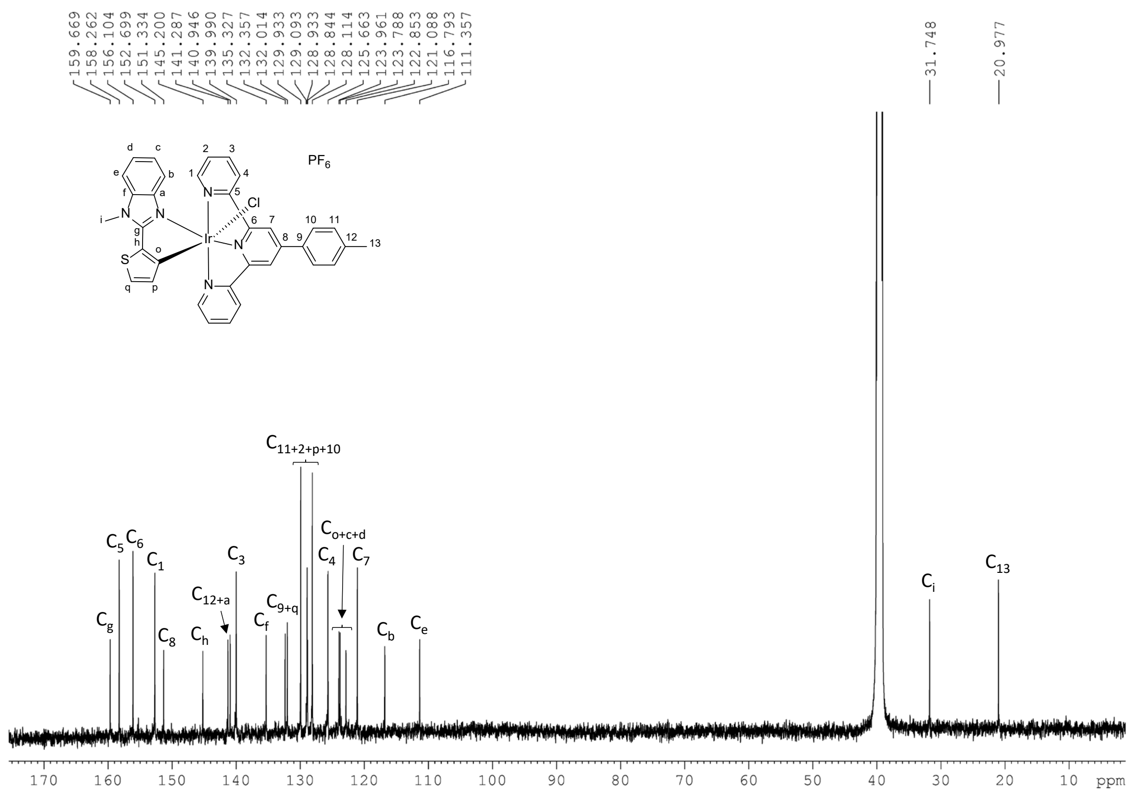

Figure S37. ^{13}C NMR spectrum of **Ir5**, 151 MHz, DMSO-d_6.

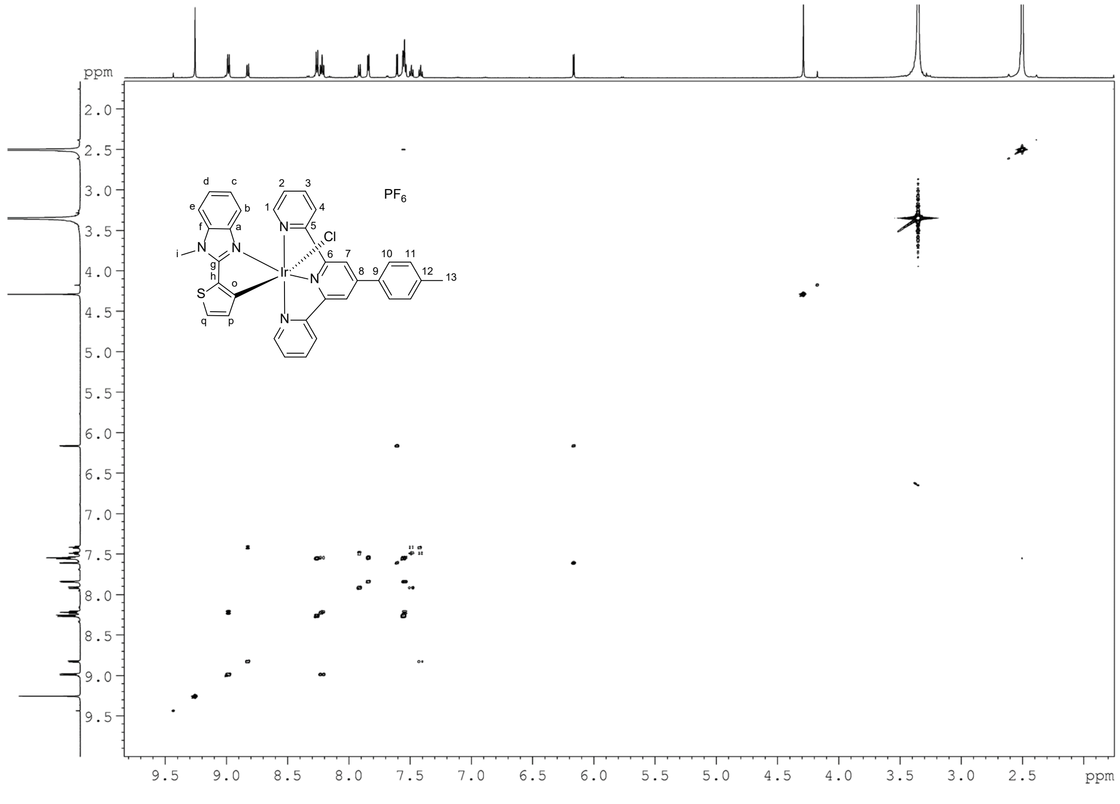

Figure S38. ^{1}H-^{1}H COSY NMR spectrum of **Ir5**, 600 MHz, DMSO-d_6.

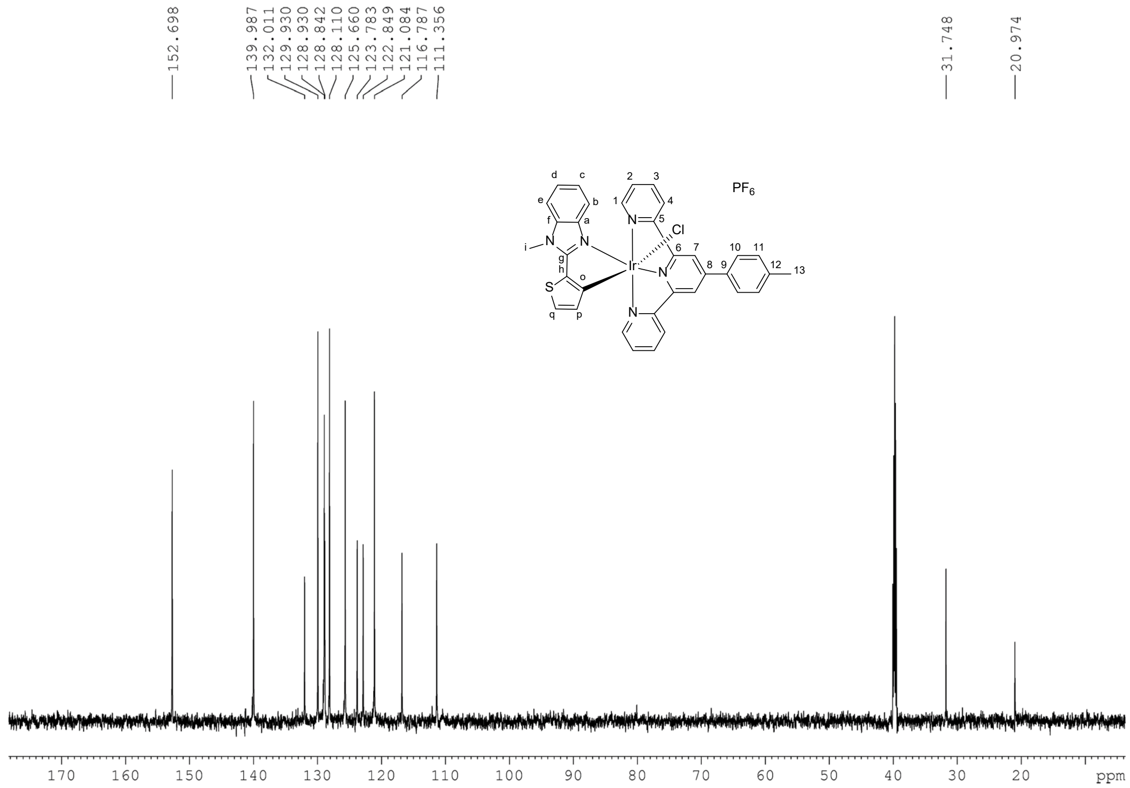

Figure S39. DEPT-135 NMR spectrum of **Ir5**, 151 MHz, DMSO-d_6.

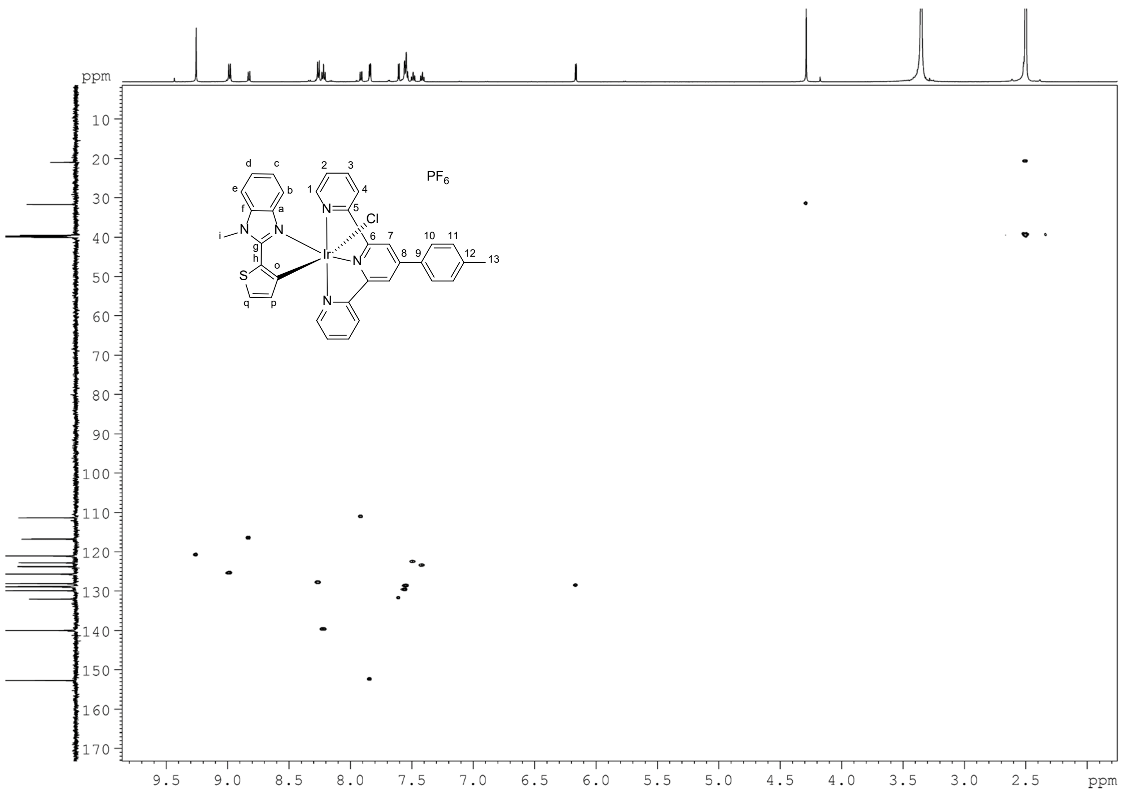

Figure S40. ^{1}H-^{13}C HSQC NMR spectrum of **Ir5**, 600 MHz, DMSO-d_6.

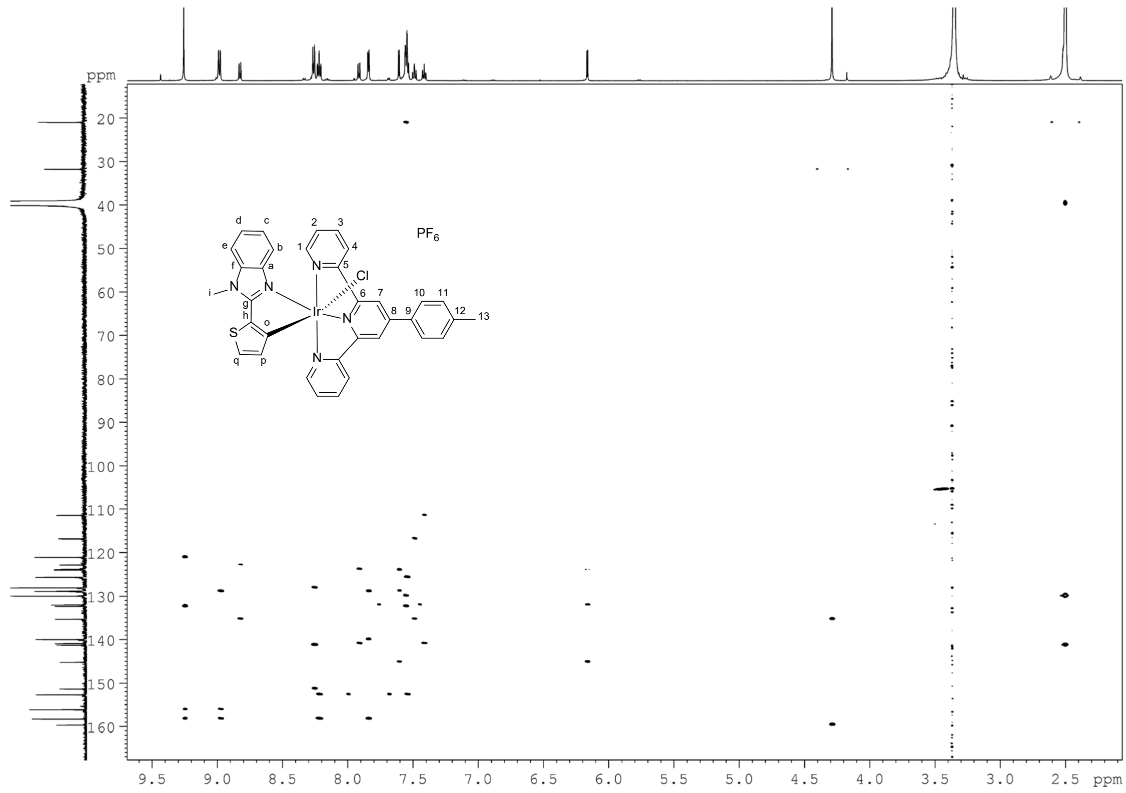

Figure S41. ^{1}H-^{13}C HMBC NMR spectrum of **Ir5**, 151 MHz, DMSO-*d*$_6$.

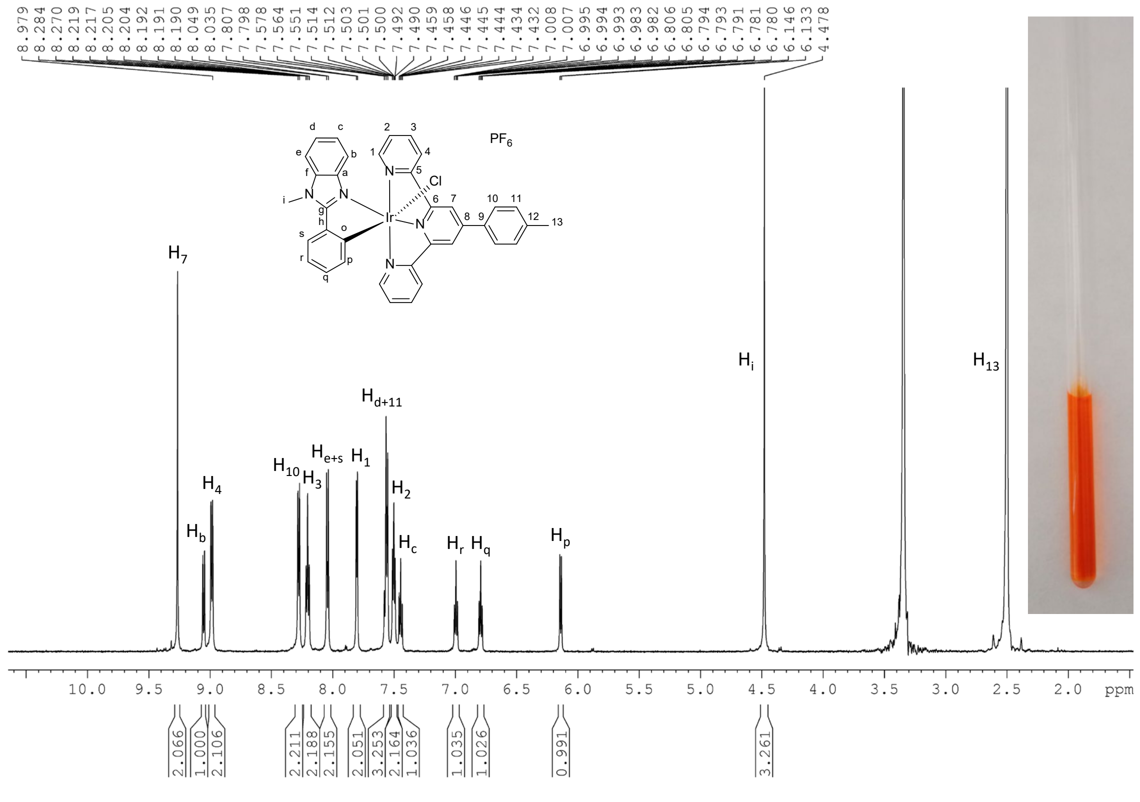

Figure S42. ^{1}H NMR spectrum of **Ir6**, 600 MHz, DMSO-*d*$_6$.

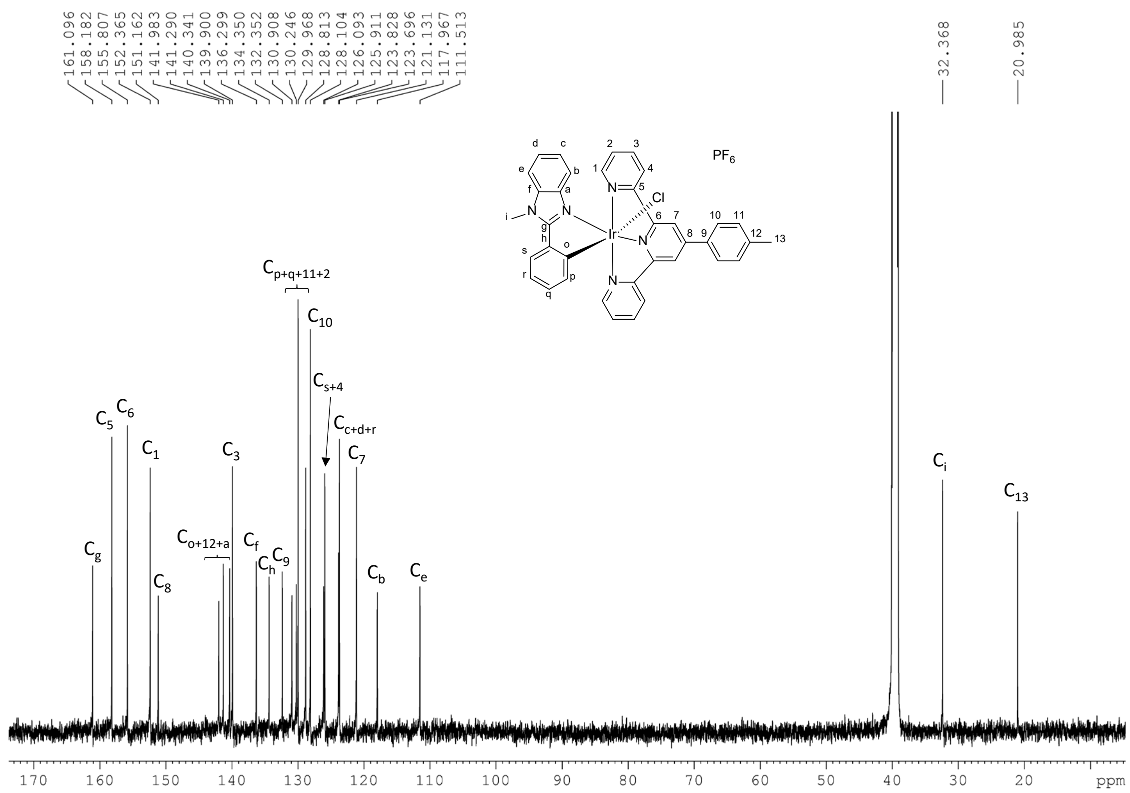

Figure S43. ^{13}C NMR spectrum of **Ir6**, 151 MHz, DMSO-d_6.

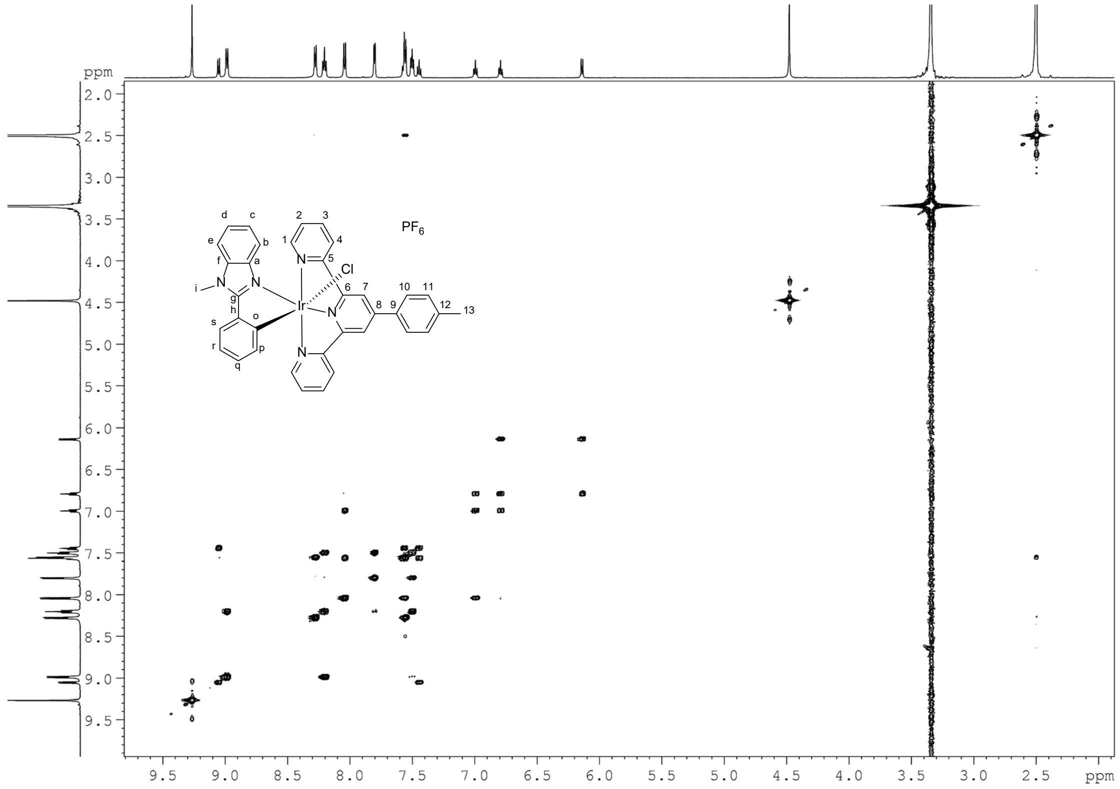

Figure S44. ^1H-^1H COSY NMR spectrum of **Ir6**, 600 MHz, DMSO-d_6.

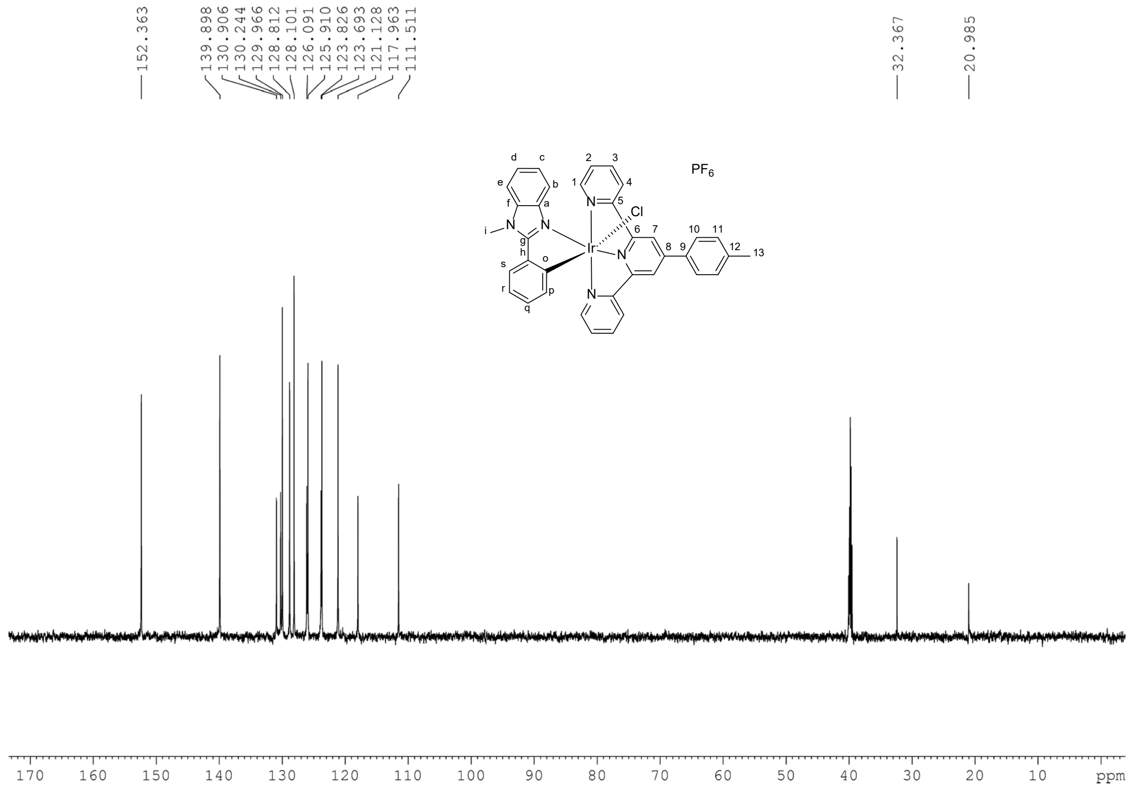

Figure S45. DEPT-135 NMR spectrum of **Ir6**, 151 MHz, DMSO-d_6.

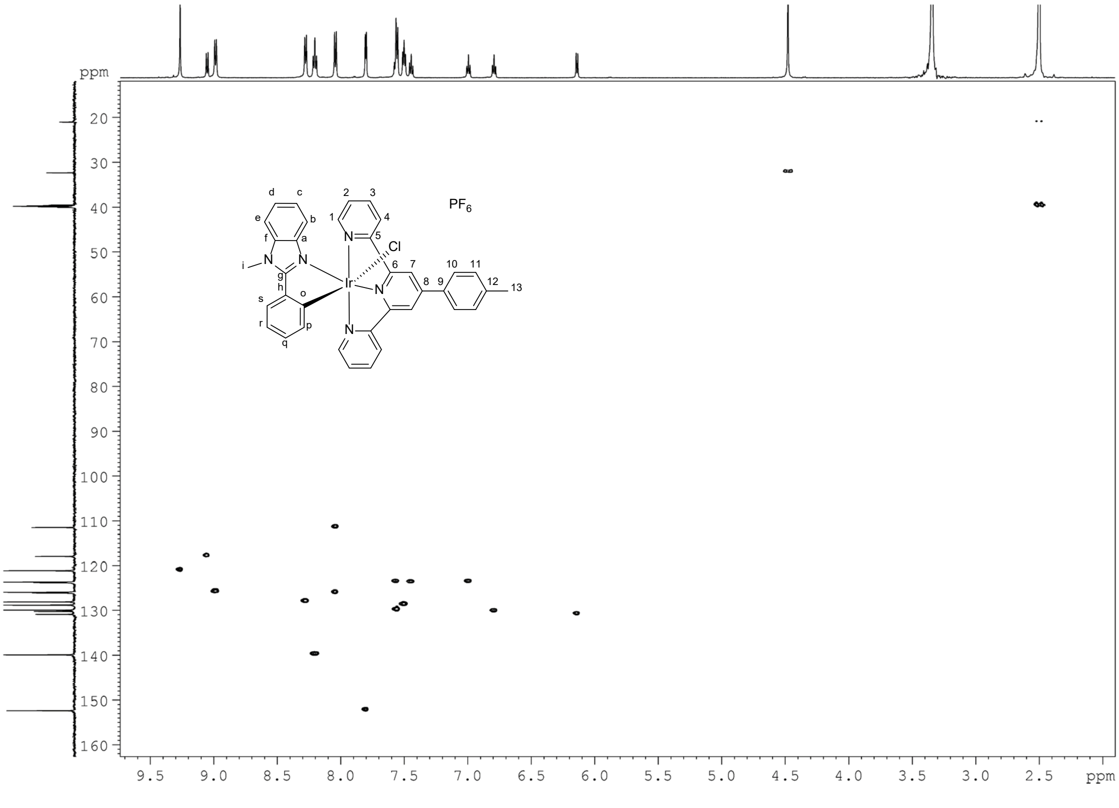

Figure S46. ^{1}H-^{13}C HSQC NMR spectrum of **Ir6**, 600 MHz, DMSO-d_6.

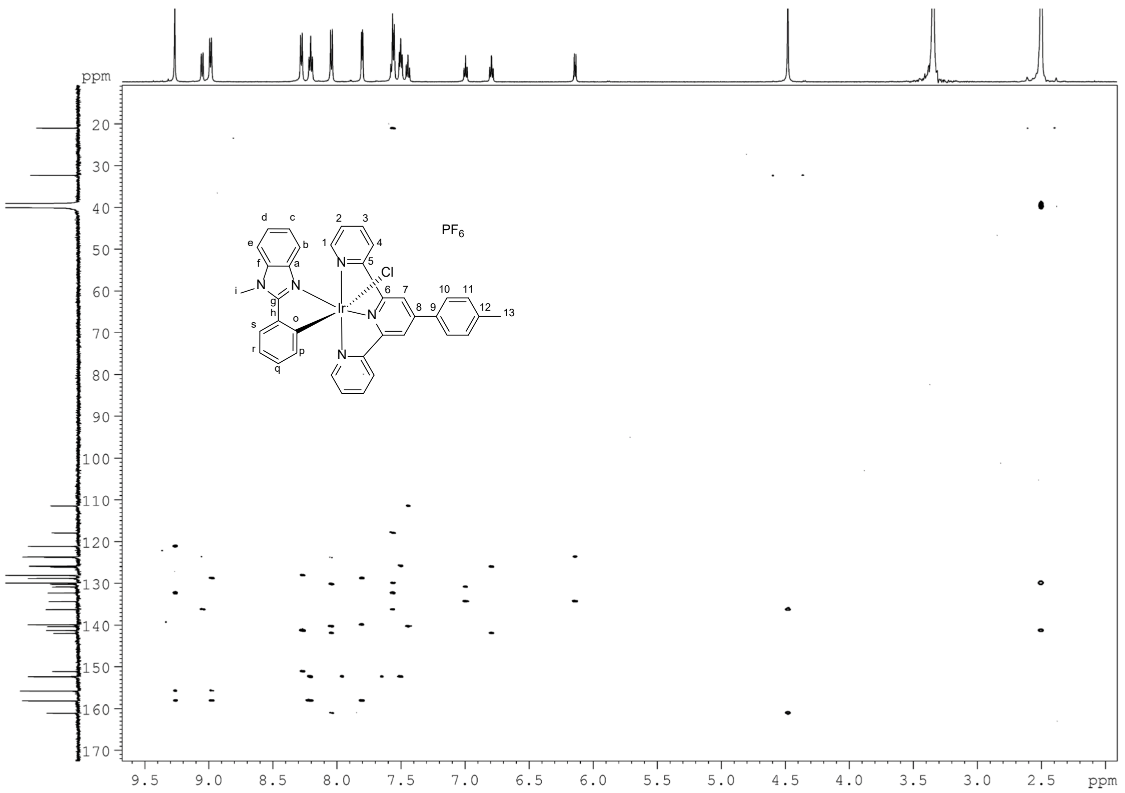

Figure S47. ^{1}H-^{13}C HMBC NMR spectrum of **Ir6**, 600 MHz, DMSO-d_6.

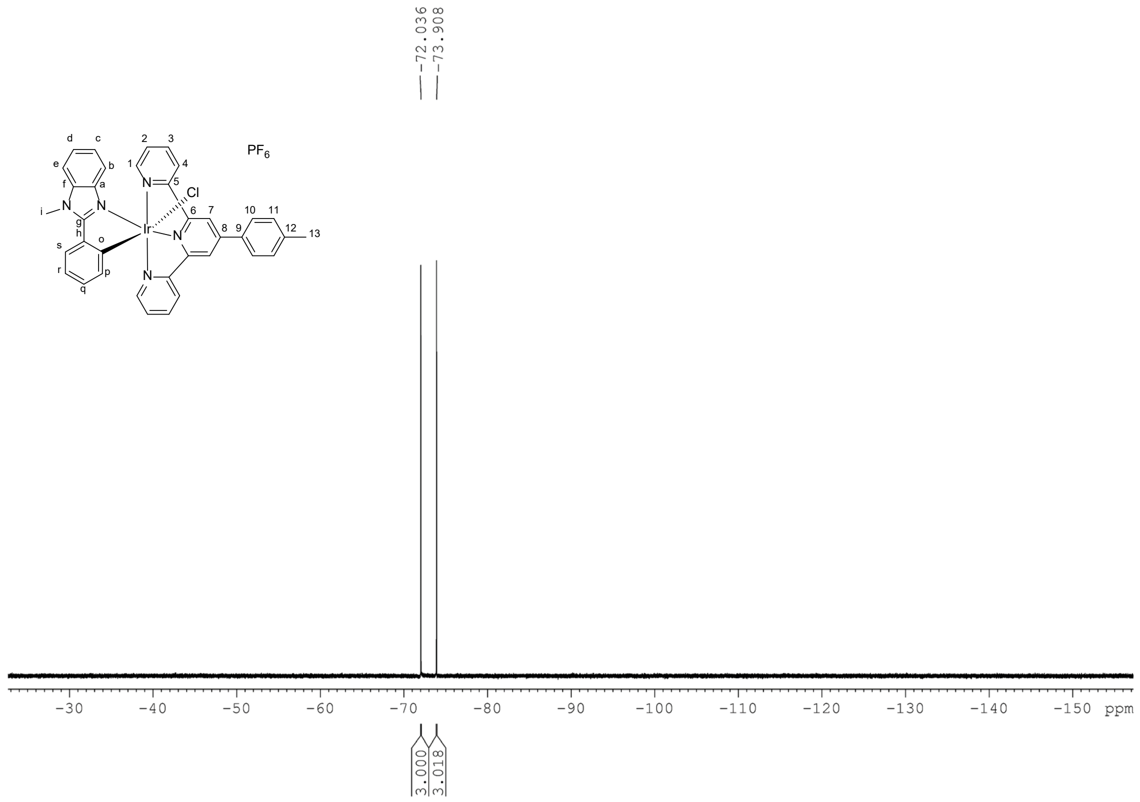

Figure S48. ^{19}F NMR spectrum of **Ir6**, 377 MHz, DMSO-d_6.

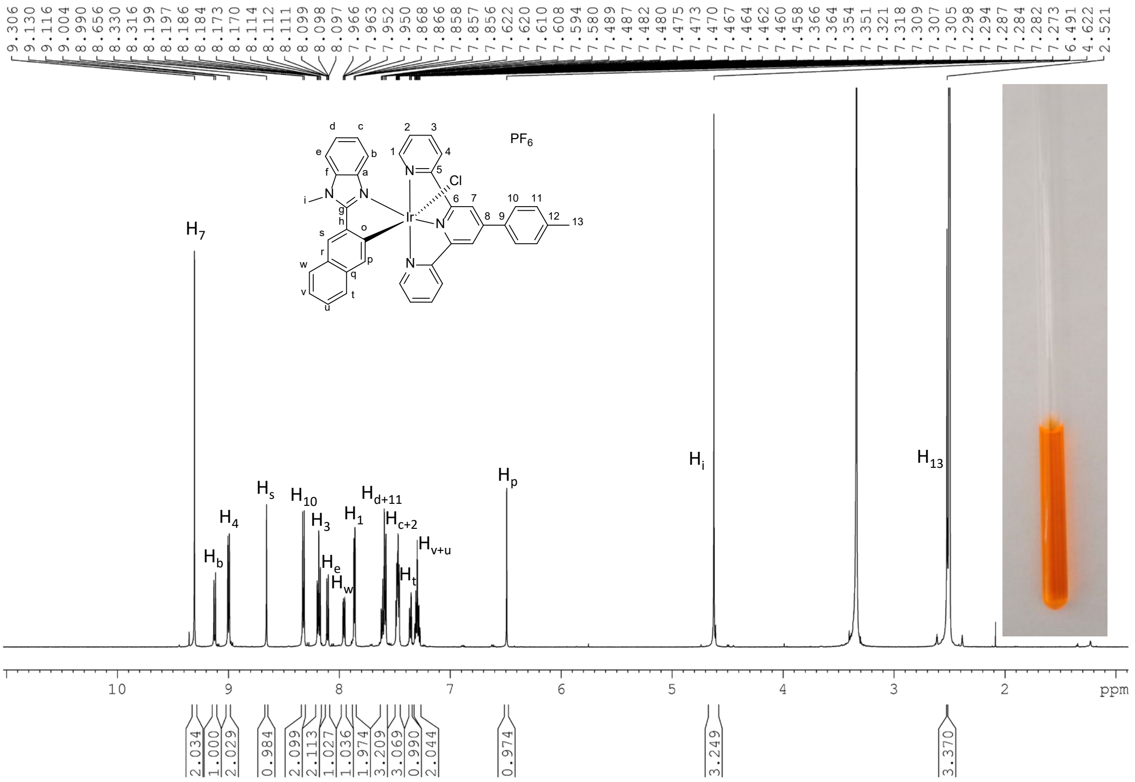

Figure S49. ^{1}H NMR spectrum of **Ir7**, 600 MHz, DMSO-d_6.

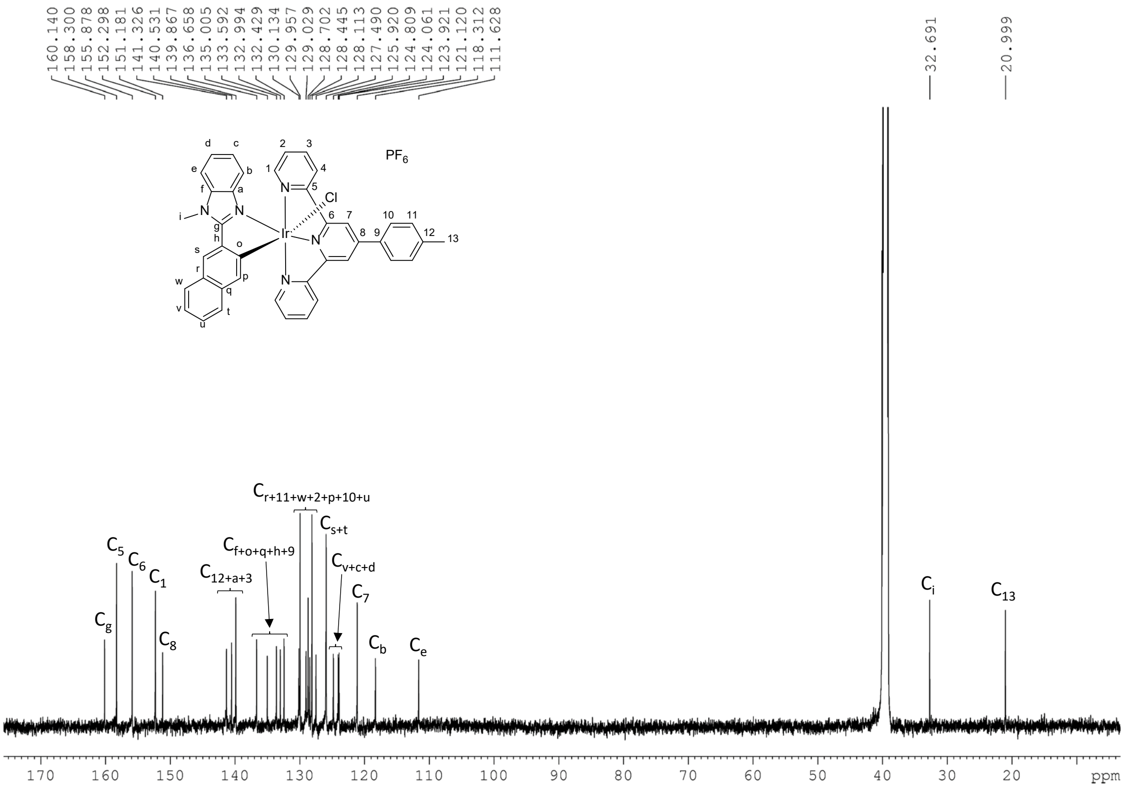

Figure S50. ^{13}C NMR spectrum of **Ir7**, 151 MHz, DMSO-d_6.

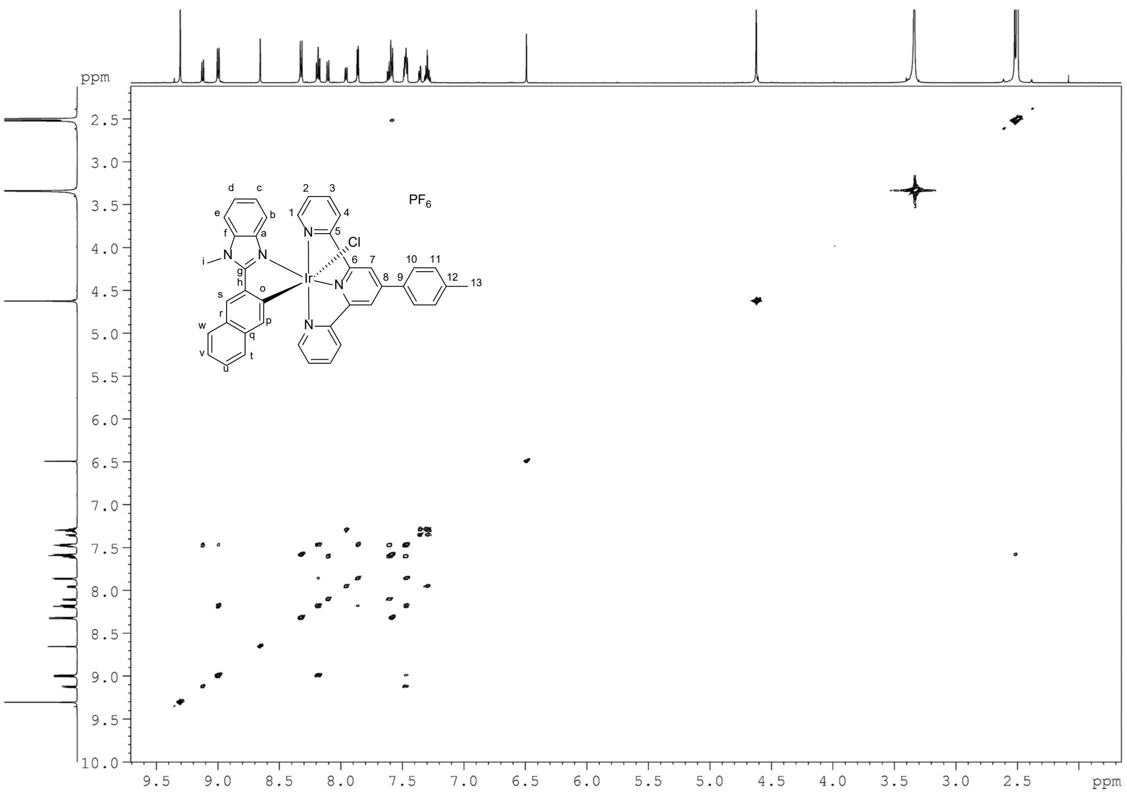

Figure S51. ^{1}H-^{1}H COSY NMR spectrum of **Ir7**, 600 MHz, DMSO-d_6.

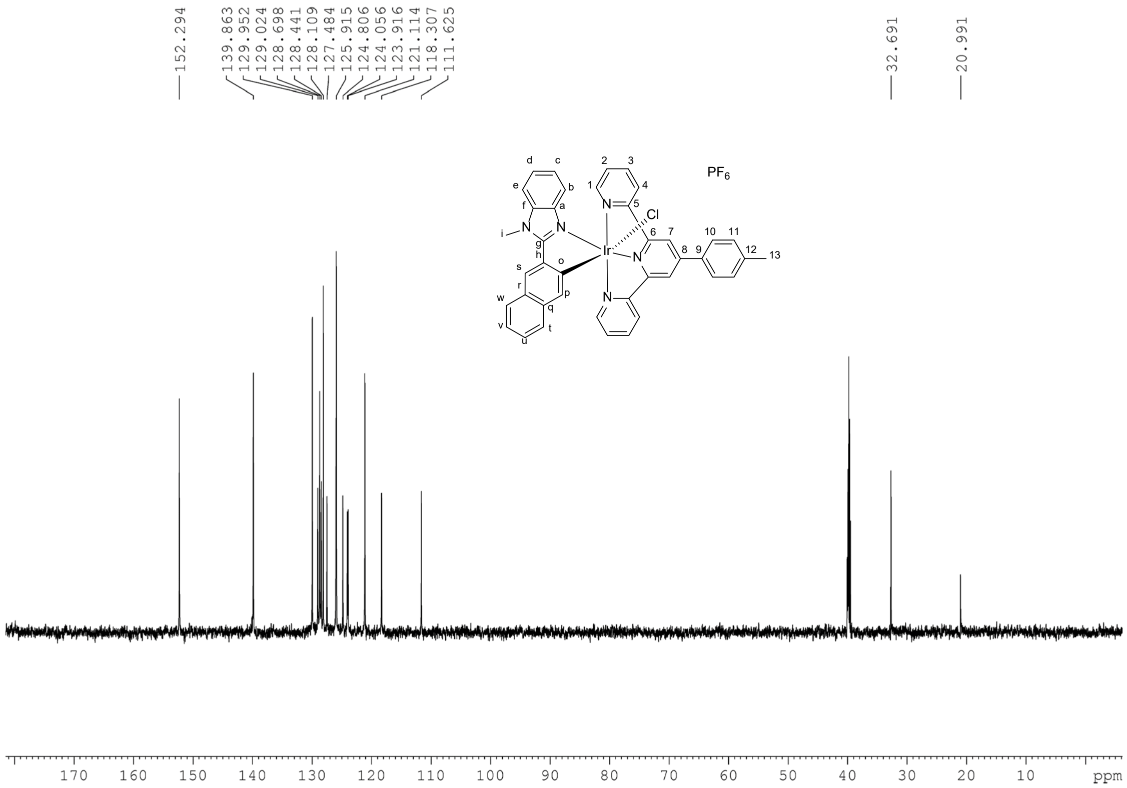

Figure S52. DEPT-135 NMR spectrum of **Ir7**, 151 MHz, DMSO-d_6.

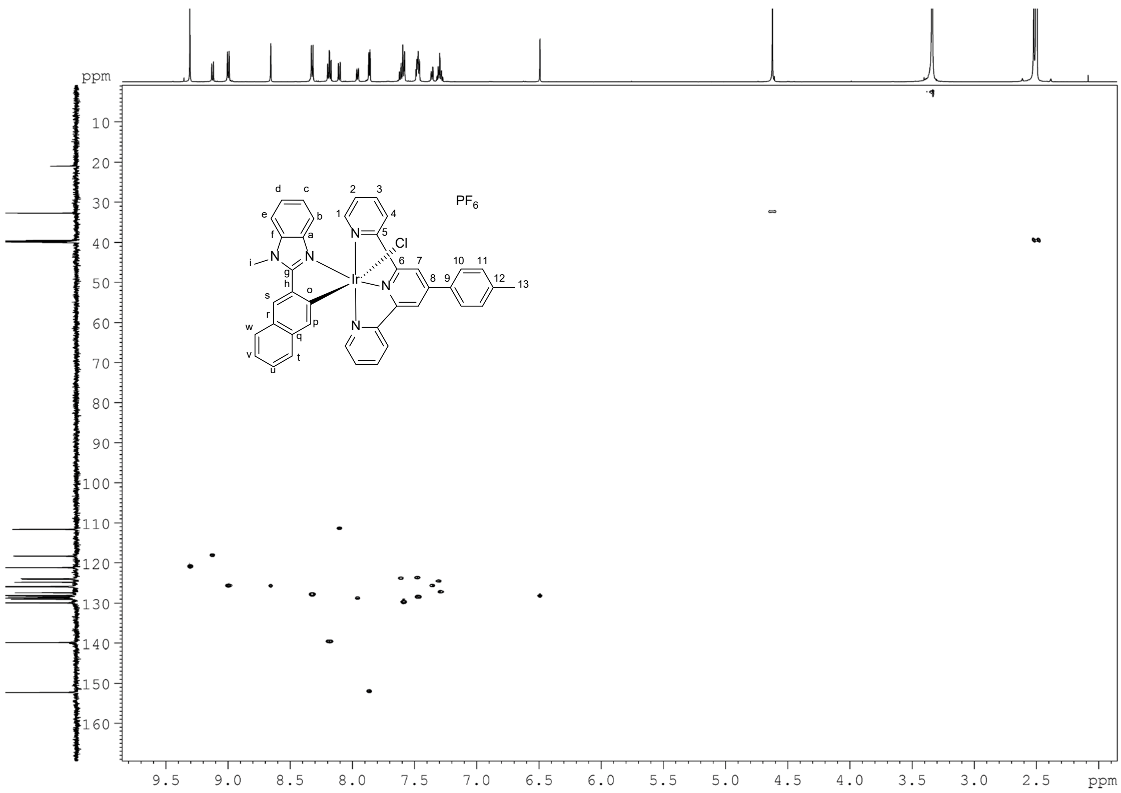

Figure S53. ^{1}H-^{13}C HSQC NMR spectrum of **Ir7**, 600 MHz, DMSO-d_6.

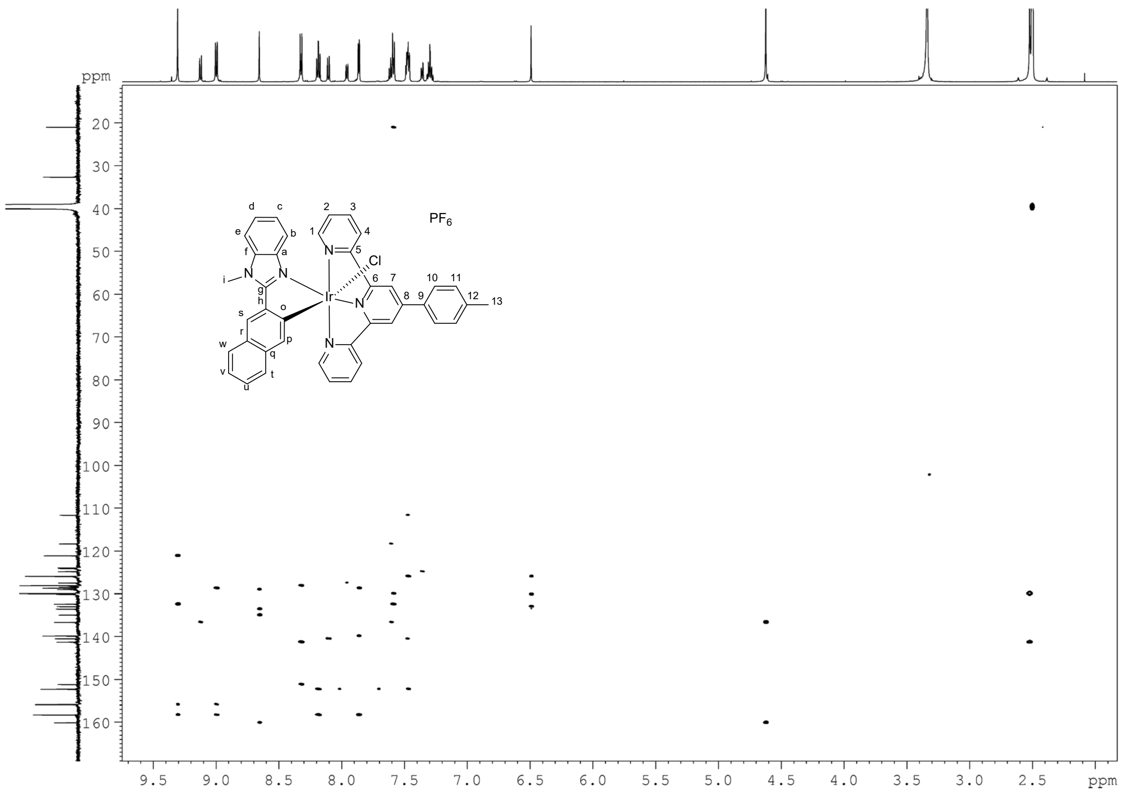

Figure S54. ^{1}H-^{13}C HMBC NMR spectrum of **Ir7**, 600 MHz, DMSO-d_6.

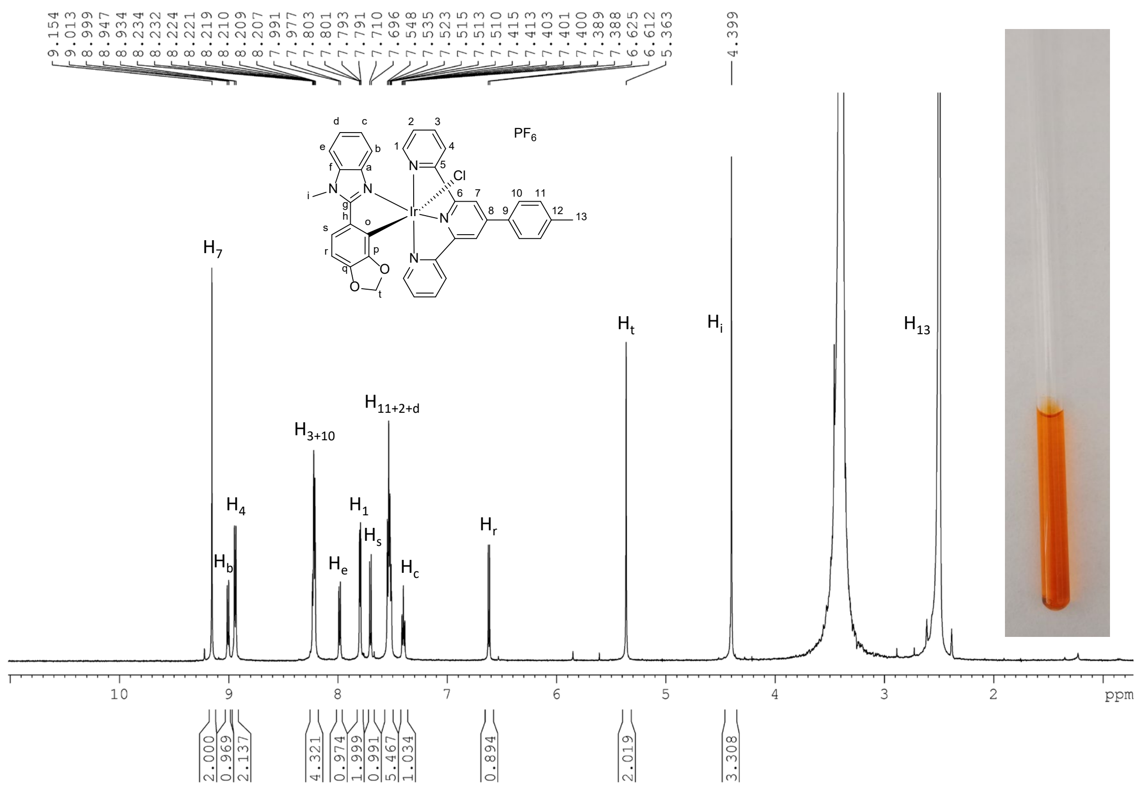

Figure S55. ^{1}H NMR spectrum of **Ir8**, 600 MHz, DMSO-d_6.

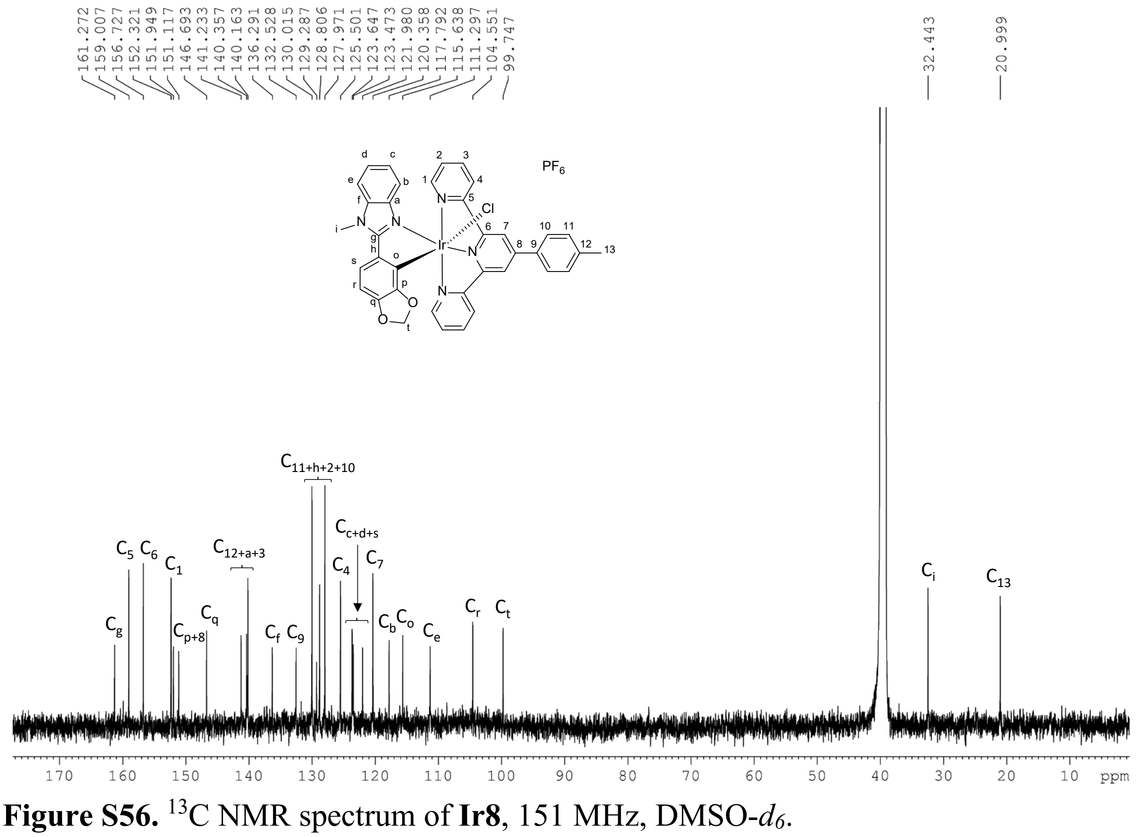

Figure S56. ^{13}C NMR spectrum of **Ir8**, 151 MHz, DMSO-d_6.

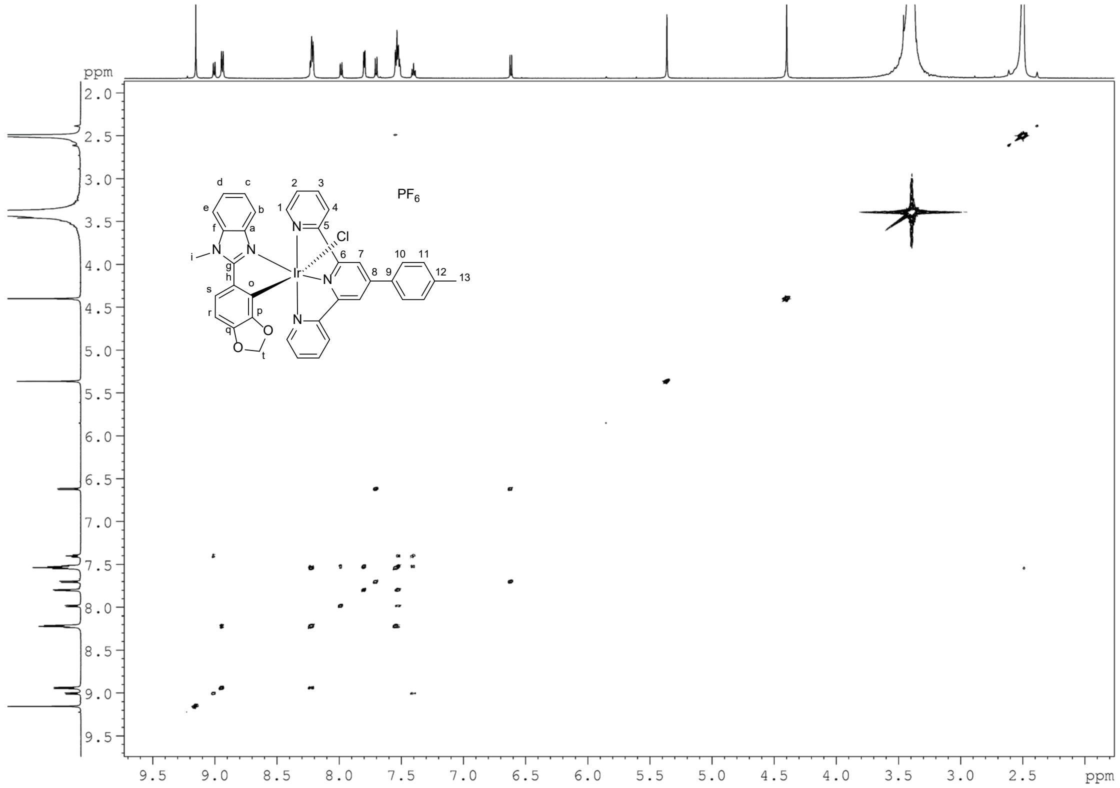

Figure S57. ^{1}H-^{1}H COSY NMR spectrum of **Ir8**, 600 MHz, DMSO-d_6.

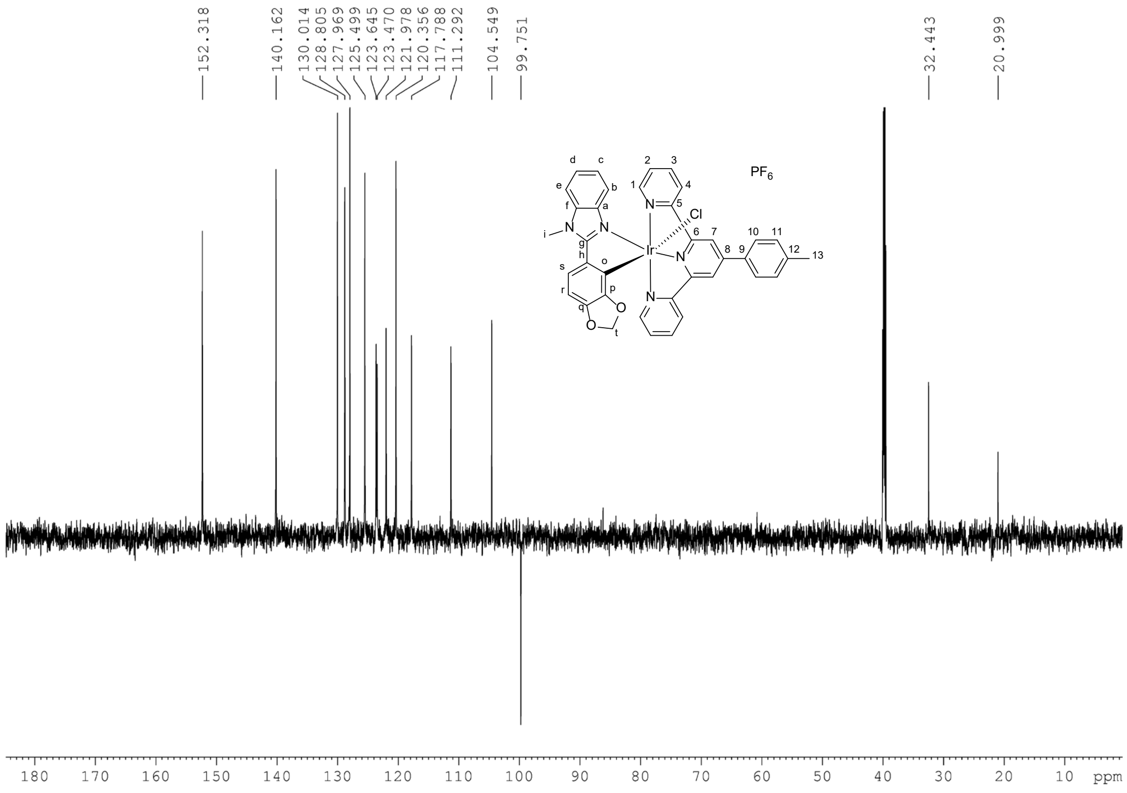

Figure S58. DEPT-135 NMR spectrum of **Ir8**, 151 MHz, DMSO-d_6.

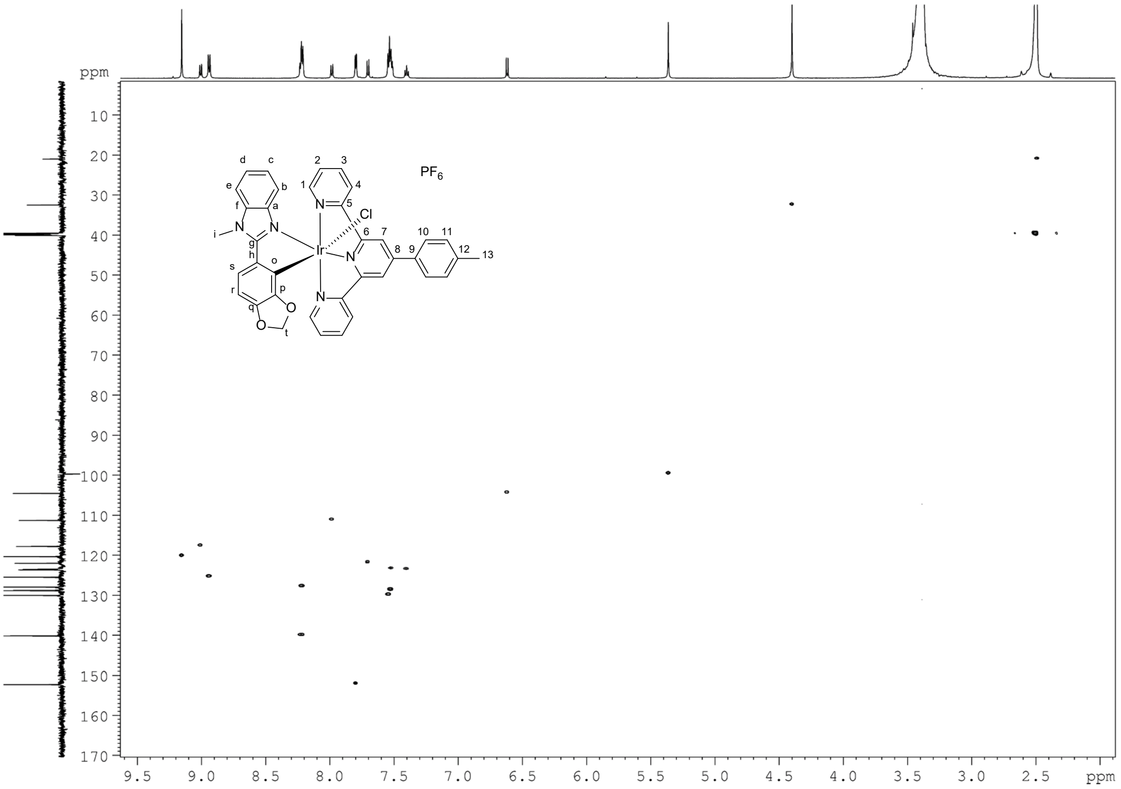

Figure S59. ^{1}H-^{13}C HSQC NMR spectrum of **Ir8**, 600 MHz, DMSO-*d*$_6$.

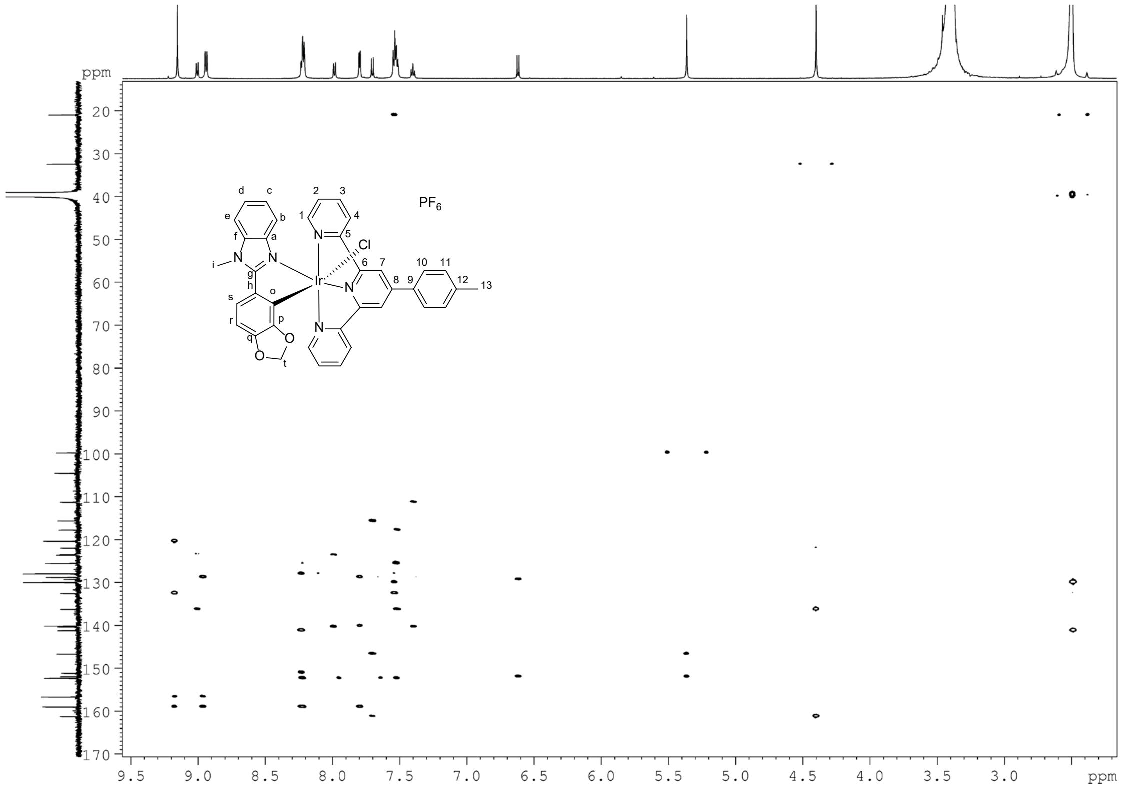

Figure S60. ^{1}H-^{13}C HMBC NMR spectrum of **Ir8**, 600 MHz, DMSO-*d*$_6$.

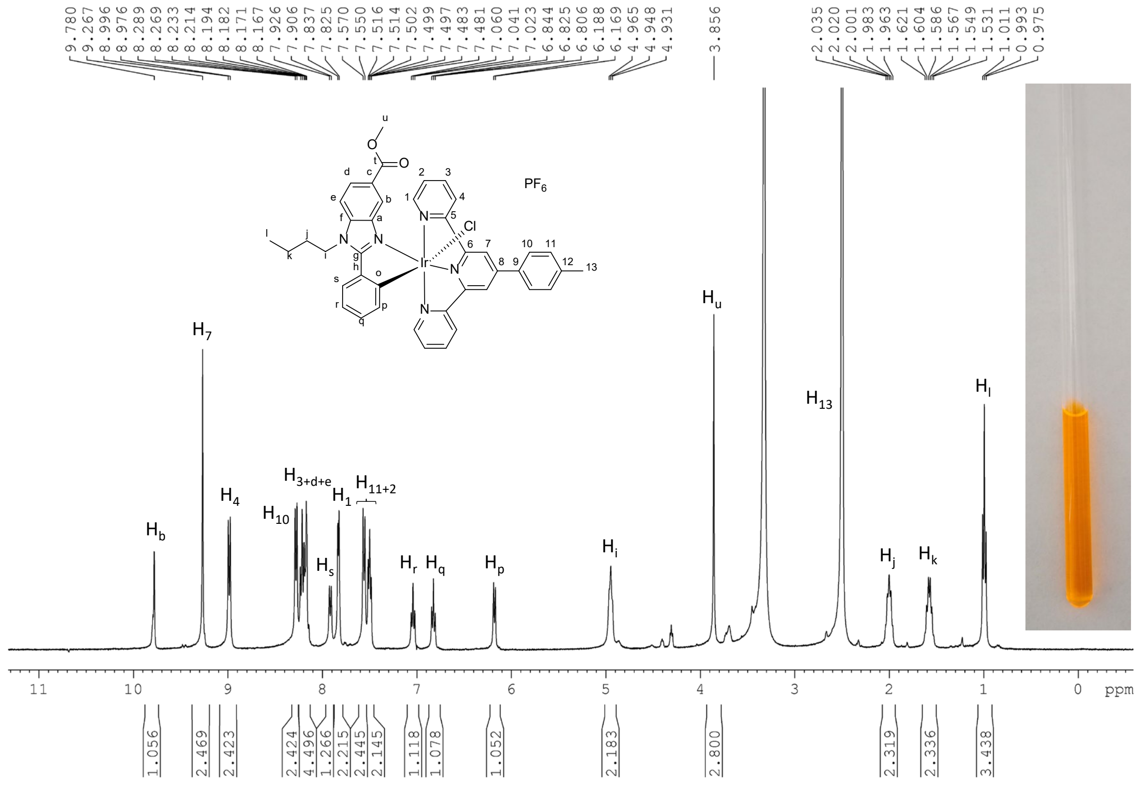

Figure S61. ^{1}H NMR spectrum of **Ir9**, 400 MHz, DMSO-d_6.

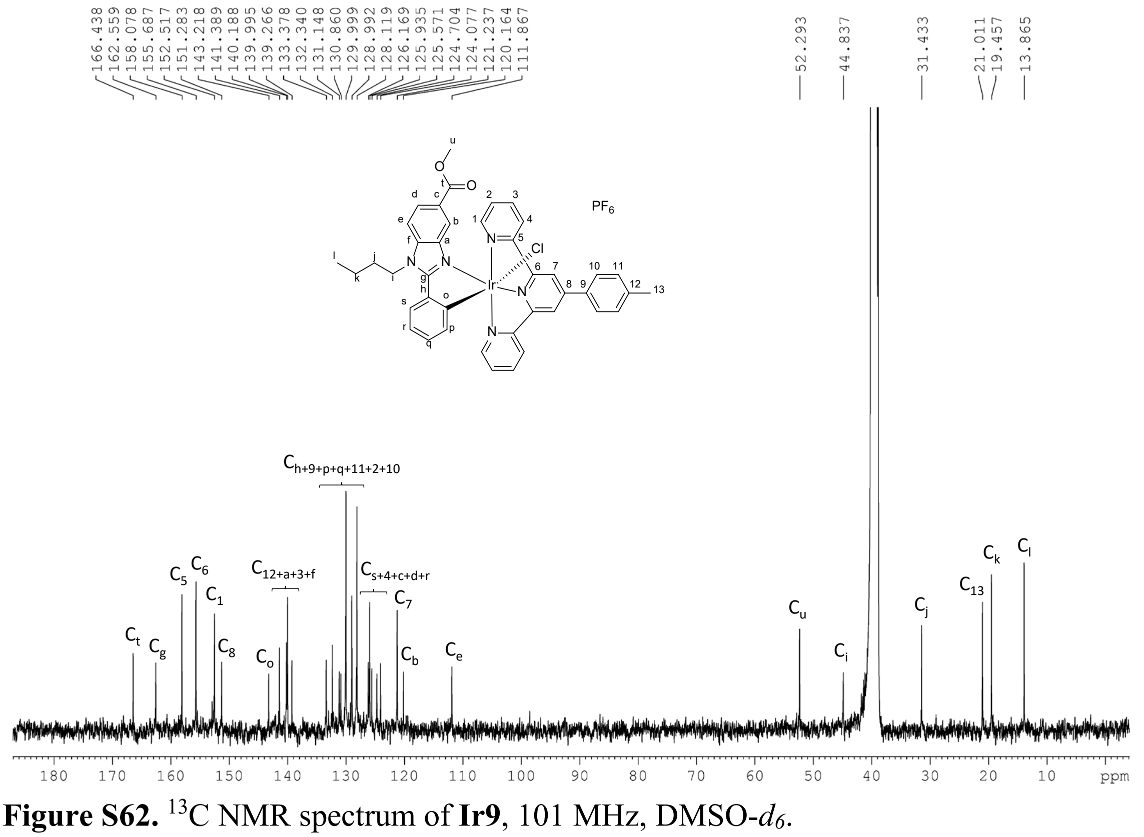

Figure S62. ^{13}C NMR spectrum of **Ir9**, 101 MHz, DMSO-d_6.

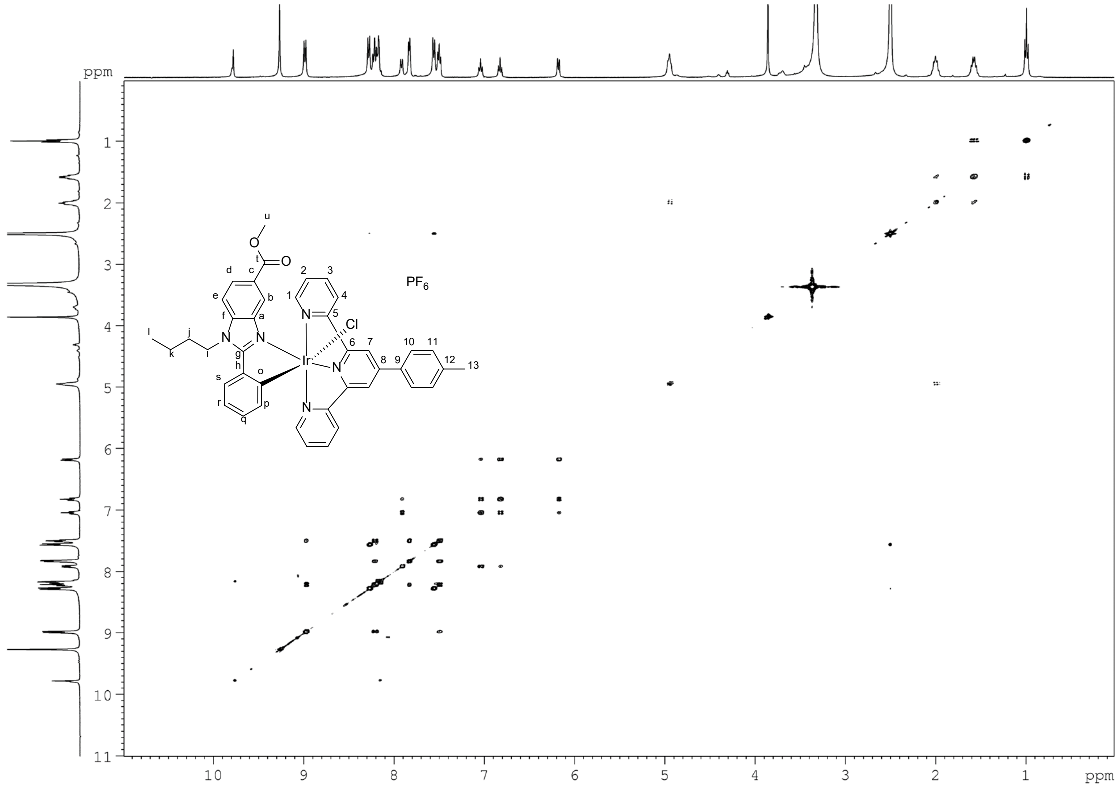

Figure S63. ^{1}H-^{1}H COSY NMR spectrum of **Ir9**, 600 MHz, DMSO-d_6.

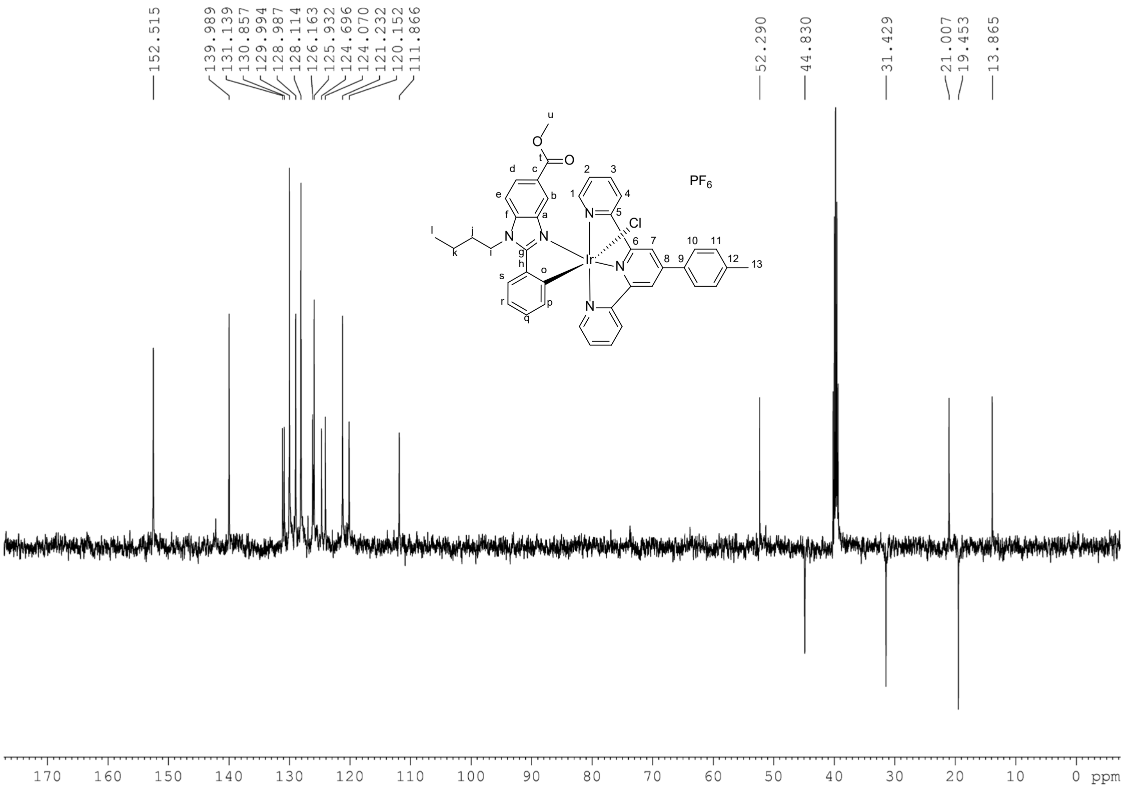

Figure S64. DEPT-135 NMR spectrum of **Ir9**, 600 MHz, DMSO-d_6.

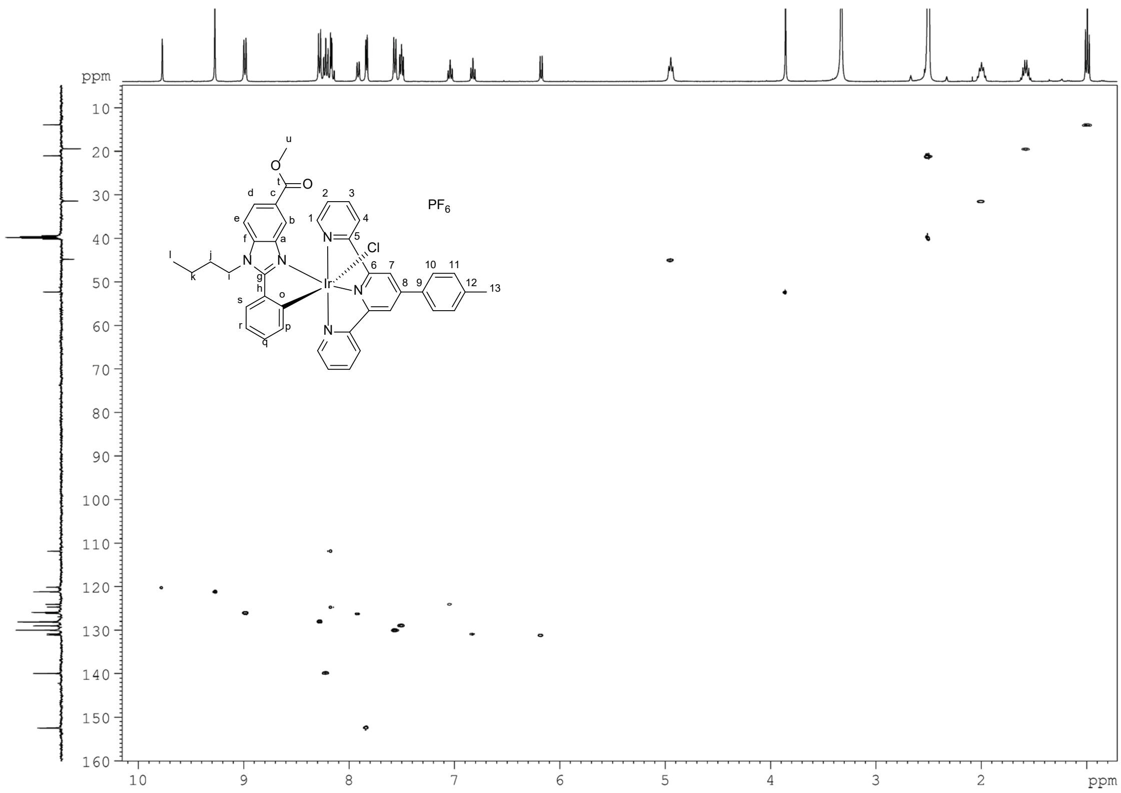

Figure S65. ^{1}H-^{13}C HSQC NMR spectrum of **Ir9**, 600 MHz, DMSO-d_6.

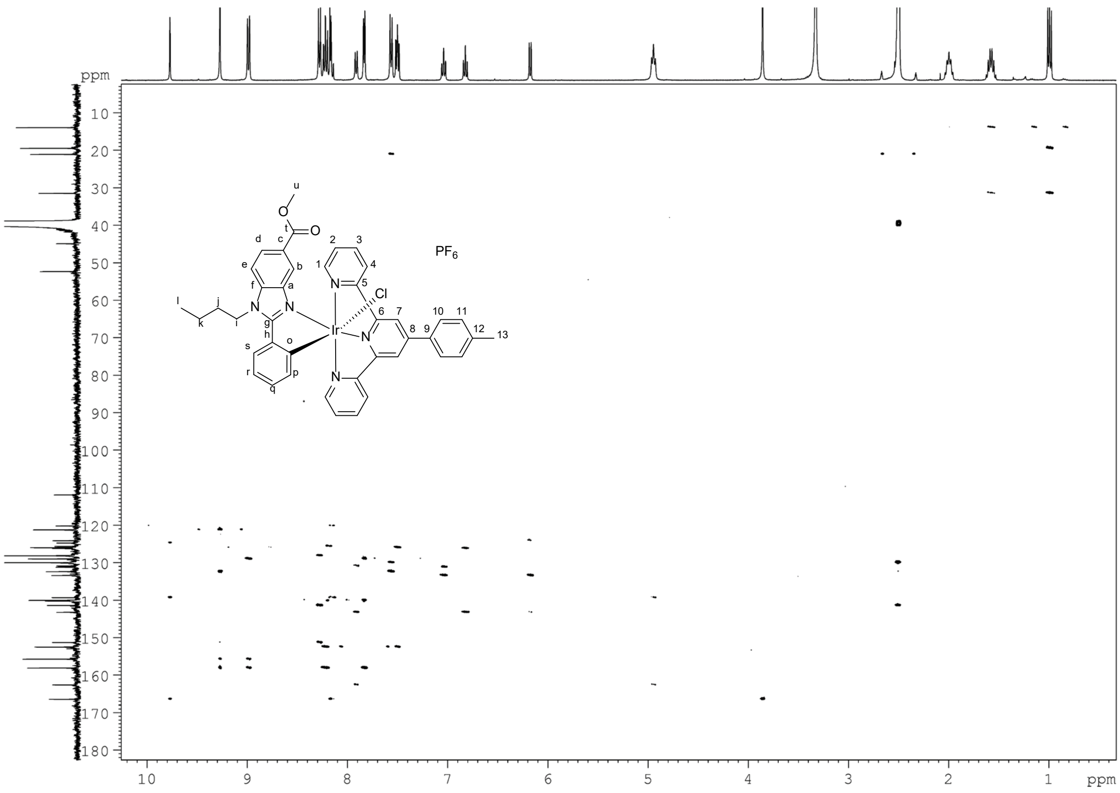

Figure S66. ^{1}H-^{13}C HMBC NMR spectrum of **Ir9**, 600 MHz, DMSO-d_6.

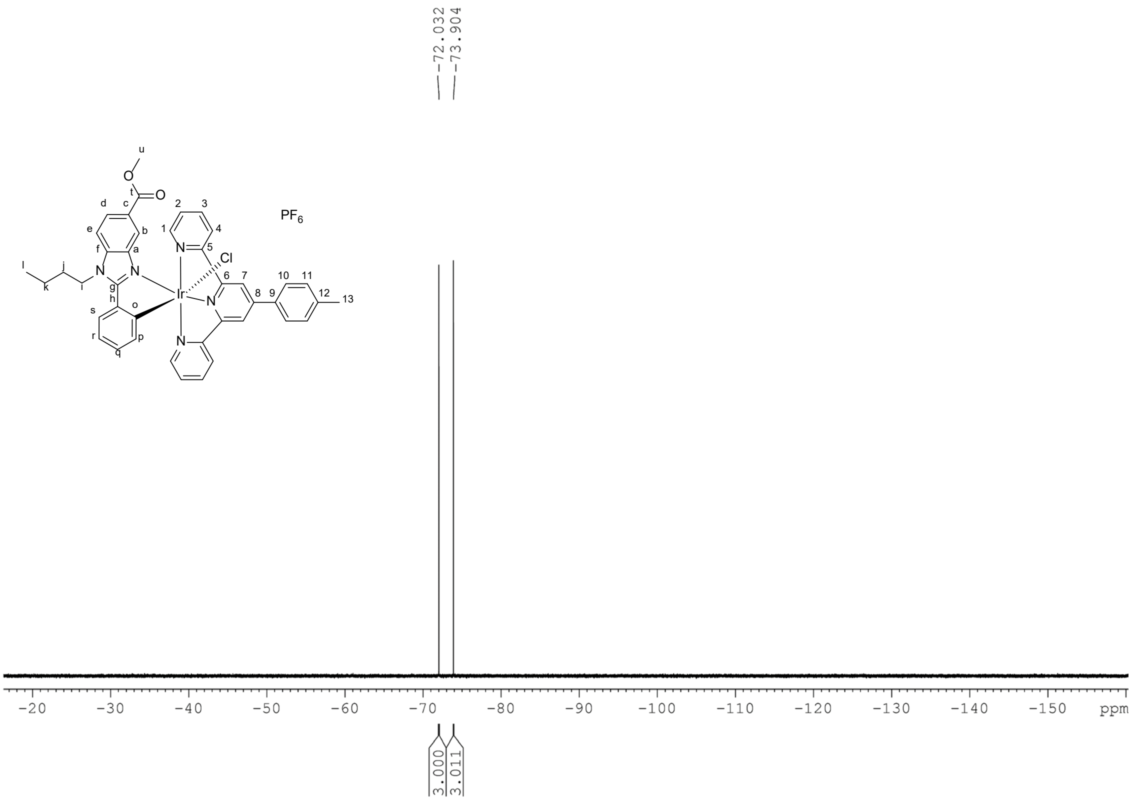

Figure S67. ^{19}F NMR spectrum of **Ir9**, 377 MHz, DMSO-d_6.

3. Mass spectrometry

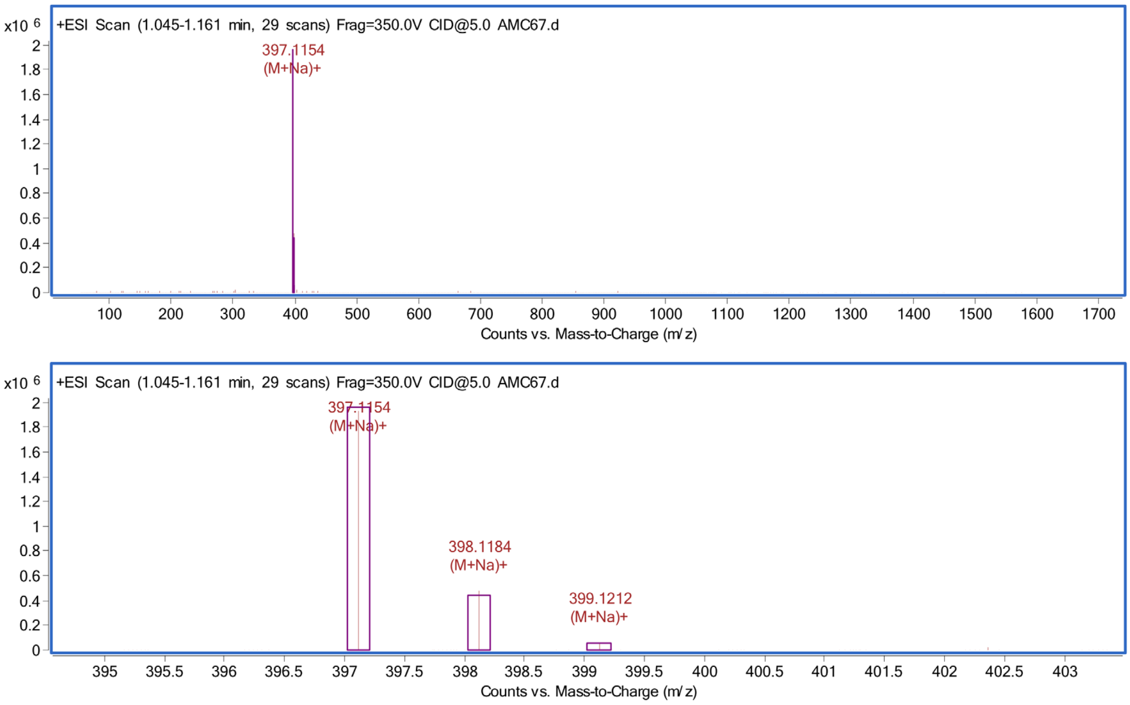

Figure S68. ESI-MS spectrum of **HL4** (positive detection mode).

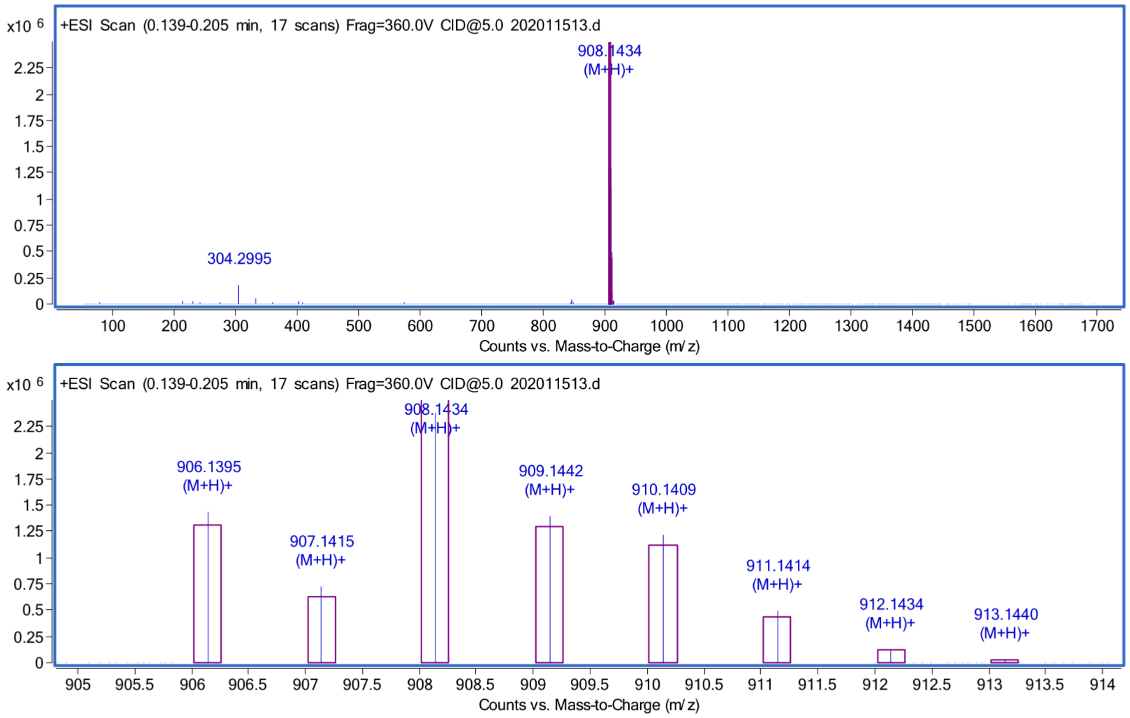

Figure S69. ESI-MS spectrum of **Ir1** (positive detection mode).

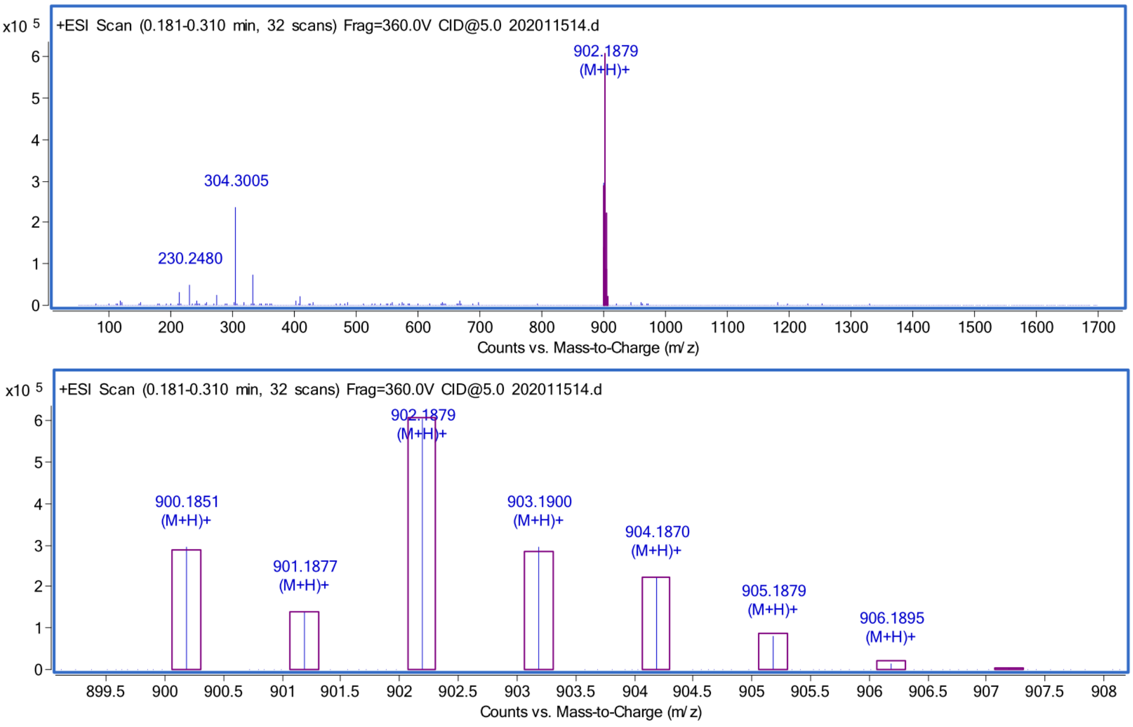

Figure S70. ESI-MS spectrum of **Ir2** (positive detection mode).

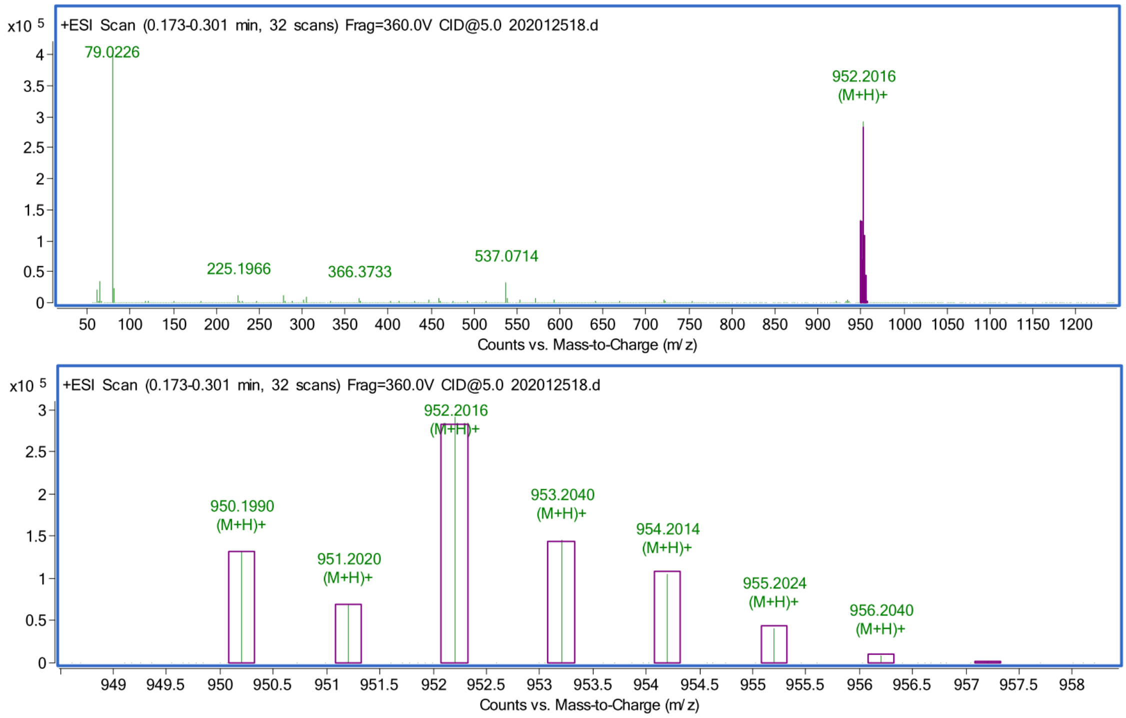

Figure S71. ESI-MS spectrum of **Ir3** (positive detection mode).

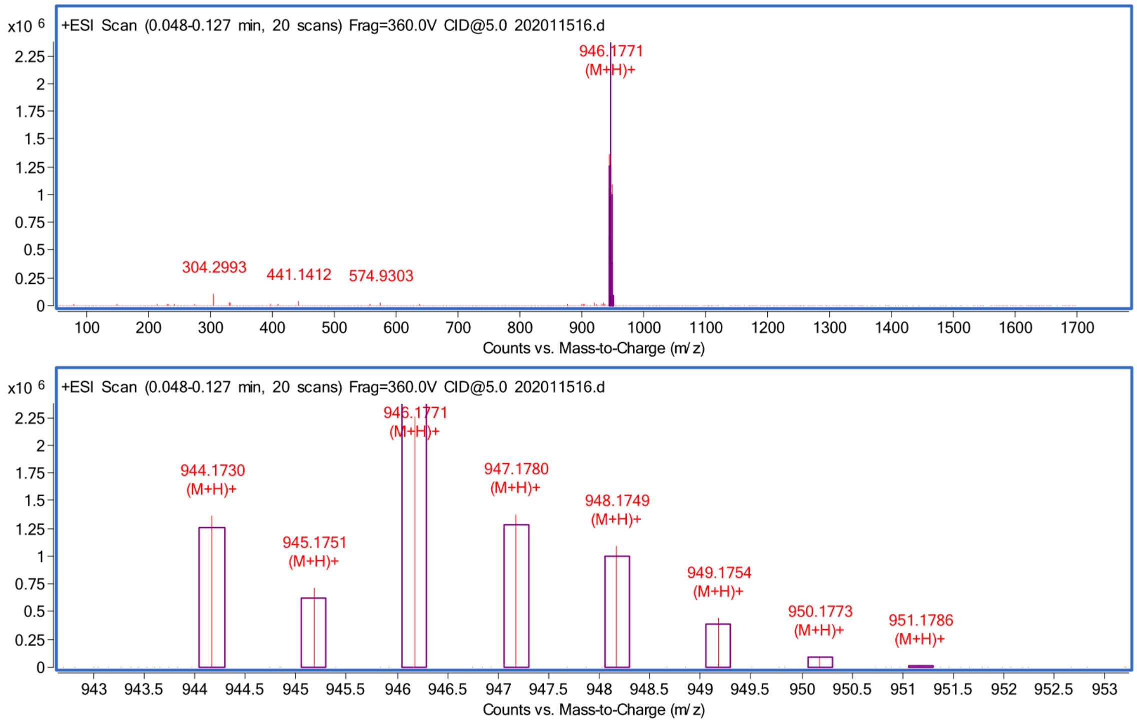

Figure S72. ESI-MS spectrum of **Ir4** (positive detection mode).

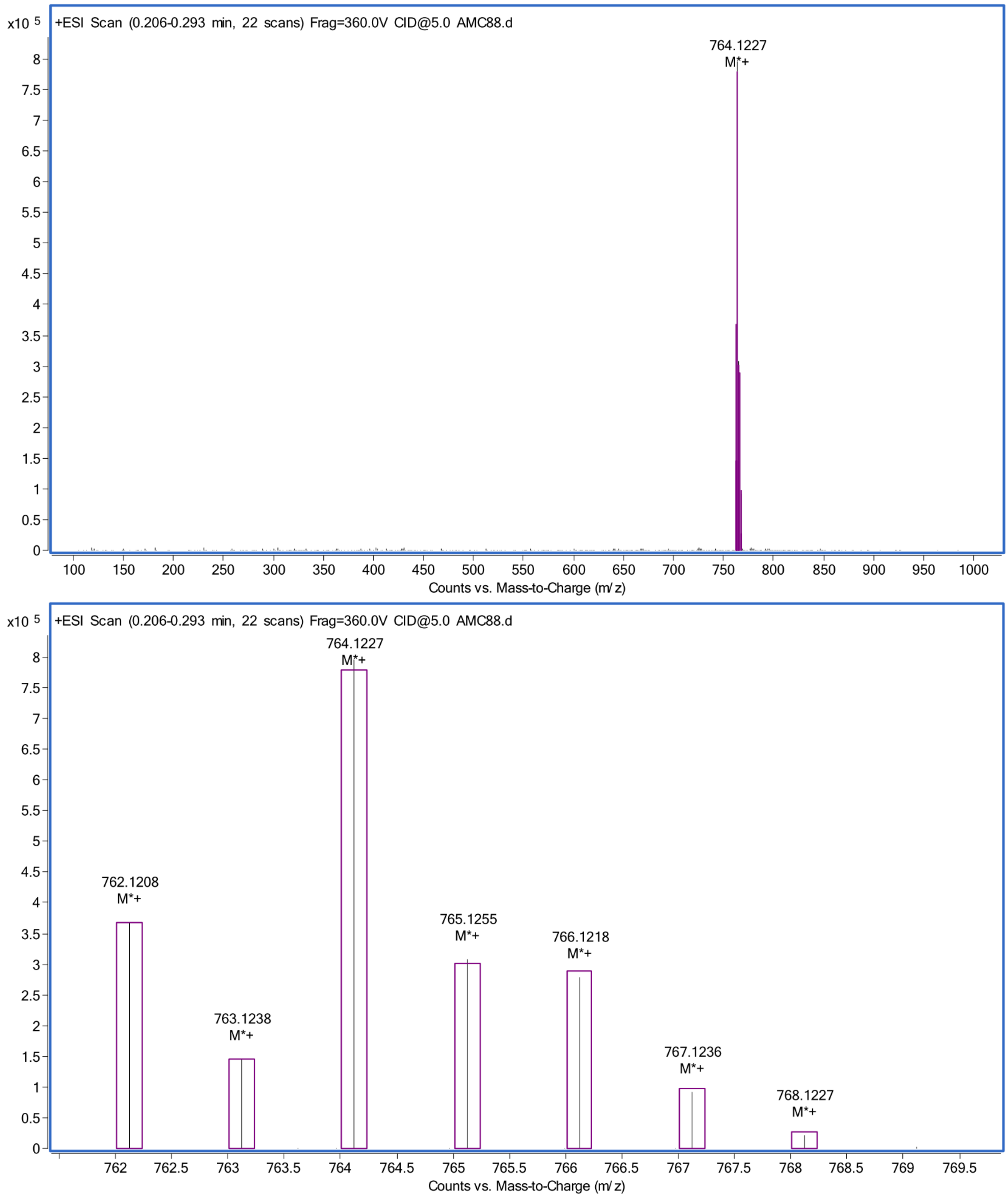

Figure S73. ESI-MS spectrum of **Ir5** (positive detection mode).

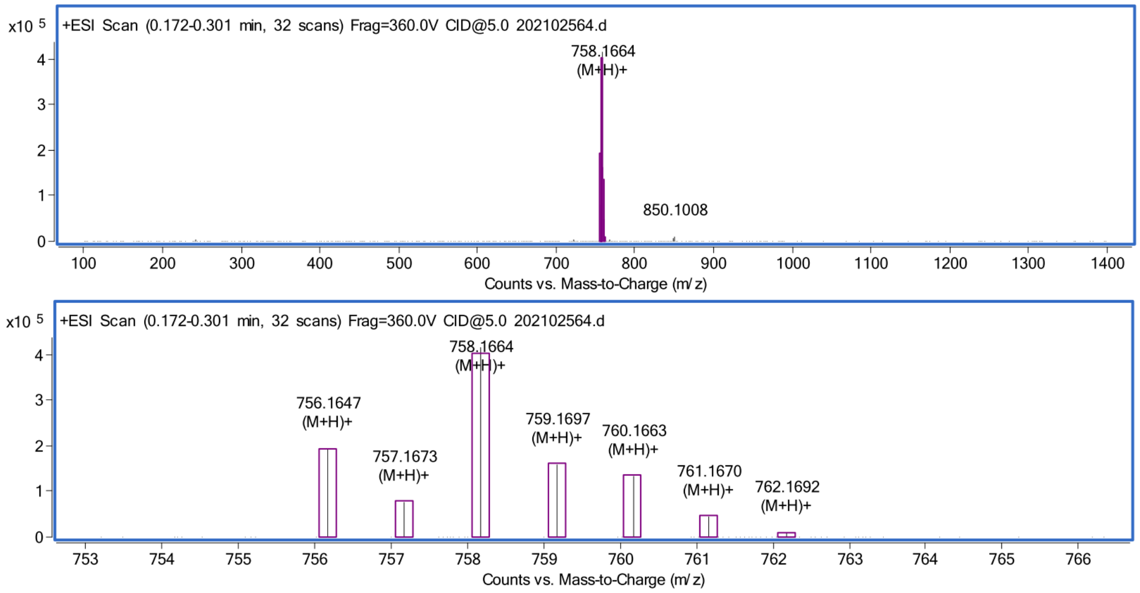

Figure S74. ESI-MS spectrum of **Ir6** (positive detection mode).

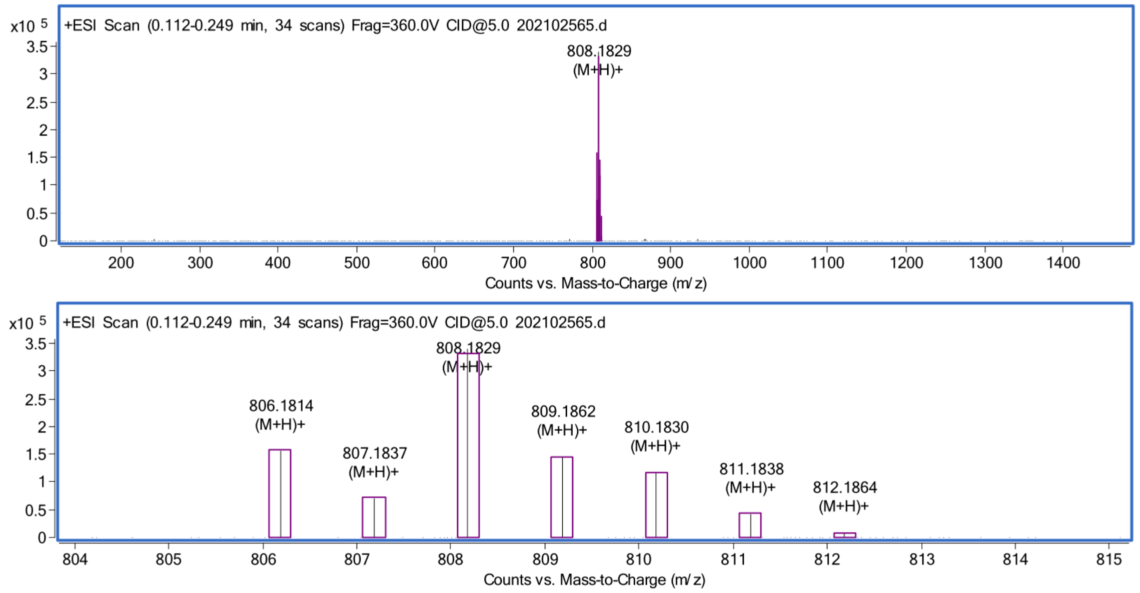

Figure S75. ESI-MS spectrum of **Ir7** (positive detection mode).

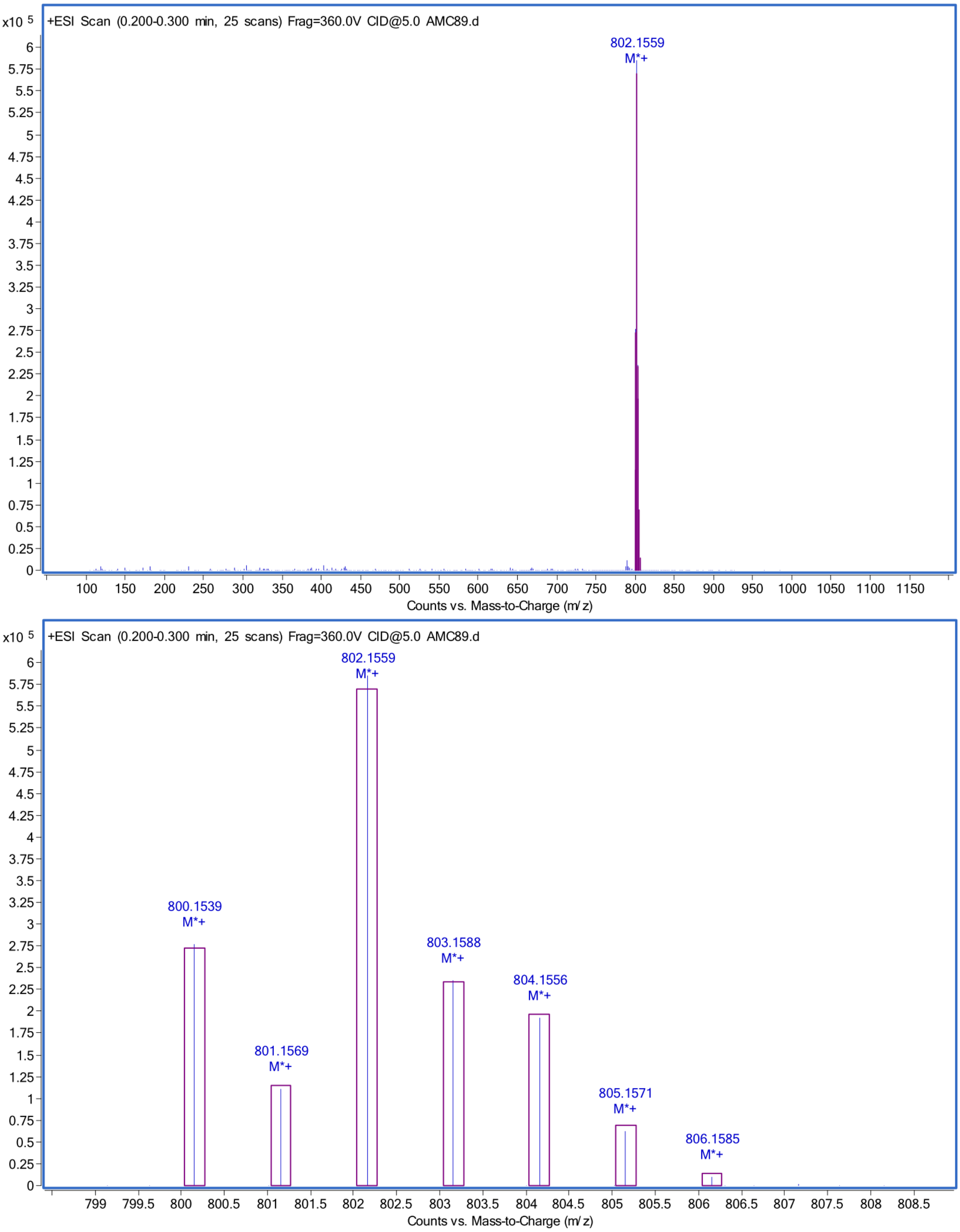

Figure S76. ESI-MS spectrum of **Ir8** (positive detection mode).

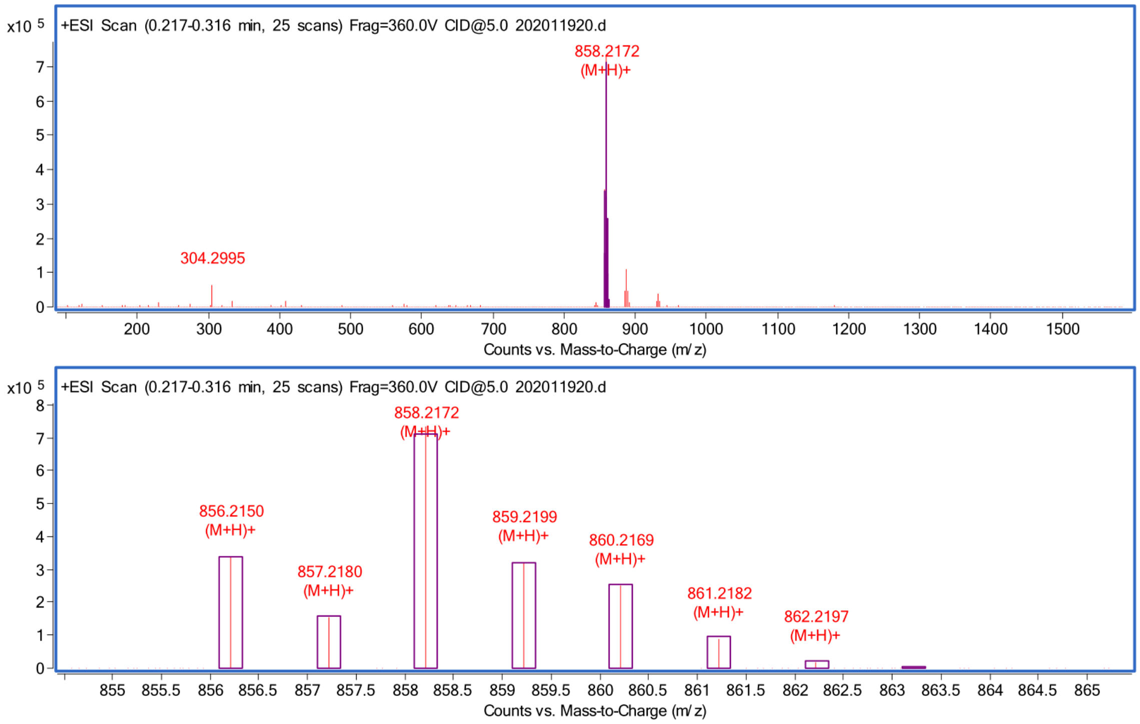

Figure S77. ESI-MS spectrum of **Ir9** (positive detection mode).

4. High performance liquid chromatography (HPLC) analysis

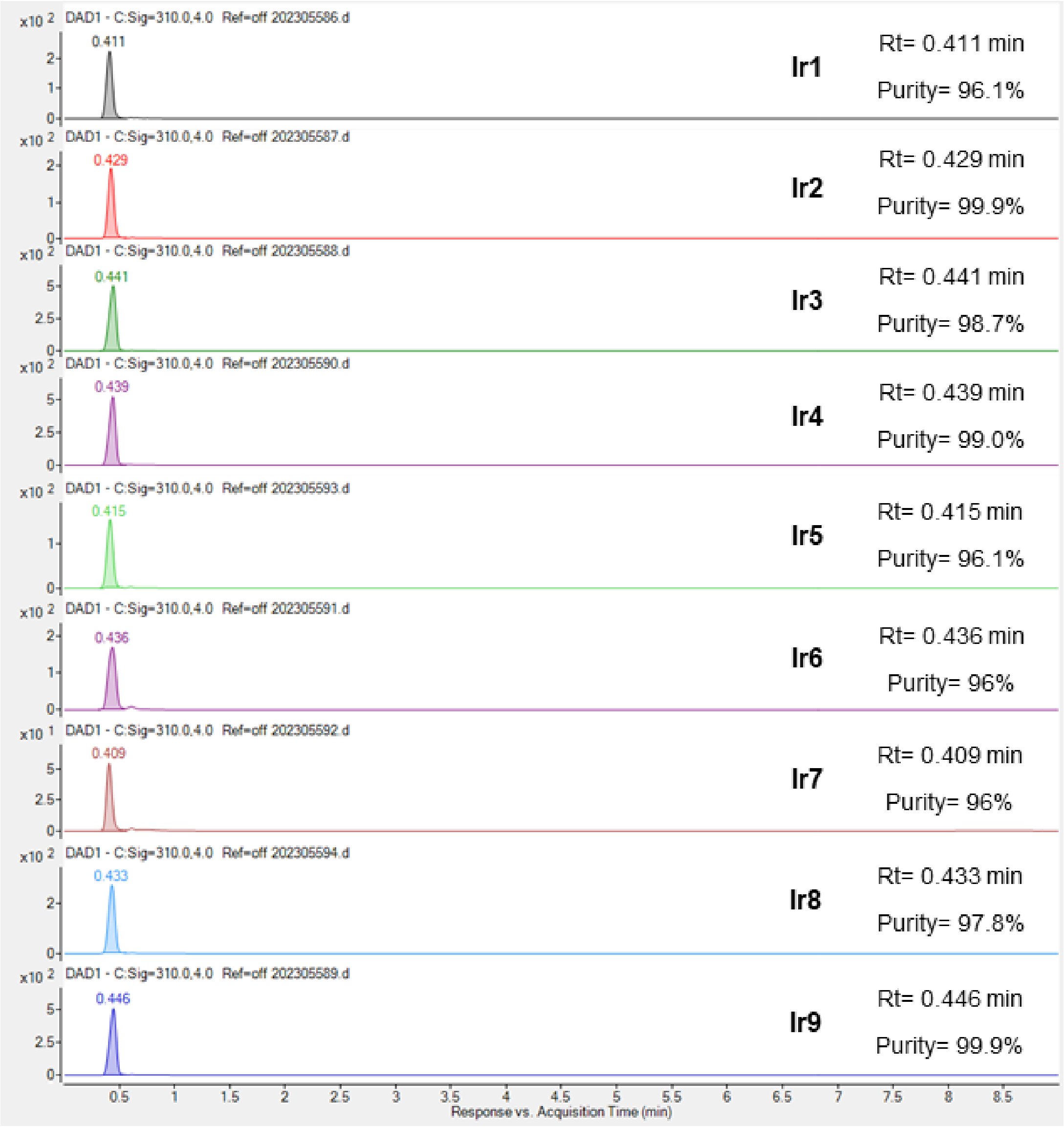

Figure S78. HPLC chromatograms with UV detection at 310 nm of complexes **Ir1–Ir9**. Acetonitrile/methanol (0.1% formic acid) (50/50) was used as the mobile phase.

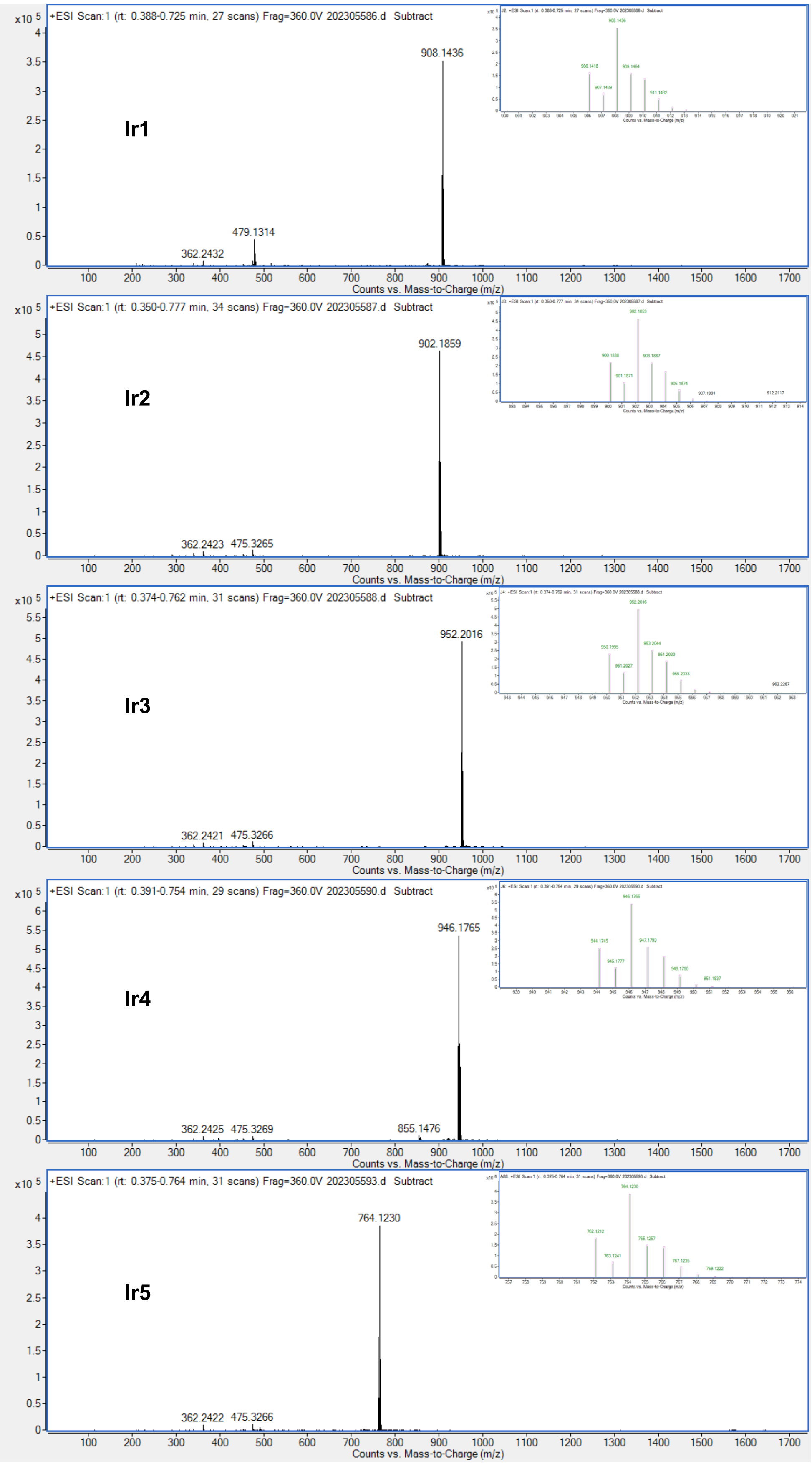
Ir1
+ESI Scan:1 (rt: 0.388-0.725 min, 27 scans) Frag=360.0V 202305586.d Subtract
908.1436
479.1314
362.2432
Ir2
+ESI Scan:1 (rt: 0.350-0.777 min, 34 scans) Frag=360.0V 202305587.d Subtract
902.1859
362.2423
475.3265
Ir3
+ESI Scan:1 (rt: 0.374-0.762 min, 31 scans) Frag=360.0V 202305588.d Subtract
952.2016
362.2421
475.3266
Ir4
+ESI Scan:1 (rt: 0.391-0.754 min, 29 scans) Frag=360.0V 202305590.d Subtract
946.1765
362.2425
475.3269
855.1476
Ir5
+ESI Scan:1 (rt: 0.375-0.764 min, 31 scans) Frag=360.0V 202305593.d Subtract
764.1230
362.2422
475.3266
Counts vs. Mass-to-Charge (m/z)

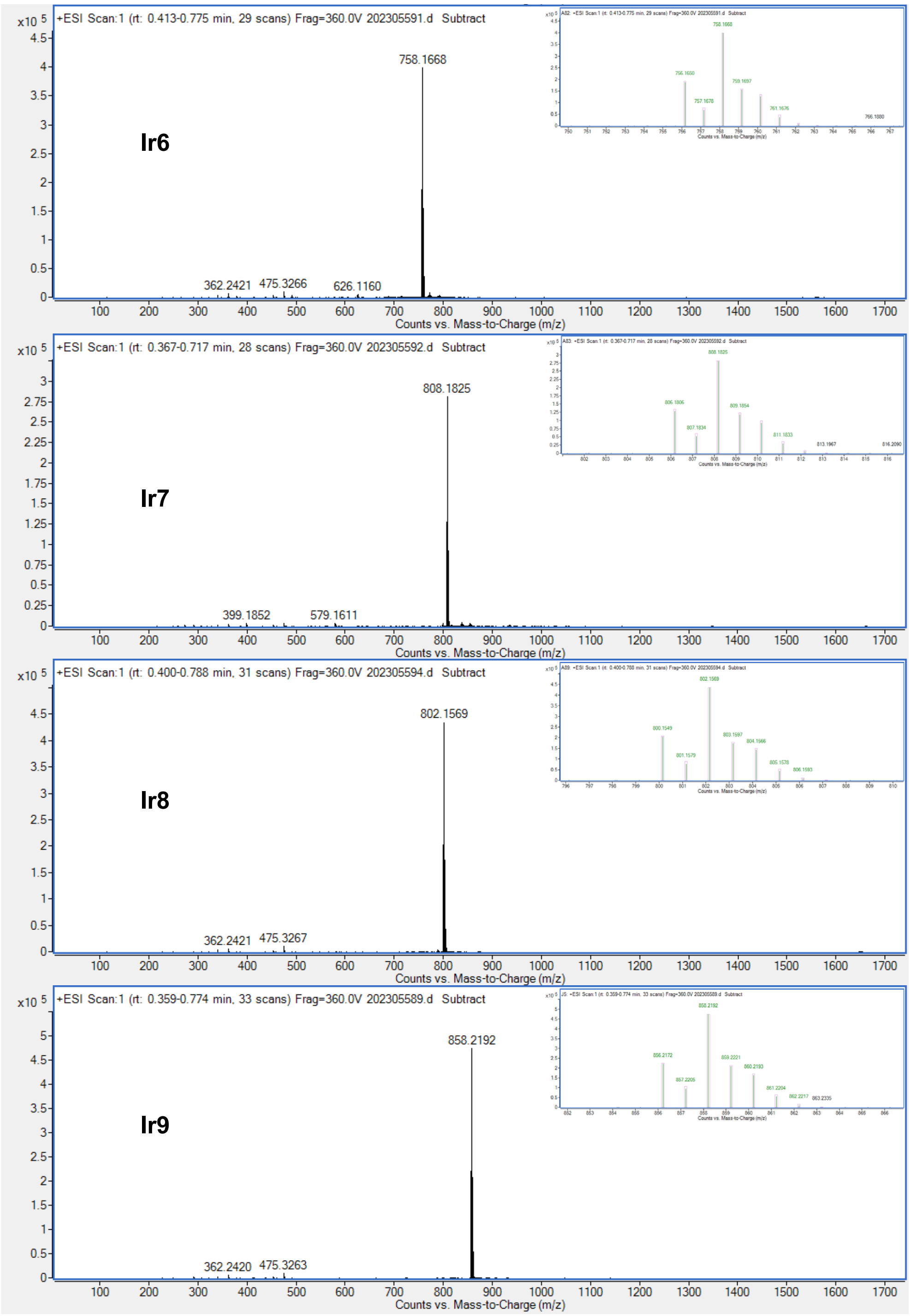

Figure S79. Mass spectra of the ~0.4 min peak of chromatograms of **Figure S78** with peak of interest extracted for all the complexes.

5. Photophysical properties

Table S1. Excitation (λ_{exc}), emission (λ_{em}) wavelengths, emission lifetimes (τ_{em}), and emission quantum yields (Φ_{em}) of complexes in deaerated acetonitrile.

Complex	$\lambda_{exc}{}^{a}$ (nm)	λ_{em} (nm)	$\tau_{em}{}^{b}$ (µs)	$\Phi_{em}{}^{b}$ (%)
**Ir1**	350	580	0.34	9.4
**Ir2**	370	555	2.62	75.8
**Ir3**	350	560	2.16	65.1
**Ir4**	370	545	0.73	20.9
**Ir5**	310	610	0.911 (89%)<br>2.98 (11%)	5
**Ir6**	370	560	5.99	60
**Ir7**	350	565	1.80 (74%)<br>2.42 (26%)	52.1
**Ir8**	330	565	0.589 (83%) 0.872 (17%)	17
**Ir9**	370	555	2.63	72.1

[a] λ_{exc} maxima. [b] Emission lifetimes ($\lambda_{NanoLED}$= 372 nm.) and quantum yields measured in deaerated solution.

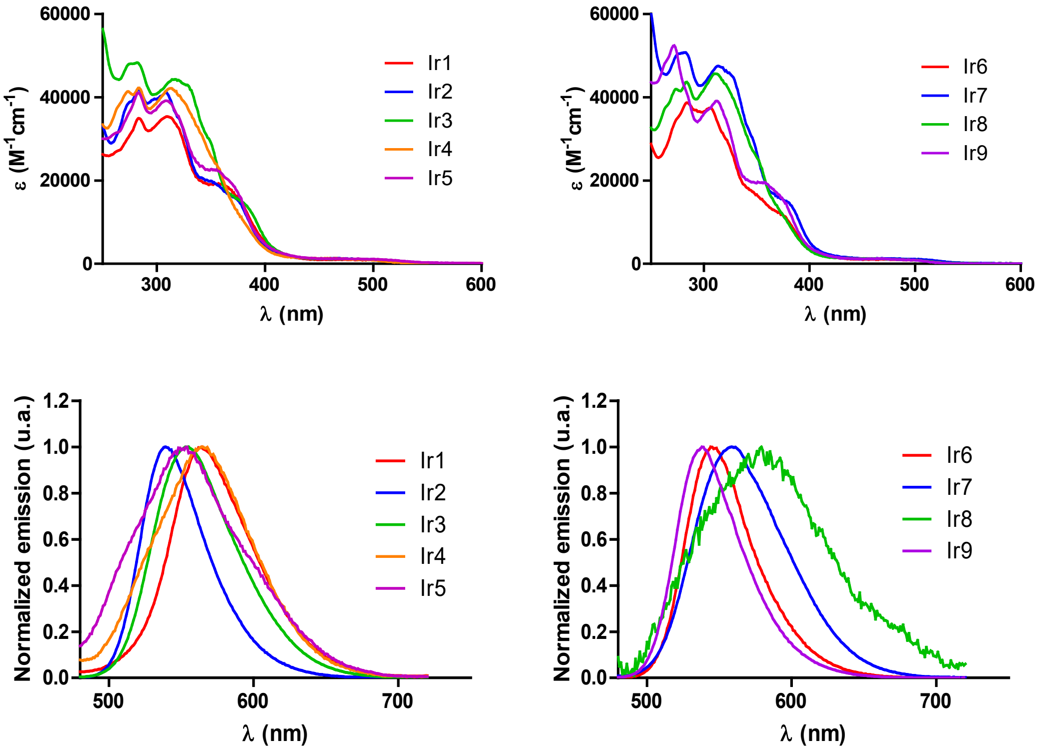

Figure S80. UV/Vis spectra (top) and normalized emission spectra (bottom) of complexes in aerated water (1% DMSO), λ_{exc}= 405 nm, 10 µM.

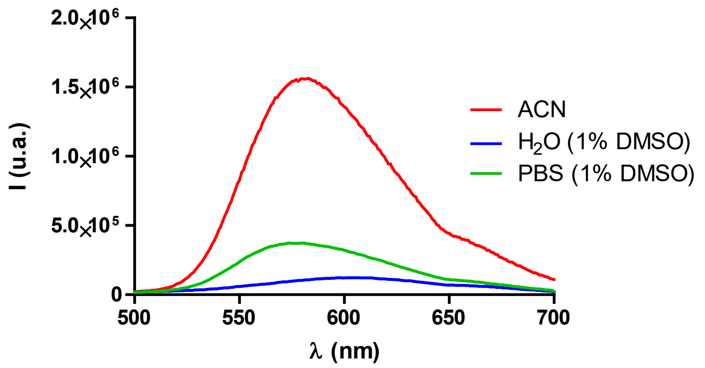

Figure S81. Emission spectra of complex **Ir1** in aerated acetonitrile, water (1% DMSO) and PBS (1% DMSO), λ_{exc}= 355 nm, 10 µM.

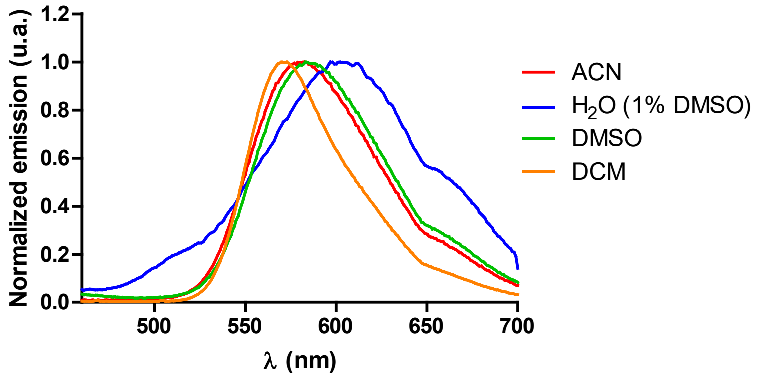

Figure S82. Emission spectra of complex **Ir1** in aerated acetonitrile, water (1% DMSO), DMSO and DCM, λ_{exc}= 355 nm, 10 µM.

6. Stability studies

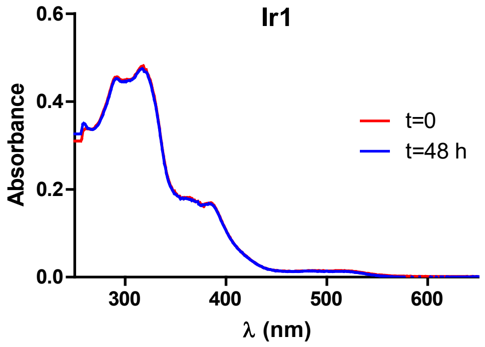

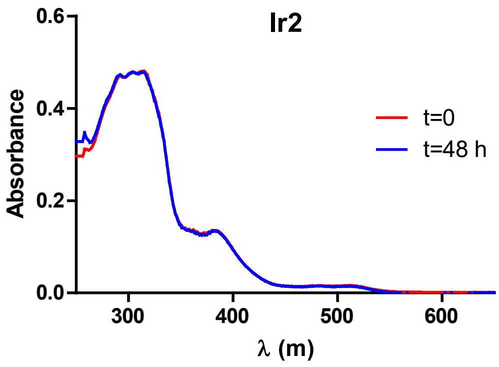

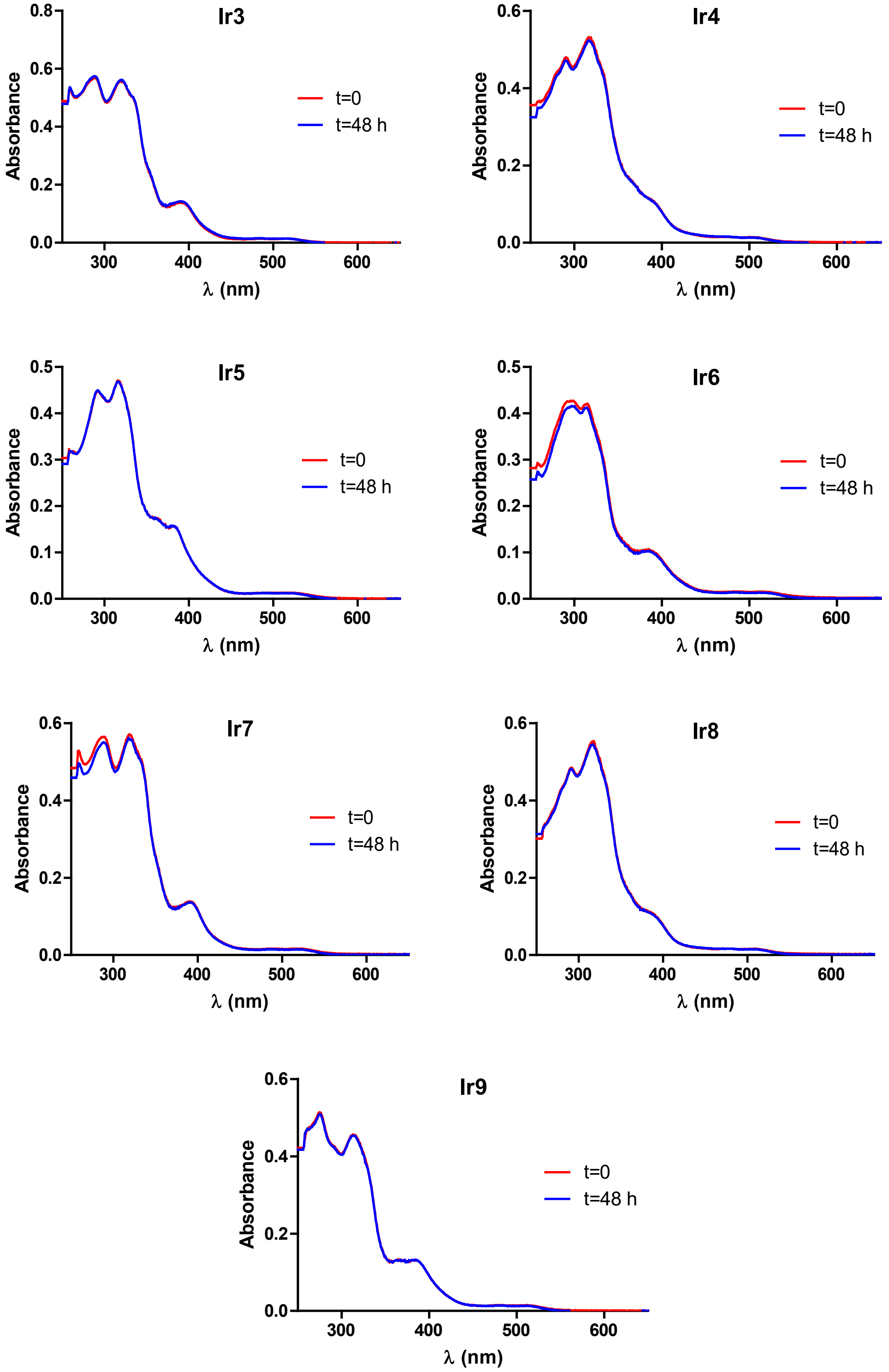

Figure S83. UV/Vis spectra of complexes **Ir1**–**Ir9** (10 μM) in DMSO at t=0 and after 48 h.

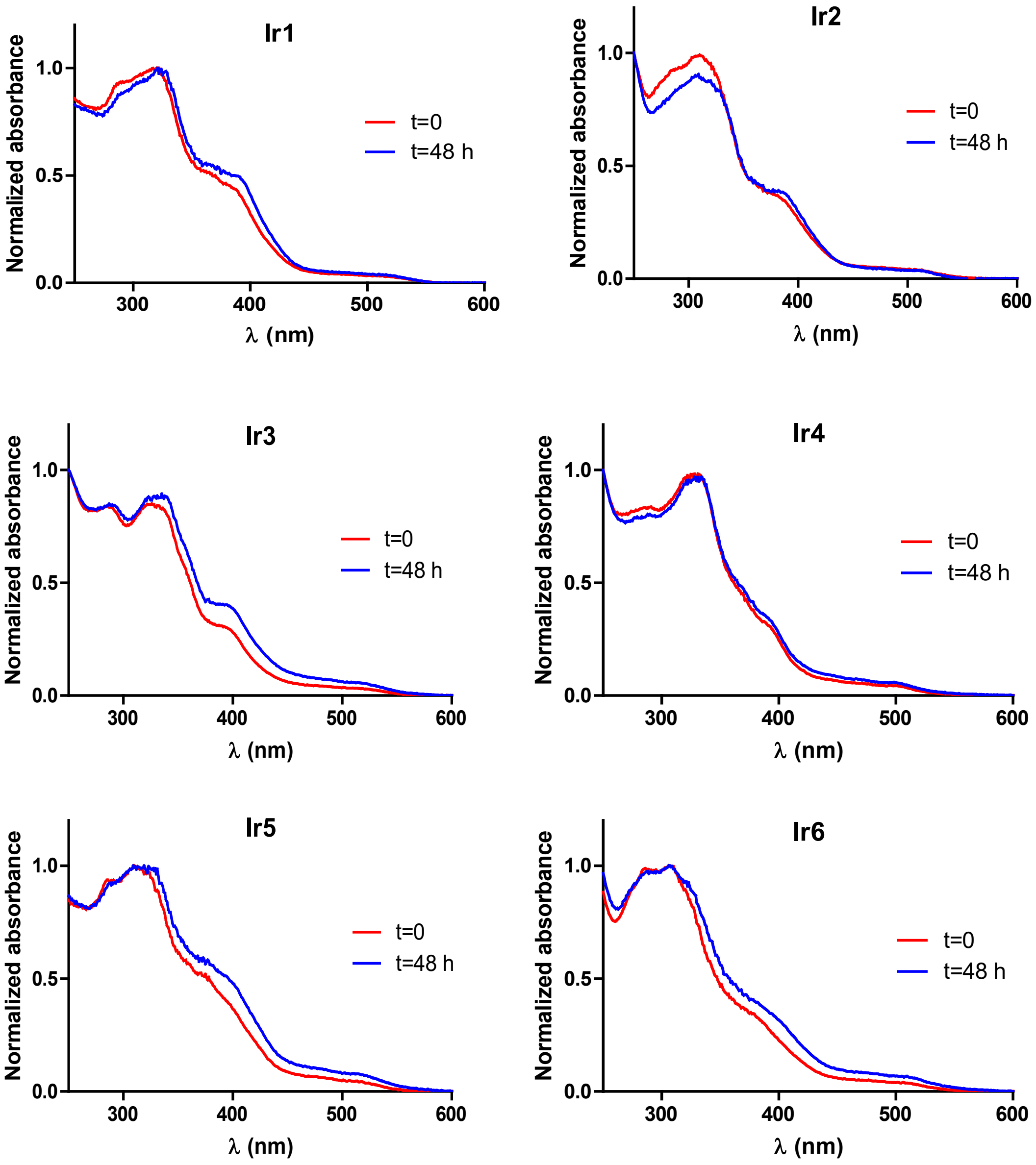
Ir1
Ir2
Ir3
Ir4
Ir5
Ir6
Normalized absorbance
λ (nm)
t=0
t=48 h
0.0
0.5
1.0
300
400
500
600

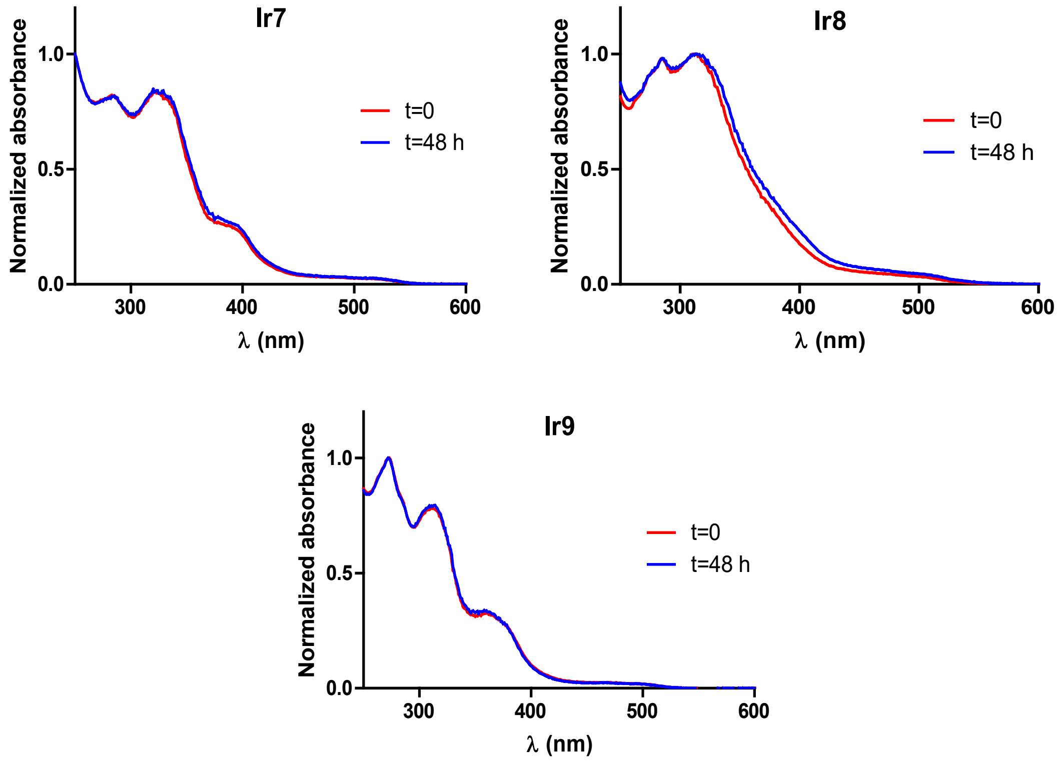

Figure S84. UV/Vis spectra of complexes **Ir1–Ir19** (10 μM) in RPMI (5% DMSO) at t= 0 h and after 48 h.

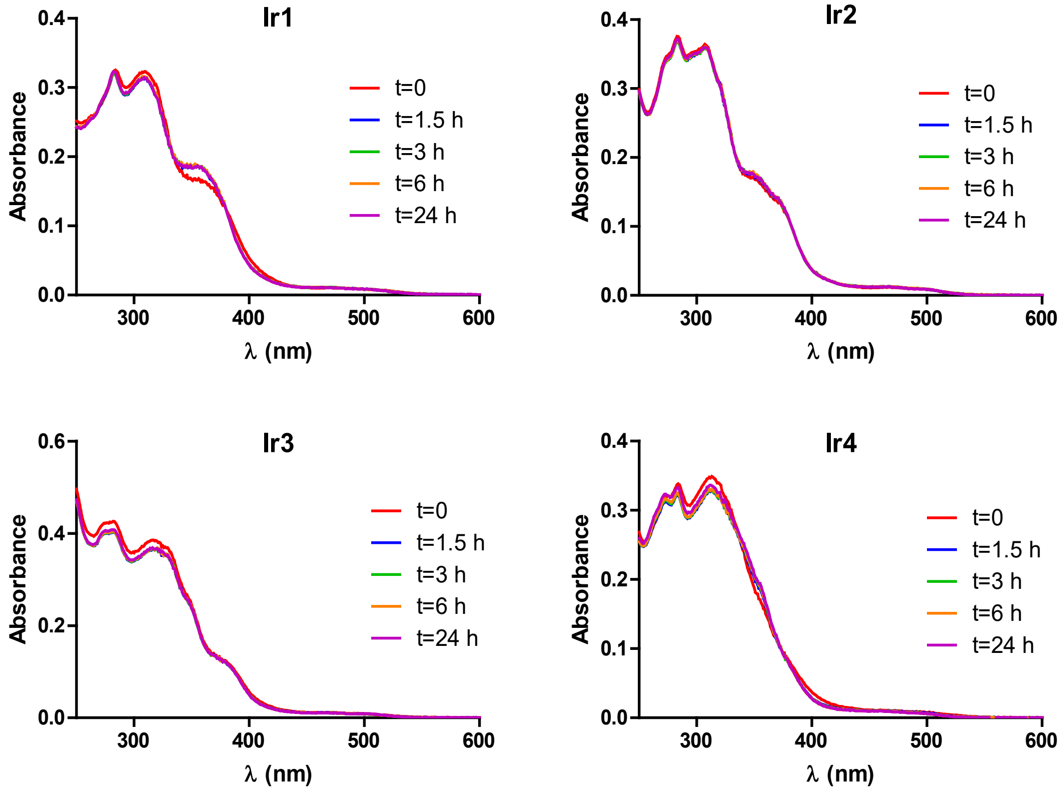

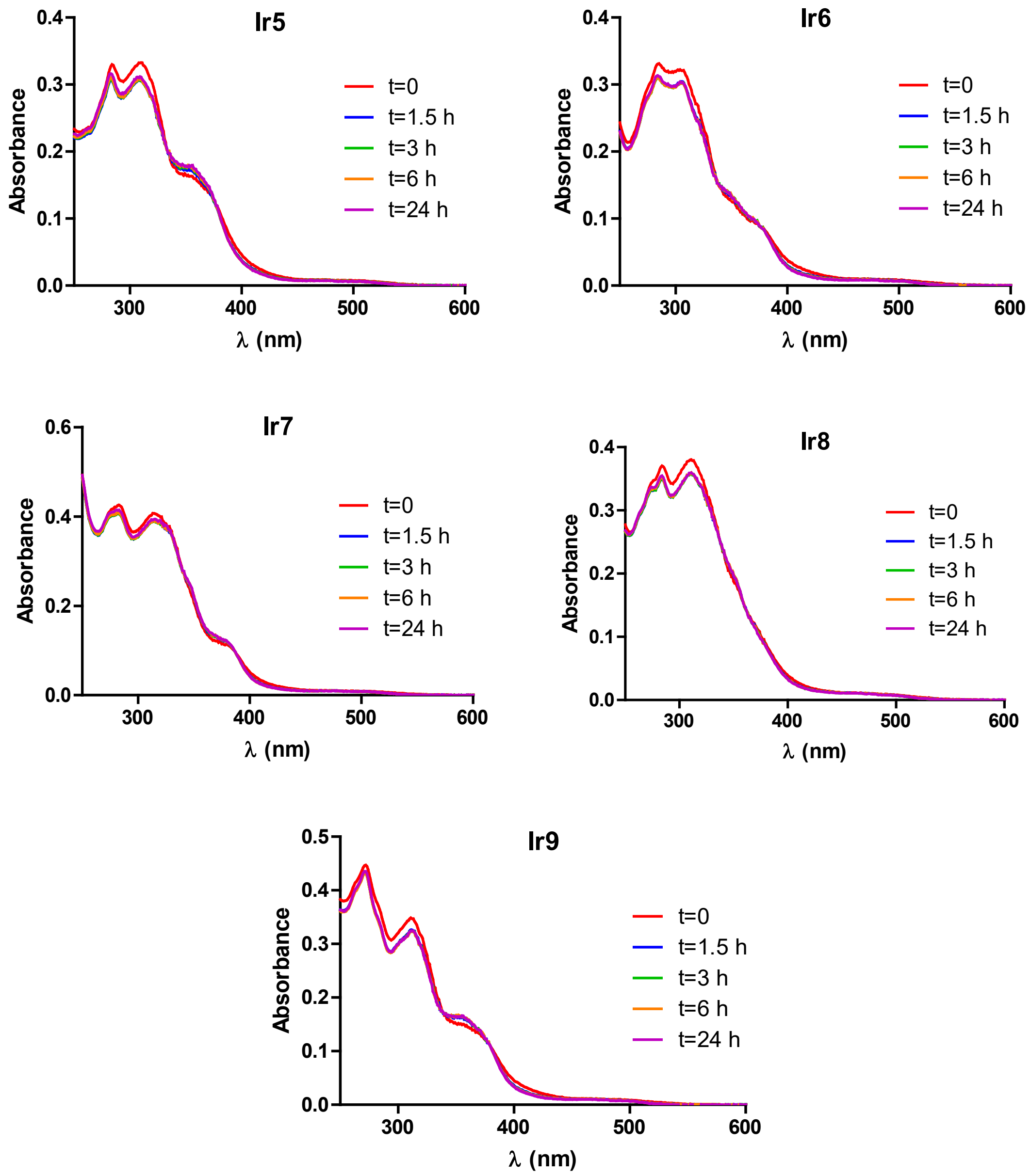

Figure S85. UV/Vis spectra of complexes **Ir1**–**Ir9** (10 μM) in water (1% DMSO) at different times.

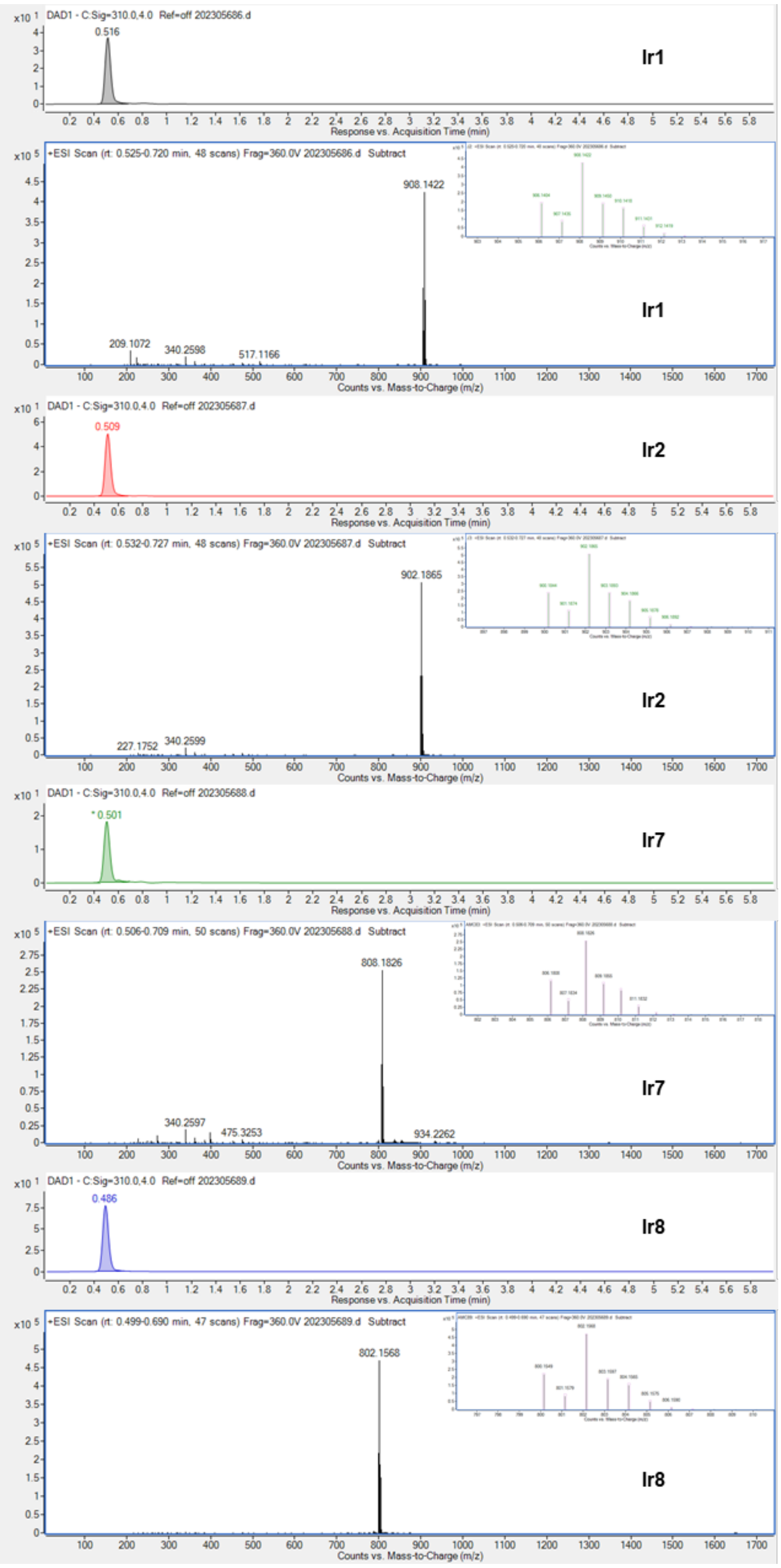

Figure S86. HPLC/MS spectra of complexes **Ir1**, **Ir2**, **Ir7** and **Ir8** with UV detection at 310 nm and the corresponding ESI-MS spectrum with the [M-PF_6]$^+$ peak. Acetonitrile/water (0.1% formic acid) (80/20) was used as the mobile phase.

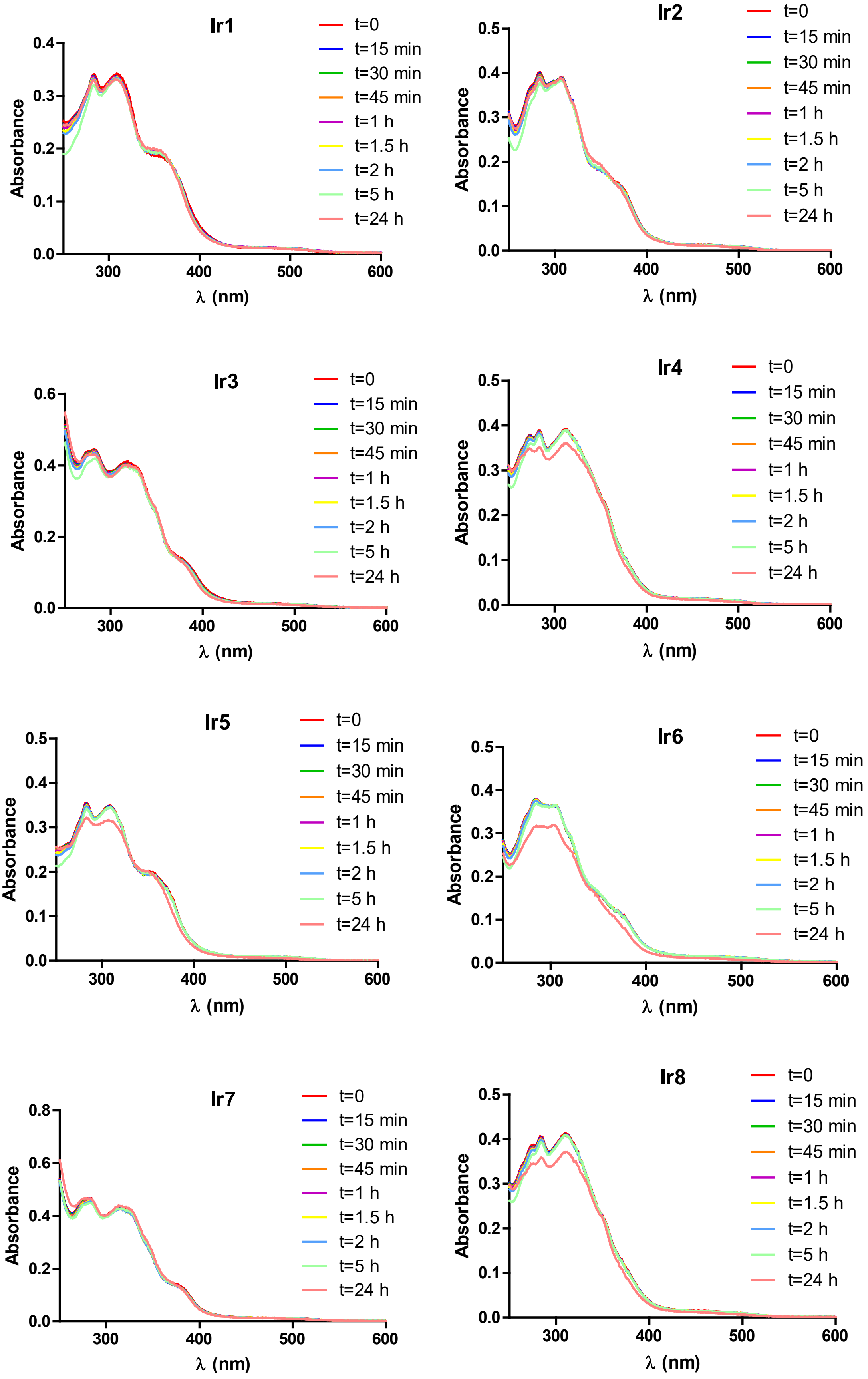
Ir1
Ir2
Ir3
Ir4
Ir5
Ir6
Ir7
Ir8
t=0
t=15 min
t=30 min
t=45 min
t=1 h
t=1.5 h
t=2 h
t=5 h
t=24 h
Absorbance
λ (nm)
300
400
500
600

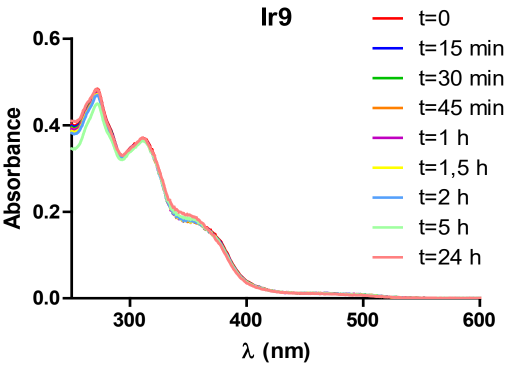

Figure S87. UV/Vis spectra of complexes **Ir1**–**Ir9** (10 µM) in water (1% DMSO) and excess of GSH (10 mM) at different times.

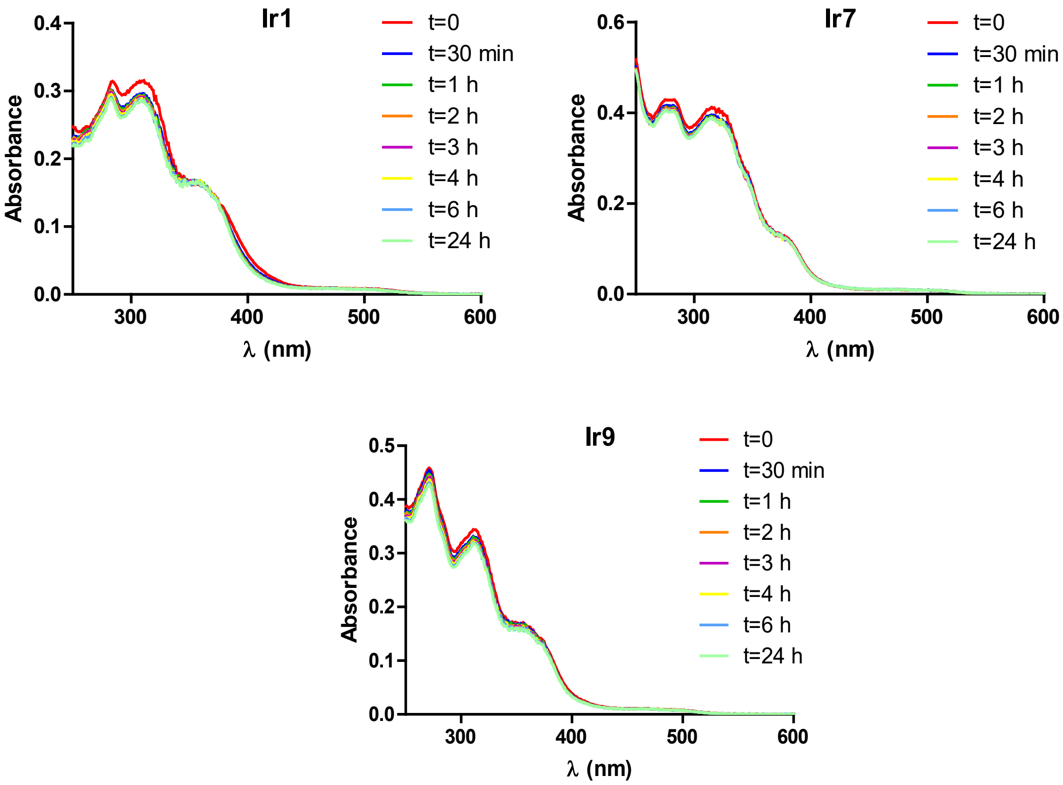

Figure S88. UV/Vis spectra of complexes **Ir1**, **Ir7** and **Ir9** (10 µM) in water (1% DMSO) and excess of NADH (100 µM) at different times.

Table S2. Statistical analysis (P values determined by two-way ANOVA) of the antiproliferative activity (IC_{50} values) of the investigated compounds.

P value (Two-way ANOVA)	MDA-MB-231 vs MCF-7	MDA-MB-231 vs MCF-10A	MCF-7 vs MCF-10A
**Ir1**	0.9759	≤0.0001	≤0.0001
**Ir2**	0.9144	≤0.0001	≤0.0001
**Ir3**	0.9939	≤0.0001	≤0.0001
**Ir4**	0.9892	≤0.0001	≤0.0001
**Ir5**	0.6034	≤0.0001	≤0.0001
**Ir6**	0.1817	≤0.0001	≤0.0001
**Ir7**	0.5925	≤0.0001	≤0.0001
**Ir8**	0.9939	≤0.0001	≤0.0001
**Ir9**	0.9214	≤0.0001	≤0.0001
**Dox**	0.9878	0.0005	0.0003

Table S3. Statistical analysis (P values determined by one-way ANOVA). Multiple comparison of antiproliferative activity (IC_{50} values) of the investigated compounds in MDA-MB-231 cells.

P-Value	Ir1	Ir2	Ir3	Ir4	Ir5	Ir6	Ir7	Ir8	Ir9
**Ir1**	-	-	-	-	-	-	-	-	-
**Ir2**	0.9997	-	-	-	-	-	-	-	-
**Ir3**	0.0082	0.0021	-	-	-	-	-	-	-
**Ir4**	0.4884	0.8473	<0.0001	-	-	-	-	-	-
**Ir5**	0.8905	0.9968	0.0003	0.9988	-	-	-	-	-
**Ir6**	0.9997	0.9527	0.0314	0.1929	0.5514	-	-	-	-
**Ir7**	<0.0001	<0.0001	0.1332	<0.0001	<0.0001	<0.0001	-	-	-
**Ir8**	>0.9999	>0.9999	0.0052	0.6154	0.9527	0.9968	<0.0001	-	-
**Ir9**	0.0013	0.0003	0.9968	<0.0001	<0.0001	0.0052	0.4884	0.0008	-
**Dox**	0.2108	0.5196	<0.0001	0.9998	0.9402	0.0662	<0.0001	0.295	<0.0001

Table S4. Statistical analysis (P values determined by one-way ANOVA). Multiple comparison of antiproliferative activity (IC_{50} values) of the investigated compounds in MCF-7 cells.

P-Value	Ir1	Ir2	Ir3	Ir4	Ir5	Ir6	Ir7	Ir8	Ir9
**Ir1**	-	-	-	-	-	-	-	-	-
**Ir2**	0.0338	-	-	-	-	-	-	-	-
**Ir3**	0.0002	0.3521	-	-	-	-	-	-	-
**Ir4**	>0.9999	0.0258	0.0001	-	-	-	-	-	-
**Ir5**	<0.0001	0.1208	0.9996	<0.0001	-	-	-	-	-
**Ir6**	<0.0001	<0.0001	<0.0001	<0.0001	<0.0001	-	-	-	-
**Ir7**	0.0572	>0.9999	0.2372	0.0441	0.0739	<0.0001	-	-	-
**Ir8**	>0.9999	0.0948	0.0005	0.9996	0.0001	<0.0001	0.1527	-	-
**Ir9**	0.0016	0.9083	0.9864	0.0012	0.7976	<0.0001	0.7976	0.0049	-
**Dox**	0.1527	<0.0001	<0.0001	0.1912	<0.0001	<0.0001	<0.0001	0.0572	<0.0001

Table S5. Statistical analysis (P values determined by one-way ANOVA). Multiple comparison of antiproliferative activity (IC_{50} values) of the investigated compounds in MCF-10A cells.

P-Value	Ir1	Ir2	Ir3	Ir4	Ir5	Ir6	Ir7	Ir8	Ir9
**Ir1**	-	-	-	-	-	-	-	-	-
**Ir2**	0.9904	-	-	-	-	-	-	-	-
**Ir3**	0.0081	0.0635	-	-	-	-	-	-	-
**Ir4**	0.6356	0.9904	0.3523	-	-	-	-	-	-
**Ir5**	0.9493	>0.9999	0.112	0.9993	-	-	-	-	-
**Ir6**	0.6356	0.9904	0.3523	>0.9999	0.9993	-	-	-	-
**Ir7**	>0.9999	0.9993	0.0153	0.803	0.9904	0.803	-	-	-
**Ir8**	0.9493	>0.9999	0.112	0.9993	>0.9999	0.9993	0.9904	-	-
**Ir9**	0.5163	0.9685	0.4586	>0.9999	0.9955	>0.9999	0.6945	0.9955	-
**Dox**	<0.0001	0.0004	0.3878	0.0032	0.0007	0.0032	<0.0001	0.0007	0.005

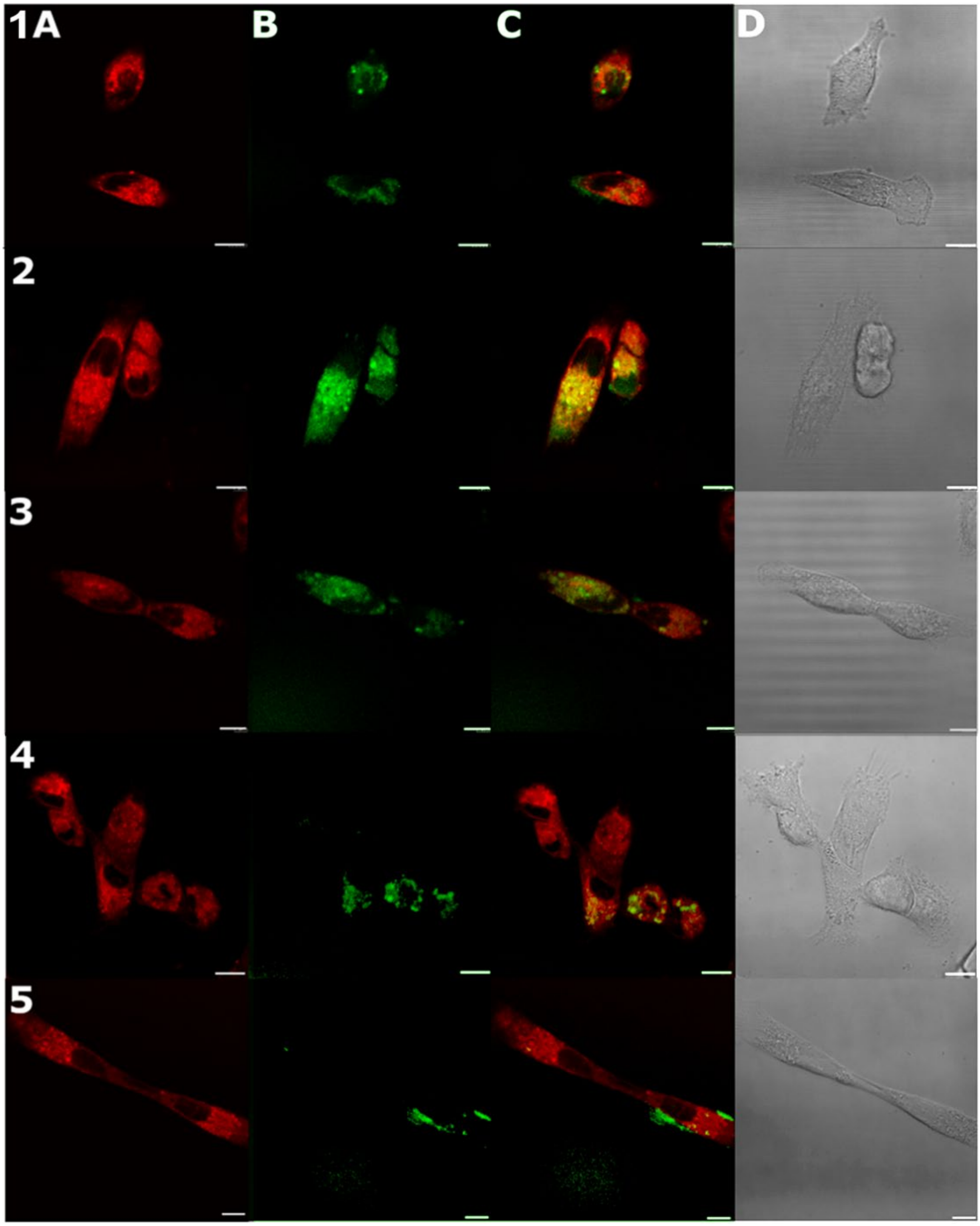
1A
B
C
D
2
3
4
5

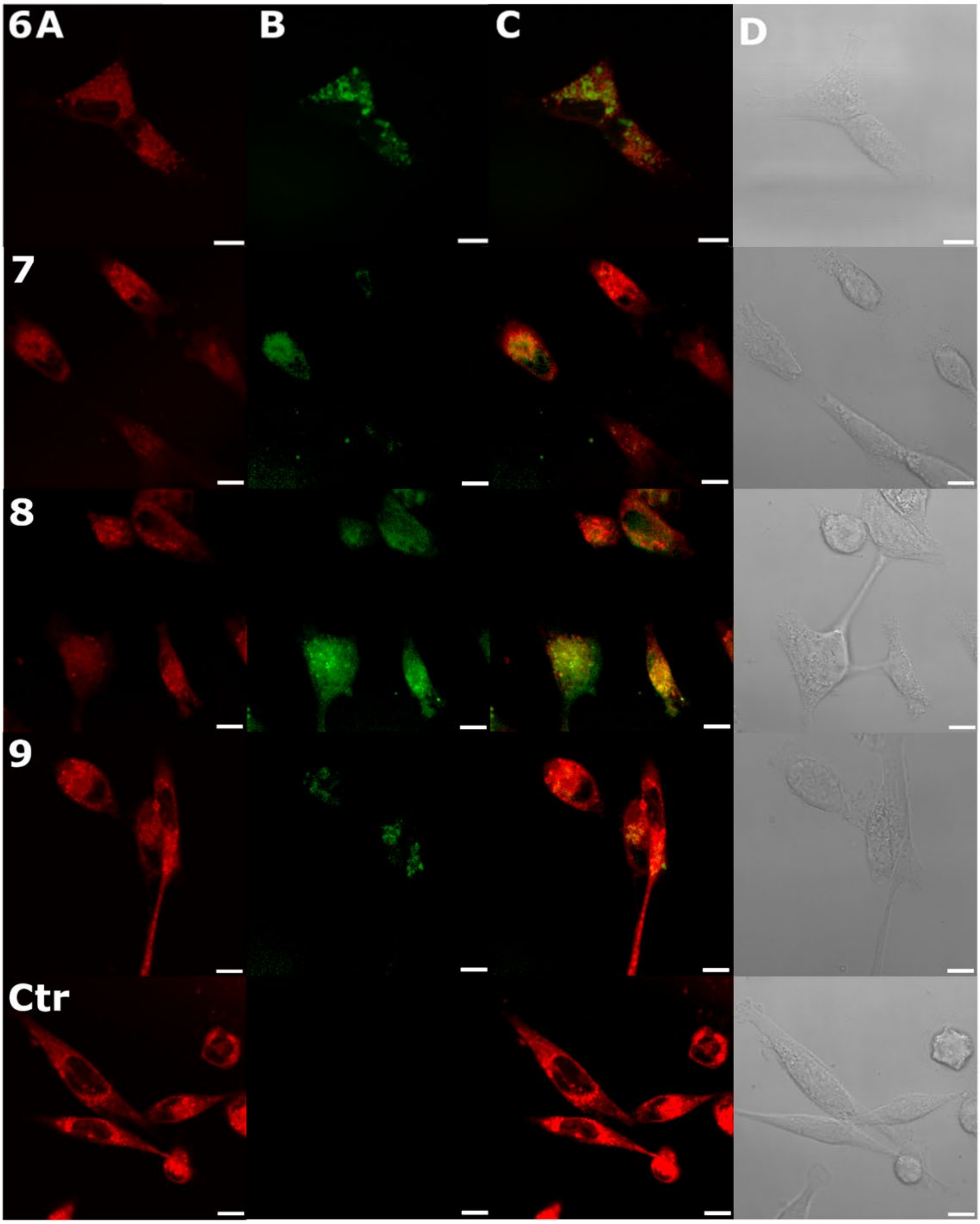

Figure S89. Colocalization study of the investigated Ir complexes in MDA-MB-231 cells. Samples were treated with tested compounds at the concentration of 5μM for 5 h and co-stained with MitoTracker™ Red. Channel A: Fluorescence coming from MitoTracker™ Red, channel B: fluorescence coming from tested compounds or vehicle-treated samples with the same setting of exc./em. scanning parameters, channel C: overlay of the A and B fluorescence channels, channel D: bright field. Scale bars represent 10 μm.

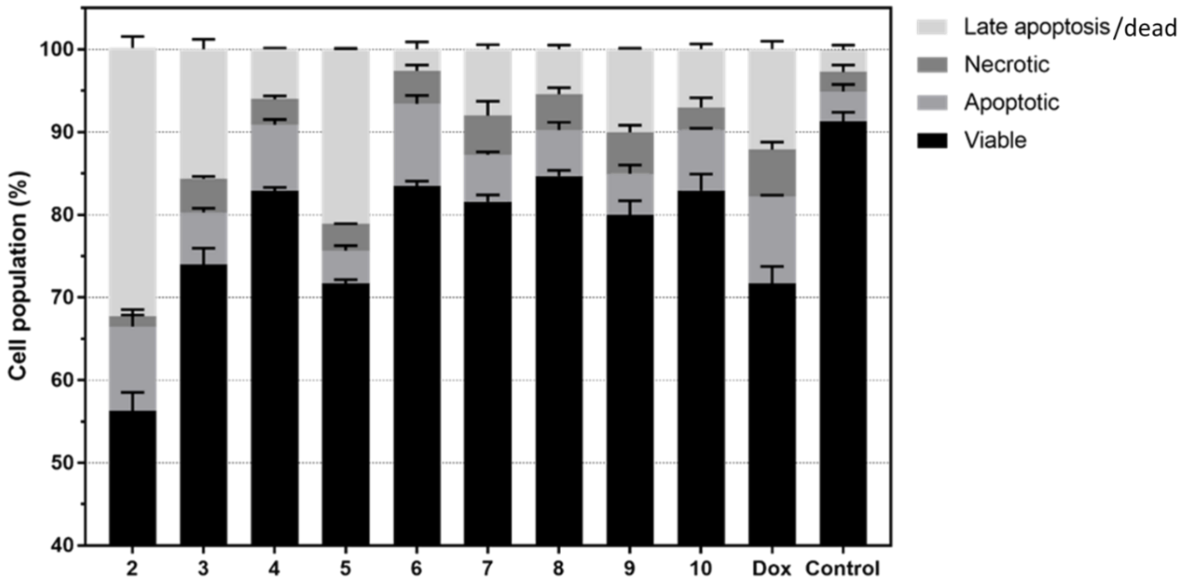

Figure S90. Quantitative analysis of MDA-MB-231-cell death induced by **Ir2** - **Ir9** or doxorubicin at the equitoxic concentrations corresponding to their 2 x $IC_{50,72h}$ determined using annexin-V/PI assay flow cytometry. Error bars are the SDs from three independent experiments.

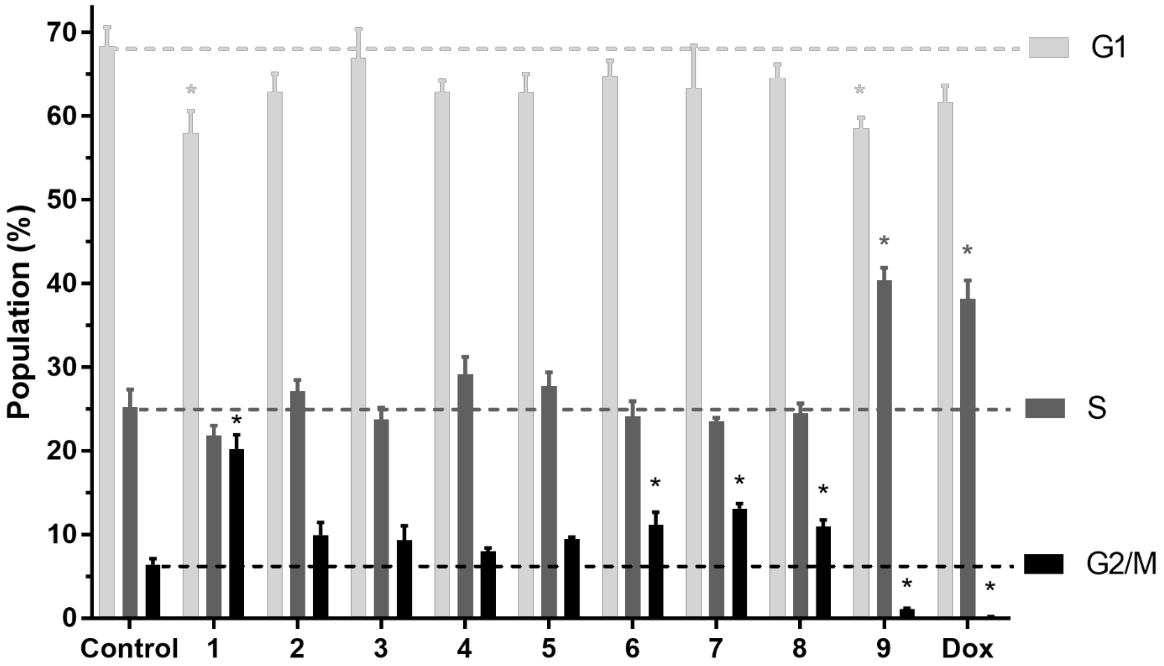

Figure S91. Cell cycle analysis of MDA-MB-231 cells. Cells were treated for 24 h with the concentrations of compounds corresponding to $2xIC_{50,72h}$. Data were analyzed using students´ t-test, and the significant differences ($p < 0.05$) from vehicle control were marked with a star at the top of the significant bars. The dashed horizontal lines indicated the distribution of each cell cycle phase determined for control samples.

8. Reference

1. Yellol, J.; Perez, S. A.; Buceta, A.; Yellol, G.; Donaire, A.; Szumlas, P.; Bednarski, P. J.; Makhloufi, G.; Janiak, C.; Espinosa, A.; Ruiz, J. Novel C,N-cyclometalated benzimidazole ruthenium(II) and iridium(III) complexes as antitumor and antiangiogenic agents: A structure-activity relationship study. *J. Med. Chem.* **2015,** 58, 7310-7327.
